# The natural product brartemicin is a high affinity ligand for the carbohydrate-recognition domain of the macrophage receptor mincle†
†Electronic supplementary information (ESI) available: Detailed experimental procedures and compound characterization data. See DOI: 10.1039/c4md00512k



**DOI:** 10.1039/c4md00512k

**Published:** 2015-01-07

**Authors:** Kristian M. Jacobsen, Ulrik B. Keiding, Lise L. Clement, Eva S. Schaffert, Neela D. S. Rambaruth, Mogens Johannsen, Kurt Drickamer, Thomas B. Poulsen

**Affiliations:** a Chemical Biology Laboratory , Department of Chemistry , Aarhus University , DK-8000 , Aarhus C , Denmark . Email: thpou@chem.au.dk; b Department of Life Sciences , Imperial College , London SW7 2AZ , UK . Email: k.drickamer@imperial.ac.uk; c Department of Forensic Medicine , Bioanalytical Unit , Aarhus University , Brendstrupgaardsvej 100 , 8200 Aarhus N , Denmark

## Abstract

We demonstrate that the natural product brartemicin, a newly discovered inhibitor of cancer cell invasion, is a high-affinity ligand of the carbohydrate-recognition domain (CRD) of the C-type lectin mincle. Recent studies have revealed that mincle is a key macrophage receptor for the mycobacterial virulence factor trehalose dimycolate (TDM), which is a glycolipid component of the mycobacterial cell wall. Major uncertainties, however, remain concerning the mechanism of TDM-binding and subsequent signal transduction as well as interplay of potential co-receptors. Due to the lipid nature of TDM, functional studies are difficult and soluble mincle-ligands are therefore of significant interest. Brartemicin, together with designed analogs also presented in this paper, may thus serve as useful molecular probes for future studies of mincle. Through computational studies, we further provide an insight into the probable mode of binding of brartemicin.

## Abstract 2

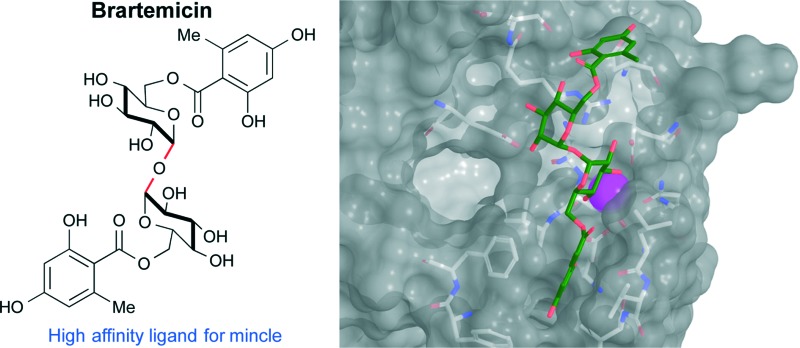
We demonstrate that the natural product brartemicin, a newly discovered inhibitor of cancer cell invasion, is a high-affinity ligand of the carbohydrate-recognition domain (CRD) of the C-type lectin mincle.

## Introduction

**Fig. 1:**
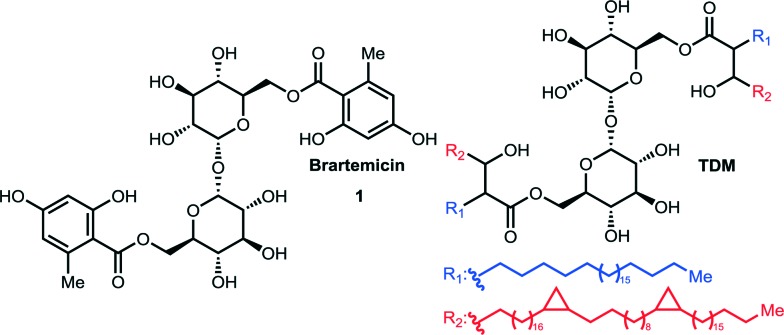
Chemical structures of brartemicin and trehalose-6,6′-dimycolate = TDM. The side chains of TDM displayed are those of the α-mycolic acid subclass.

Trehalose-6,6-dimycolate (TDM, cord factor, Fig. 1) is an abundant component of the mycobacterial cell wall. This complex glycolipid is known to play pivotal roles in the pathogenesis of mycobacteria such as *Mycobacterium tuberculosis*, and it can stimulate granuloma formation *in vivo*.^1^ The biomolecules involved in immune cell activation by TDM has only recently become clear, with the discovery that mincle, macrophage-inducible C-type lectin (CLEC4E), is a key mediator of the recognition of TDM by immune cells.^2^ This cell surface recognition event initiates a signaling cascade through FcRγ-Syk-Card9 running in parallel to signaling pathways downstream of other important pattern-recognition receptors, such as the Toll-Like Receptors (TLRs).^2,3^ Details about the precise molecular mechanism of signal transduction, however, are lacking, and in addition to mincle, a constitutively expressed C-type lectin receptor MCL (CLEC4D) also appears to be intimately involved in the cellular response to TDM and it has been proposed that a mincle-MCL dimer constitutes the functional receptor complex.^4^ As TDM *in vivo* is constrained in the mycobacterial membrane, it is not clear how much of the structure is in fact exposed for interactions with the receptor(s). As a consequence of their immunostimulatory activities, TDM, as well as synthetic analogs, are under investigation as potential vaccine adjuvants^3^ and have further been shown to block tumor formation and metastasis in mice through an adjuvant mechanism.^5^

Brartemicin (**1**, Fig. 1) is a natural product isolated from *Nonomuraea* sp that was recently reported to block matrigel-invasion of cancer cells and which comprises a doubly esterified α,α-trehalose core structure similar to TDM.^6^ A number of ester analogs of brartemicin have recently been reported and their effects upon cancer cell invasion have been studied. However, the direct binding targets of these compounds and the molecular mechanisms by which they work remain obscure.^7^

Realizing the potential importance of discovering high-affinity, non-lipid ligands for mincle, we took note of the significant structural similarity between brartemicin and TDM (Fig. 1). The mycolic acid side chains in TDM are branched at the α-carbon and hydroxylated on the β-carbon, so the part of the structure in proximity to the trehalose core maps closely on to the aromatic groups present in brartemicin. Here we report our initial results demonstrating that brartemicin as well as synthetic analogs are competent binders of mincle.

## Results and discussion

**Fig. 2:**
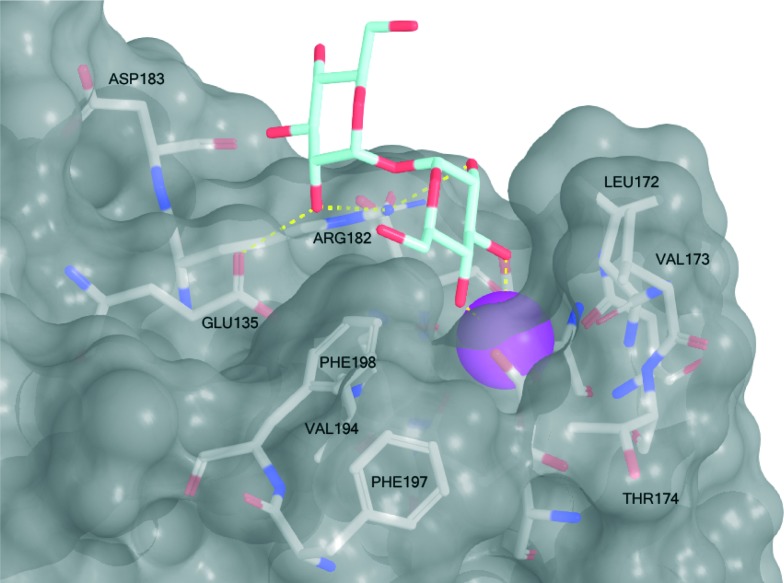
Structure of bovine mincle co-crystallized with α,α-trehalose (PDB entry 4KZV). The Ca^2+^-ion in the primary binding site is coloured magenta. A hydrophobic groove comprised of Leu172, Val173, Val194, Phe197, and Phe198 is shown in the front.

Mincle binds ligands through an extracellular carbohydrate-recognition domain (CRD). A recently published crystal structure of the CRD from bovine mincle in complex with the simple disaccharide α,α-trehalose reveals that one glucose residue is coordinated to a Ca^2+^-ion through the C4 and C5 hydroxyls that anchors the ligand in the primary binding site. The second glucose residue interacts with an adjacent, secondary binding site with *e.g.* Glu135 and Arg182 forming key hydrogen bonds to the C2′-hydroxyl group (Fig. 2).^8^ Moreover, in close vicinity to the Ca^2+^-ion there is a hydrophobic groove that may be involved in recognition of the mycolic acid portions of TDM. Due to the presence of phenylalanine (Phe197 and Phe198) residues we hypothesized that this part of the CRD could potentially interact strongly with ligands containing aromatic groups, such as those found in brartemicin (**1**). In order to investigate the proposed interaction of brartemicin with mincle and to probe key aspects of the structural requirements for binding, the natural product and four additional analogs based on the brartemicin structure were synthesized.

## Synthesis of brartemicin and analogs

**Scheme 1:**
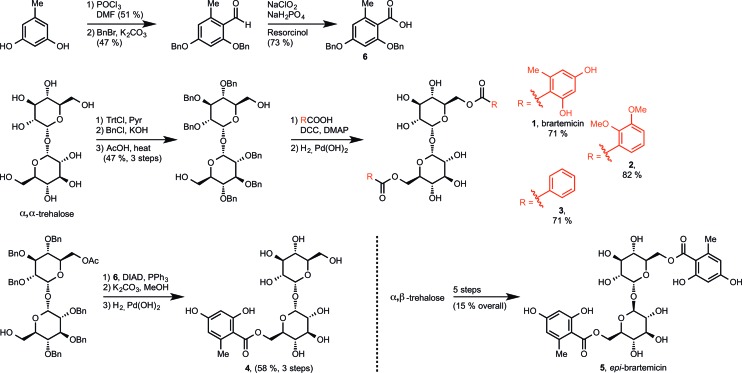
Syntheses of brartemicin (**1**), analogs **2–4**, and *epi*-brartemicin (**5**). For detailed information about reagents and conditions, please see the ESI.† DMF = *N*,*N*-dimethylformamide, Trt-Cl = trityl chloride, Pyr = pyridine, BnCl = benzyl chloride, BnBr = benzyl bromide, DCC = dicyclohexylcarbodiimide, DMAP = 4-dimethylaminopyridine, DIAD = diisopropylazodicarboxylate, PPh_3_ = triphenylphosphine.

We prepared brartemicin (**1**) through a short synthesis sequence from α,α-trehalose (Scheme 1). An orthogonal protection group strategy allowed quick access to the 6,6′-diol that could undergo efficient double ester coupling with carboxylic acid **6** using DCC and DMAP followed by global debenzylation. Carboxylic acid **6** was prepared from orcinol in 3 steps.

Through the same general route, we prepared two analogs **2** and **3**. As a side note, both these compounds were reported to have anti-invasive activity on cancer cells in the same range as brartemicin.^7^ Structurally, these two analogs provide preliminary information about the importance of the substitution pattern on the aromatic rings for mediating binding to the mincle CRD. In order to investigate the importance of the dimeric structure, we prepared monoester derivative **4** starting from the monoacetate of protected α,α-trehalose using a mitsunobu esterification protocol. Finally, we designed a close structural analog, *epi*-brartemicin (**5**), having altered stereochemistry at the glycosidic junction, by starting the synthesis from α,β-trehalose (see experimental section and ESI† for details). This compound would be expected to have a drastically different conformation in the disaccharide moiety, with potential deleterious effects on interaction with the receptor. All compounds were rigorously purified by HPLC before biochemical testing.

## Binding studies of putative ligands to the CRD of mincle

**Fig. 3:**
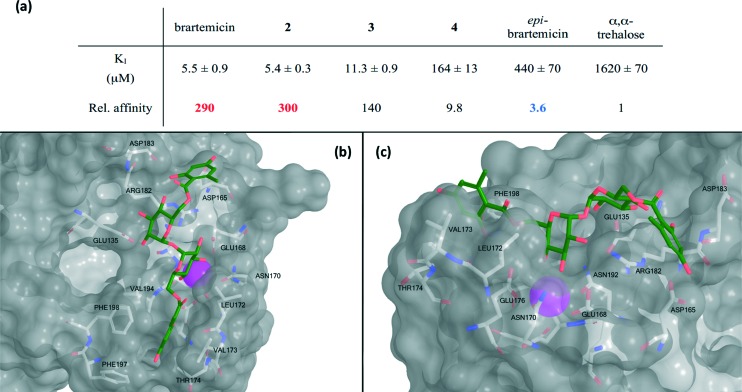
a) Data for inhibition of mannose-conjugated serum albumin binding to the CRD from bovine mincle by brartemicin and analogs. *K*_I_-values (average ± s.d. for three independent experiments) and affinities relative to α,α-trehalose. b) Top view and c) side view of highest scoring docking pose of brartemicin with bovine mincle. The Ca^2+^ is coloured magenta.

The collection of compounds was tested in competition binding experiments with immobilized bovine mincle CRD^8^ (Fig. 3a). Strikingly, we found that brartemicin displayed strong affinity for mincle (*K*_I_ = 5.5 ± 0.9 μM), which is approximately 300-fold higher than the affinity of α,α-trehalose itself. Analog **2** was equipotent (*K*_I_ = 5.4 ± 0.3 μM) and analog **3** had a two-fold reduced affinity (*K*_I_ = 11.3 ± 0.9 μM). Interestingly, removal of one of the ester groups (monoester **4**) resulted in a significant reduction in binding affinity of roughly 30-fold (*K*_I_ = 164 ± 13 μM) compared to brartemicin. This loss in affinity indicates that both ester groups may be directly involved in interactions with the receptor or that one may be required for locking the other ester in a binding-competent conformation.

Finally, *epi*-brartemicin shows an almost 100-fold loss in affinity (*K*_I_ = 440 ± 70 μM) compared to natural brartemicin and thus has only marginally higher affinity than α,α-trehalose. This observation indicates strong specificity of the binding site in mincle for the α,α-trehalose-core structure. Importantly, we found that brartemicin also binds with high affinity to the CRD of human mincle^9^ (Fig. S1†).

## Computational studies of brartemicin and *epi*-brartemicin

To deepen the understanding of the binding mode of these new, non-lipid mincle ligands we inspected the crystal structure of bovine mincle co-crystallized with α,α-trehalose (PDB entry 4KZV), in which both rings of α,α-trehalose make contacts with the receptor (Fig. 2). The crystal structure was prepared for modeling by Protein Preparation Wizard in Maestro. We then performed molecular docking of brartemicin in bovine mincle using the Glide algorithm,^10^ and identified a population of binding capable conformations (Fig. 3b, c and Fig. S2a†). In these conformations the disaccharide core of brartemicin was found to overlap closely with the crystal structure of α,α-trehalose in complex with mincle (Fig. S3†). Furthermore, as initially predicted, the calculations provide strong support for hydrophobic interactions between one of the aromatic esters of brartemicin and the side chains of residues Leu172, Val173, Val194, Phe197, and Phe198 that together comprise the hydrophobic groove adjacent to the Ca^2+^-ion. The size of the aromatic ring is closely matched with the size of the groove. The second aromatic ester is oriented in the opposite direction within the binding site in the vicinity of Arg182 indicating possible π-cation interactions and in the vicinity of Asp165/Asp183 where polar contacts are possible (Fig. 3b, c). Overall the calculations suggest a possible position of the second ester substituent and an overall elongated conformation of brartemicin in the binding site. The above mentioned interactions can explain the 290-fold increase in binding affinity of brartemicin compared to α,α-trehalose.

Docking of *epi*-brartemicin (**5**) and the monoester (**4**) under the same conditions as **1** suggests subtle changes in the bound conformations, which could explain the reduced affinity of these compounds. Whereas the ensemble of poses found for brartemicin (Fig. S2a†) show a very well defined binding mode, the ensembles of poses for both monoester (**4**) and *epi*-brartemicin (Fig. S2b–c†) are much more diffuse suggesting that key stabilizing interactions are diminished. In both these structures, the glucose ring in the secondary binding site is rotated significantly relative to the brartemicin structure. Most notably, the altered glycosidic configuration in *epi*-brartemicin is incompatible with an elongated conformation in the binding site and key hydrogen bonds are absent. Surprisingly, the computational studies of the monoester (**4**) indicate that in this molecule the aromatic ester occupies an alternative cavity in close vicinity to the hydrophobic groove (Fig. S2c†). The reduced interactions in these structures reflect in lower docking scores in accord with the relative affinities (Table S1, Fig. S4†).

Overall, our experiments and computational studies indicate that brartemicin and *epi*-brartemicin constitute a pair of molecules that will allow affinity correlations in future functional assays. The structural requirements for immune cell activation by TDM-mimetics are not well understood and the situation is further complicated by the presence of additional receptors for TDM, such as MCL, which directly impact the expression of mincle.^11^ At the cytokine level, TDM-analogs with simple saturated ester groups display disparate behaviour and seem to show maximum activation with intermediate length chains.^12^ Due to the highly amphiphilic nature of these compounds, solubility issues are unavoidable and may complicate interpretation of some experiments. Our discovery that phenol-containing esters, like brartemicin, are strong binders of mincle CRD that are soluble at millimolar concentrations in aqueous media, will enable the preparation of new classes of soluble TDM analogs that may help shed light on the mechanism involved in immune cell activation through the mincle pathway.

## Experimental

### Synthesis

#### 2,3,4,2′,3′,4′-Hexa-(benzyloxy)-6,6′-bis-(2,4-bis-(benzyloxy)-6-methylbenzoate)-α,α-trehalose

An azeotrope was formed with the benzyl-protected α,α-trehalose (50 mg, 0.06 mmol, 1.0 equiv.) and 2,4-bis-(benzyloxy)-6-methylbenzoic acid (43 mg, 0.12 mmol, 2.2 equiv.) in dry benzene and this was concentrated to dryness. This was repeated three times. The residue was then dissolved in dry DCM (0.24 mL) in a flame-dried schlenk flask containing a stirring bar and flushed with Ar. The mixture was cooled to 0 °C and DCC (0.03 g, 0.14 mmol, 2.4 equiv.) and a catalytic amount of DMAP was added. The reaction was allowed to reach rt and was stirred overnight under an argon atmosphere. After 26 h the reaction mixture was filtered to remove the urea product of DCC. The filtrate was concentrated *in vacuo*. Purification of the crude product by FC (SiO_2_, 15 × 2 cm, EtOAc/pentane 1 : 5 → EtOAc/pentane 1 : 4 → EtOAc/pentane 1 : 2) yielded the diester (62 mg, 0.04 mmol, 71%) as a colorless oil. *R*_f_ 0.50 (EtOAc/pentane 1 : 5 (CAM-stain)). ^1^H NMR (400 MHz, CDCl_3_) *δ* 7.39–7.14 (m, 50H), 6.36 (d, *J* = 1.8 Hz, 2H), 6.31 (d, *J* = 1.8 Hz, 2H), 5.10 (d, *J* = 3.5 Hz, 2H), 5.02–4.92 (m, 12H), 4.83–4.74 (m, 4H), 4.57–4.48 (m, 8H), 4.26 (d, *J* = 10.2 Hz, 4H), 4.00 (t, *J* = 9.6 Hz, 2H), 3.59 (t, *J* = 9.6 Hz, 2H), 3.43 (dd, *J* = 3.5, 9.6 Hz, 2H), 2.24 (s, 6H). ^13^C NMR (100 MHz, CDCl_3_) *δ* 168.1, 160.4, 157.2, 138.9, 138.4, 138.2, 138.0, 136.7, 136.6, 128.7, 128.6, 128.5, 128.4, 128.4, 128.2, 128.2, 127.9, 127.8, 127.7, 127.6, 127.5, 126.8, 117.2, 108.2, 98.5, 93.7, 81.5, 79.2, 77.9, 75.5, 75.3, 72.6, 70.1, 69.4, 63.2, 20.1. IR (neat) *ν*_max_/cm^–1^ 3031, 2926, 2864, 1725, 1602, 1586, 1497, 1453, 1263, 1157, 1089, 1070, 995, 733, 698. [*α*]26.8D = +55.2 (c 0.50, CHCl_3_).

#### 6,6′-Bis-(2,4-dihydroxy-6-methylbenzoate)-α,α-trehalose (**1**)

The benzyl-protected brartemicin (33.1 mg, 21.4 μmol, 1.0 equiv.) was dissolved in MeOH/CHCl_3_ 1 : 1 (5 mL) in a 50 mL round-bottom flask and the flask was then purged with argon. To the solution was added Pd(OH)_2_/C (23 mg, 20%, 32 μmol, 1.5 equiv.) and the mixture was stirred while flushed with argon. The atmosphere in the flask was subsequently saturated with H_2_-gas from a balloon. After 6 h the reaction had run to completion as judged by TLC and the mixture was filtered to remove residues of the catalyst. The solvents were removed *in vacuo* to yield brartemicin (**1**) as an analytically pure off-white solid (13.5 mg, 21.0 μmol, 98%). The product was purified further by semi-preparative C-18 RP HPLC prior to binding assays (5% → 70% MeOH in H_2_O over 17 min, hold 3 min, then 70% → 100% MeOH, hold 1 min, 10 mL min^–1^, RT = 16.7 min, Phenomenex Luna 5u C18(2) 100 A, New Column, 250 × 10 mm). *R*_f_ 0.58 (EtOAc/MeOH/H_2_O 4 : 1 : 1 (CAM-stain)). ^1^H NMR (400 MHz, CD_3_OD) *δ* 6.21 (d, *J* = 2.3 Hz, 2H), 6.15 (d, *J* = 2.3 Hz, 2H), 5.13 (d, *J* = 3.7 Hz, 2H), 4.58 (dd, *J* = 2.0, 12.0 Hz, 2H), 4.46 (dd, *J* = 4.9, 12.0 Hz, 2H), 4.21–4.17 (m, 2H), 3.83 (t, *J* = 9.6 Hz, 2H), 3.49 (dd, *J* = 3.7, 9.6 Hz, 2H), 3.43 (t, *J* = 9.6 Hz, 2H) 2.51 (s, 6H). ^13^C NMR (100 MHz, CD_3_OD) *δ* 172.8, 166.3, 163.9, 144.9, 112.5, 105.6, 101.7, 95.6, 74.5, 73.2, 72.2, 71.3, 65.4, 24.9. HRMS (*m*/*z*): [M–H]^–^ calcd for C_28_H_33_O_17_, 641.1723; found, 641.1723. IR (neat) *ν*_max_/cm^–1^ 3292, 2928, 2856, 1644, 1617, 1447, 1312, 1256. [*α*]26.5D = +78.8 (c 0.50, MeOH).

#### 2,3,4,2′,3′,4′-Hexa-(benzyloxy)-6,6′-bis-(2,4-bis-(benzyloxy)-6-methylbenzoate)-α,β-trehalose

The hexabenzylated α,β-trehalose (0.050 g, 56.7 μmol, 1.0 equiv.) was added to a flame-dried flask and an azeotrope was formed with dry benzene, concentrated to dryness and subsequently placed under high vacuum. This step was repeated once. The flask was purged with argon and the compound was redissolved in dry THF/toluene (2 mL, 1 : 1) and to this was added 2,4-bis-(benzyloxy)-6-methylbenzoic acid (0.059 g, 170 μmol, 3.0 equiv.) and PPh_3_ (0.045 g, 170 μmol, 3.0 equiv.). The mixture was cooled to 0 °C in an ice bath. To the cooled solution was added DIAD (33 μL, 170 μmol, 3.0 equiv.) the reaction was stirred at 0 °C for 2.5 h until TLC showed full conversion of starting material. The reaction mixture was diluted with ice-water and extracted with EtOAc (3×), after which the organic phases were washed with brine, dried over Na_2_SO_4_ and concentrated *in vacuo*. The crude product was purified by FC (SiO_2_, 2 × 11 cm, EtOAc/pentane 10% → 20% → 25% → 30%). The product was isolated as a colorless oil (0.043 g, 27 μmol, 48%). *R*_f_ 0.71 (EtOAc/heptane 1 : 1 (CAM-stain)). ^1^H NMR (400 MHz, CDCl_3_) *δ* 7.41–7.08 (m, 50H), 6.36 (d, *J* = 2.1 Hz, 1H), 6.34 (d, *J* = 2.2 Hz, 1H), 6.29 (d, *J* = 2.1 Hz, 2H), 5.11–5.02 (m, 4H), 5.02–4.98 (m, 1H), 4.98–4.91 (m, 5H), 4.87 (t, *J* = 10.8 Hz, 2H), 4.79–4.67 (m, 6H), 4.59–4.44 (m, 6H), 4.39 (dd, *J* = 12.0, 3.9 Hz, 1H), 4.29–4.18 (m, 2H), 3.96 (t, *J* = 9.3 Hz, 1H), 3.66 (t, *J* = 9.5 Hz, 1H), 3.62–3.56 (m, 2H), 3.53–3.47 (m, 1H), 3.46–3.35 (m, 2H), 2.23 (s, 3H), 2.20 (s, 3H). ^13^C NMR (100 MHz, CDCl_3_) *δ* 167.9, 167.8, 160.3, 160.3, 157.0, 157.0, 138.7, 138.5, 138.5, 138.4, 138.3, 137.9, 137.8, 136.8, 136.7, 136.6, 136.5, 128.6, 128.6, 128.5, 128.5, 128.4, 128.4, 128.3, 128.2, 128.1, 128.0, 128.0, 127.8, 127.7, 127.7, 127.6, 127.6, 127.5, 127.5, 127.5, 127.4, 127.3, 126.7, 126.6, 117.1, 116.8, 108.0, 107.9, 103.7, 99.1, 98.4, 98.3, 84.3, 81.6, 81.5, 79.6, 77.5, 75.6, 75.3, 75.1, 75.0, 74.4, 73.3, 73.0, 70.1, 70.0, 70.0, 69.9, 20.1, 20.0. HRMS (*m*/*z*): [M + H]^+^ calcd for C_98_H_95_O_17_, 1543.6564; found, 1543.6603. IR (neat) *ν*_max_/cm^–1^ 3031, 2924, 1725, 1602, 1497, 1453, 1325, 1263, 1158, 1072, 1043, 1027, 909, 732, 695.

#### 6,6′-Bis-(2,4-dihydroxy-6-methylbenzoate)-α,β-trehalose (**5**)

The globally benzylated *epi*-brartemicin (0.042 g, 27 μmol, 1.0 equiv.) was dissolved in CHCl_3_/MeOH (1 : 1, 5 mL) and the flask was purged with argon before Pd(OH)_2_/C (0.020 g, 20%, 28 μmol, 1.05 equiv.) was added. The flushing with argon was continued until the atmosphere in the flask was exchanged for H_2_-gas. The reaction mixture was stirred under the H_2_-atmosphere for 4 h at which point TLC showed full conversion of the starting material. The mixture was filtered over celite with MeOH and solvents were evaporated. This yielded the product quantitatively as an analytically pure brownish solid. The product was subsequently purified by semi-preparative HPLC before binding assays (5% → 65% MeCN in H_2_O over 20 min, hold for 1 min, then 65% → 100% MeCN, hold for 2 min, RT = 5.5 min, 10 mL min^–1^, Phenomenex Luna 5u CN 100 A, 250 × 10 mm 5 micron). *R*_f_ 0.58 (EtOAc/MeOH/H_2_O 4 : 1 : 1 (CAM-stain)). ^1^H NMR (400 MHz, CD_3_OD) *δ* 6.19–6.03 (m, 4H), 5.12 (d, *J* = 3.7 Hz, 1H), 4.61–4.49 (m, 2H), 4.48–4.35 (m, 2H), 4.28 (dd, *J* = 11.9, 2.6 Hz, 1H), 4.06 (dt, *J* = 10.2, 2.9 Hz, 1H), 3.72–3.57 (m, 2H), 3.50–3.35 (m, 5H), 2.48 (s, 3H), 2.43 (s, 3H). ^13^C NMR (100 MHz, CD_3_OD) *δ* 172.7, 172.3, 166.3, 166.1, 163.9, 163.7, 144.7, 112.6, 112.5, 105.9, 102.8, 101.7, 77.4, 75.5, 75.3, 74.9, 73.8, 71.6, 71.5, 65.2, 64.4, 24.9, 24.8. HRMS (*m*/*z*): [M – H]^–^ calcd for C_28_H_33_O_17_, 641.1723; found, 641.1725. IR (neat) *ν*_max_/cm^–1^ 3391, 2920, 2851, 1645, 1456, 1315, 1263, 1167, 1080.

### Binding studies

A biotin-tagged version of the CRD from bovine mincle was prepared in a bacterial expression system as previously described.^8^ An equivalent fragment of human mincle was generated using an analogous system.^9^ Binding competition assays, based on inhibition of binding of radioiodinated Man_31_-bovine serum albumin to the biotin-tagged CRDs immobilized on streptavidin-coated plates, were performed as previously described.^8^ Results are reported as average ± standard deviation for at least 3 independent experiments, each performed in duplicate.

### Docking experiments

The computational studies were performed with a crystal structure of bovine mincle (PDB entry 4KZV). The pdb file was loaded in the Schrödinger (2014-2) software Maestro 9.8^13^ and prepared using Protein Preparation Wizard^14^ in accordance with Schrödinger's guidelines. The structures of brartemicin and *epi*-brartemicin were loaded into the software and minimized.^15^ Using Glide 5.6,^16^ a ligand binding domain was defined at the trehalose binding site and the minimized structures were docked generating up to 25 poses of each structure in the binding site. The structures were scored using Glide XP algorithm.

## Conclusions

In conclusion, we have discovered that the natural product brartemicin is a new ligand for the CRD of mincle. The affinity is higher than that of long chain alkyl (C6) diesters^8^ and thus brartemicin constitutes a new lead structure for identifying soluble mincle-ligands with even higher affinity for this receptor. We also report that inverting one stereogenic center at the core trehalose leads to a dramatic reduction in affinity. We speculate that the molecules reported here will constitute important molecular probes for future studies of mincle and its role in innate immunity.
