# Supplementary material for: The natural product brartemicin is a high affinity ligand for the carbohydrate-recognition domain of the macrophage receptor mincle
Source: Medchemcomm. 2015 Jan 7;6(4):647–52. doi: 10.1039/c4md00512k (PMC4393326; doi:10.1039/c4md00512k)
Supplement: Supplementary file 1 [file MD-006-C4MD00512K-s001.pdf]

Electronic Supplementary Material (ESI) for MedChemComm.
This journal is © The Royal Society of Chemistry 2015

The Natural Product Brartemicin is a High Affinity Ligand for the Carbohydrate-Recognition Domain of the Macrophage Receptor Mincle

Supporting Information

Kristian M. Jacobsen,[a] *Ulrik B. Keiding,*[a,c] *Lise L. Clement,*[a] *Eva S. Schaffert,*[a] *Neela D. S. Rambaruth,*[b] *Mogens Johannsen,*[c] *Kurt Drickamer,*[b#*] *and Thomas B. Poulsen.*[a#*]

[a] Chemical Biology Laboratory, Department of Chemistry, Aarhus University, DK-8000, Aarhus C, Denmark,

[b] Department of Life Sciences, Imperial College, London SW7 2AZ, United Kingdom,

and

[c] Department of Forensic Medicine, Bioanalytical Unit, Aarhus University, Brendstrupgaardsvej 100, 8200 Aarhus N, Denmark.

[#] These authors contributed equally to this work.

[*] thpou@chem.au.dk, k.drickamer@imperial.ac.uk

Contents

General synthetic methods 2

Biological methods 3

Computational studies 3

Supplementary Figures 4

Synthesis 8

References 20

Spectra 21

HPLC-UV-MS Chromatograms 39

General synthetic methods

All solvents were of HPLC-grade quality and commercially available from Sigma-Aldrich or VWR and were used without further treatment unless stated otherwise. For the reactions performed under inert atmosphere dichloromethane was dried prior to use over aluminum oxide via a Braun solvent purification system. Benzene and pyridine were dried over activated molecular sieves (4 Å).[A] Anhydrous DMF was purchased from Sigma-Adrich. Reagents were used as received from commercial suppliers. Reactions with air- and moisture sensitive compounds were conducted in flame-dried glassware under an atmosphere of argon. Concentration *in vacuo* was done using a rotary evaporator with the water bath temperature at 40 °C, followed by further concentration using a high vacuum pump. TLC analysis was carried out on silica coated aluminum foil plates (Merck Kieselgel 60 F254). The TLC plates were visualized by UV light and/or by staining with either CAM-stain ($(NH_4)_6Mo_7O_{24}$ $4H_2O$ (10 g), Ceric ammonium sulfate (4 g), 10% H_2SO_4 (aq., 400 mL)), or $KMnO_4$-stain ($KMnO_4$ (5.0 g), 5% NaOH (aq., 8.3 mL) and K_2CO_3 (33.3 g) in H_2O (500 mL)) followed by successive heating. Flash column chromatography was carried out using silica gel (230-400 Mesh particle size, 60 Å pore size) as stationary phase. Dry column chromatography was carried out using Merck silica gel 60 (0.015 – 0.040 mm) as stationary phase. End products were purified by a Gilson GX271/H-322 HPLC system linked to a Gilson UV/VIS-155 detector and a PerkinElmer Flexar SQ 300 MS Detector.

^{1}H and ^{13}C NMR spectra were recorded on either a Varian Mercury spectrometer or a Bruker BioSpin GmbH spectrometer at 400 MHz. The spectra were interpreted on the basis of DEPT-135, *g*COSY and *g*HMQC techniques when necessary. All chemical shifts were reported in ppm relative to the solvent peak. Singlets will be reported as s, doublets as d, triplets as t, and multiplets as m. Doublets of a doublet and doublets of a triplet will be reported as dd and dt, respectively. Coupling constants will be reported as *J* and are given in Hz. Mass spectrometry was measured on a Bruker Daltonics MicrOTOF time-of-flight spectrometer with electrospray ionization. MS-spectra were analyzed in Bruker Compass DataAnalysis 4.1 and calibrated using an internal standard of sodium formate clusters. IR spectroscopy was performed on PerkinElmer Spektrum TwoTM UATR spectrometer. Optical rotation was obtained on an ADP 440+ Polarimeter from Bellingham and Stanley. Melting points were measured on a Büchi B-540 instrument and the melting points are reported as uncorrected values. All yields are corrected for eventual residues of solvent based on ^{1}H NMR integrals.

[A] Activation was achieved by pre-drying 4 Å molecular sieves at 120 °C overnight and subsequently heating them in a microwave oven at 600 W for 1 minute. The latter was done repeatedly until complete dryness was obtained.

Biological methods

A biotin-tagged version of the CRD from bovine mincle was prepared in a bacterial expression as previously described.[1] An equivalent fragment of human mincle was generated using an analogous system.[2] Binding competition assays, based on inhibition of binding of radioiodinated Man31-bovine serum albumin to the biotin-tagged CRDs immobilized on streptavidin-coated plates, were performed as previously described.[1] Results are reported as average ± standard deviation for at least 3 independent experiments, each performed in duplicate.

Computational studies

Of the poses given by the Glide 6.3[B] calculation the poses with the lowest internal energy, judged by the glide emodel value, were chosen for comparison. These poses were selected as emodel is related to calculated internal energy and they therefore are likely to represent the most populated conformations of the given molecule, especially at lower temperatures. The poses were qualitatively compared, as judged by eye, for possible explanations of the differences in affinity seen in the competition experiment. In all poses found, brartemicin scored a higher XP GScore than any poses of either *epi*-brartemicin (**5**) or monoester (**4**), which again supports the results from the competition assay.

[B] Glide, version 6.3, Schrödinger, LLC, New York, NY, 2014.

Supplementary Figures

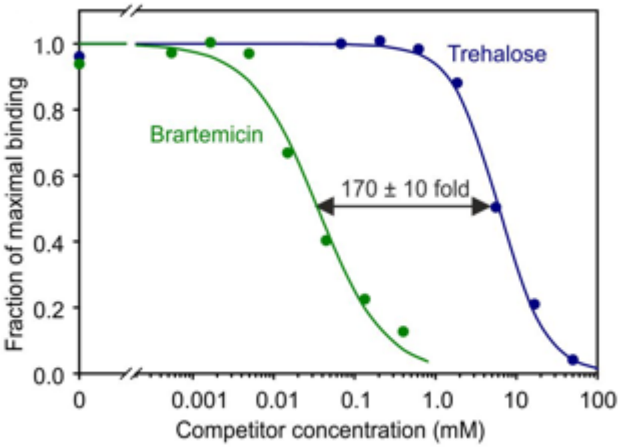

Figure S1. Representative inhibition curves for brartemicin and α,α-trehalose competing with radiolabelled mannose-conjugated serum albumin for binding to the CRD from human mincle.

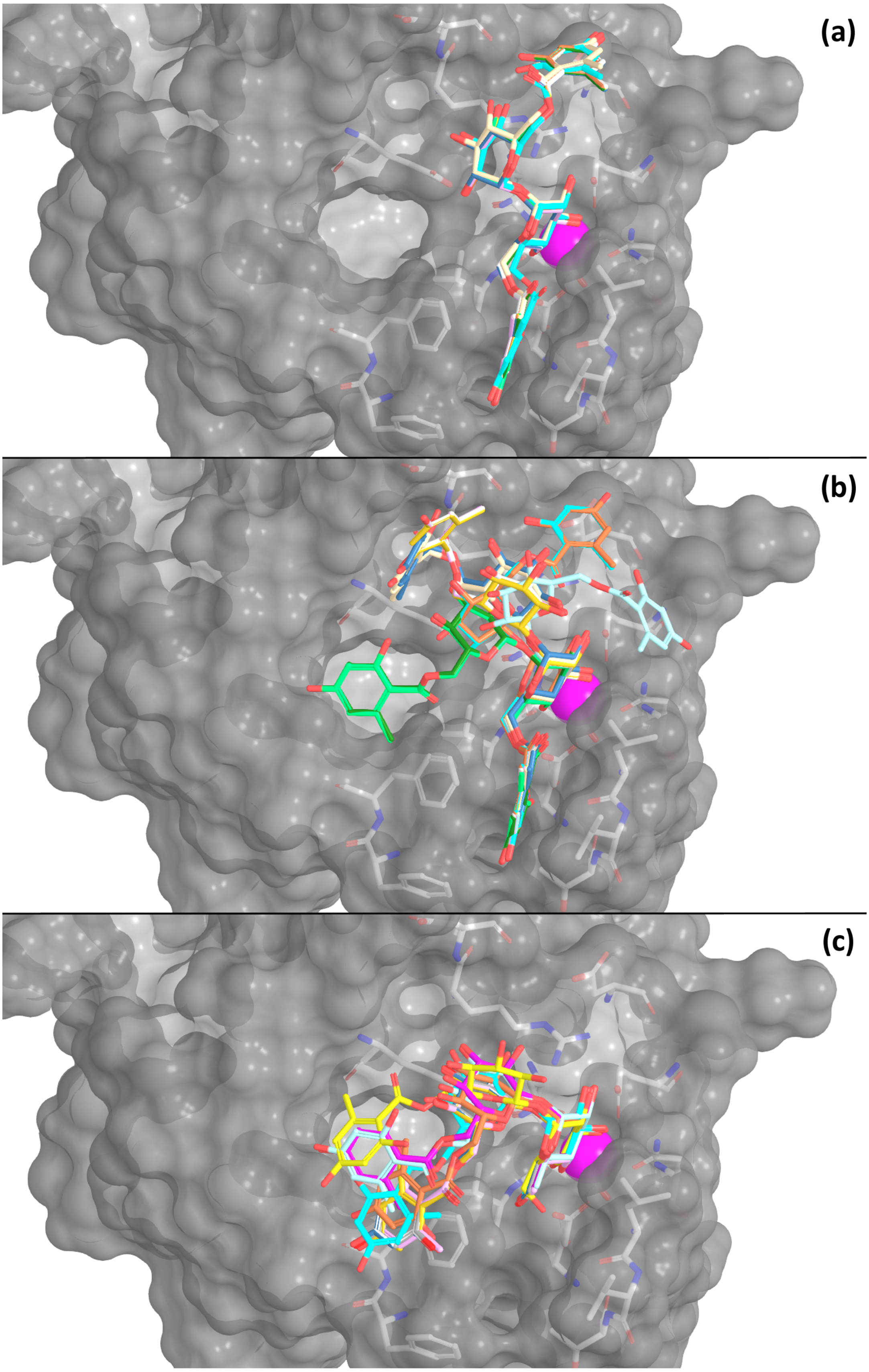

Figure S2. Complete ensemble of docking poses of a) brartemicin, b) *epi*-brartemicin, c) monoester (**4**).

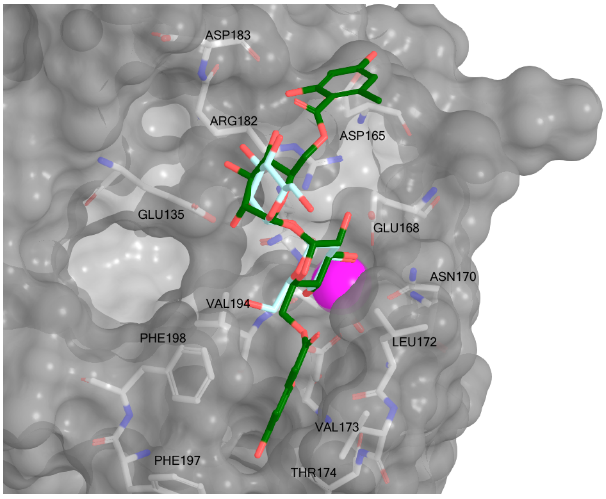

Figure S3. Highest scoring docking pose for brartemicin (green) overlayed with the α,α-trehalose (cyan) ligand co-crystallized with bovine mincle (PDB entry 4KZV). The Ca^{2+}-ion is coloured magenta.

Table S1. Docking scores for brartemicin, monoester (**4**) and epi-brartemicin

Structure	Docking score (kcal/mol)	Structure	Docking score (kcal/mol)	Structure	Docking score (kcal/mol)
Brartemicin	-8.645	**4**	-7.484	*epi*-bratemicin	-7.173
Brartemicin	-8.542	**4**	-7.290	*epi*-bratemicin	-7.133
Brartemicin	-8.480	**4**	-6.738	*epi*-bratemicin	-6.888
Brartemicin	-8.476	**4**	-6.733	*epi*-bratemicin	-6.772
Brartemicin	-8.466	**4**	-6.239	*epi*-bratemicin	-6.656
Brartemicin	-8.345	**4**	-6.237	*epi*-bratemicin	-6.656
Brartemicin	-8.208	**4**	-6.198	*epi*-bratemicin	-6.597
Brartemicin	-8.006	**4**	-5.986	*epi*-bratemicin	-6.567
		**4**	-5.916	*epi*-bratemicin	-6.321
		**4**	-5.330	*epi*-bratemicin	-6.307

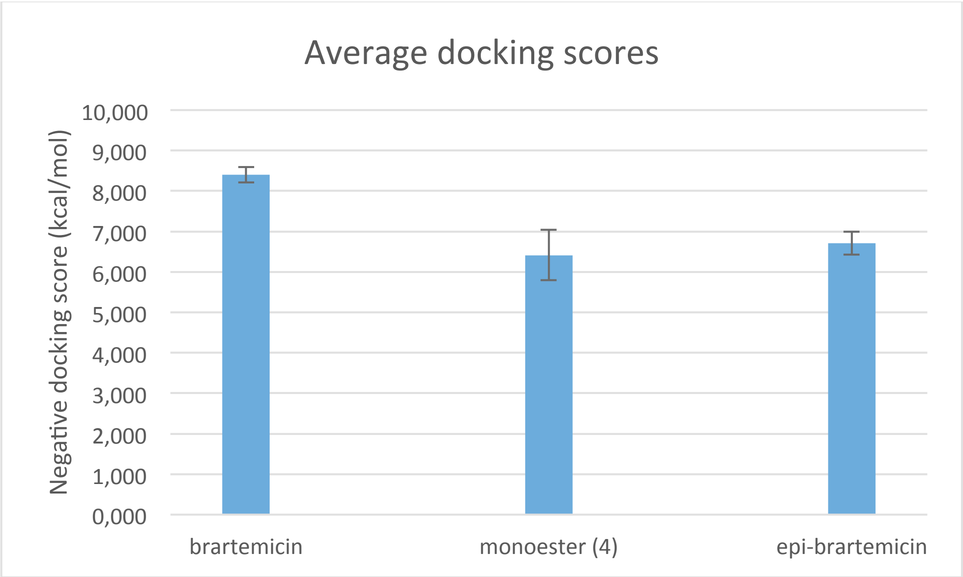

Figure S4. Average docking scores of brartemicin, monoester (**4**), and epi-brarte*micin.*

Synthesis

2,4-Dihydroxy-6-methylbenzaldehyde

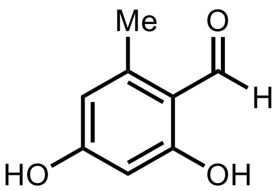

Phosphoryl chloride (9.8 mL, 110 mmol, 2.1 equiv.) was diluted in dry DMF (25 mL) in a flame-dried 50 mL round-bottom flask and subsequently transferred to a flame-dried 250 mL round-bottom flask containing a magnetic stirring bar. The solution was cooled to 10 °C and a solution of 3,5-dihydroxy toluene (6.22 g, 50.1 mmol, 1.0 equiv.) in dry DMF (21 mL) was added dropwise. The reaction mixture was left to stir for 17.5 h at rt. The mixture was cooled to 0 °C and treated with ice water (70 mL). The pH-value of the mixture was adjusted to ≈ 10 using NaOH (10 % in water). The mixture was refluxed for 10 min, then cooled and acidified with HCl (conc.) to pH ≈ 3. The solids were filtered off, washed with water, and dried *in vacuo*. The reaction yielded the aldehyde as a yellow powder (3.85 g, 25.3 mmol, 51%). R_f 0.65 (EtOAc/Pentane 1:1 ($KMnO_4$-stain)). ^{1}H NMR (400 MHz, DMSO-d_6) δ 12.07 (s, 1H), 10.67 (s, 1H), 10.04 (s, 1H), 6.20 (s, 1H), 6.12 (s, 1H), 2.44 (s, 3H). ^{13}C NMR (100 MHz, DMSO-d_6) δ 192.9, 165.4, 165.2, 144.7, 112.6, 110.7, 100.3, 18.5. HRMS (*m/z*): $[M-H]^-$ calcd for $C_8H_7O_3$, 151.0401; found, 151.0403. IR (neat) ν_{max}/cm^{-1} 3011, 1623, 1593, 1479, 1458.

2,4-Bis-(benzyloxy)-6-methylbenzaldehyde

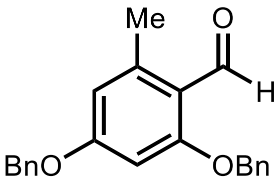

In a 250 mL round-bottom flask a solution of the aromatic aldehyde (2.00 g, 13.1 mmol, 1.0 equiv.) and BnBr (3.9 mL, 33 mmol, 2.5 equiv.) was made in acetone (70 mL). K_2CO_3 (7.27 g, 52.6 mmol, 4.0 equiv.) was added to the mixture and the mixture was refluxed for 2.5 h. Then the reaction mixture was left to cool to rt. The crude mixture was filtered under suction and the solvents were evaporated *in vacuo*. The crude product was purified by FC (SiO_2, Et_2O/Pentane 1:5 → 1:4) Purification gave the product as a pale green solid (2.04 g, 6.13 mmol, 47%). R_f 0.65 (EtOAc/Pentane 3:7 (CAM stain)). ^{1}H NMR (400 MHz, $CDCl_3$) δ 10.60 (s, 1H), 7.42-7.34 (m, 10H), 6.48 (d, J = 2.1 Hz, 1H), 6.44-6.43 (m, 1H), 5.11 (s, 2H), 5.09 (s, 2H), 2.60 (s, 3H). ^{13}C NMR (100 MHz, $CDCl_3$) δ 190.7, 164.4, 163.6, 144.8, 136.2, 128.9, 128.8, 128.5, 128.4, 127.7, 127.4, 117.9, 110.2, 98.0, 70.7, 70.3, 22.5. HRMS (*m/z*): $[M-H]^-$ calcd for $C_{22}H_{19}O_3$, 331.1340; found, 331.1339. IR (neat) ν_{max}/cm^{-1} 3100, 2978, 2866, 2775, 1686, 1593, 1573, 1500, 1443, 1375, 1319, 1220, 1150, 1038.

2,4-Bis-(benzyloxy)-6-methylbenzoic acid (6)

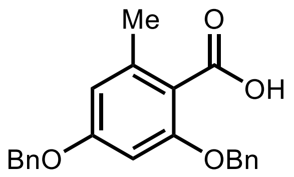

2,4-Bis-(benzyloxy)-6-methylbenzaldehyde (0.17 g, 0.50 mmol, 1.0 equiv.) was added to a 50 mL round-bottom flask containing a magnetic stirring bar before it was dissolved in *t*-BuOH (7 mL) and the reaction flask was flushed with argon. In order to solubilize the compound the mixture was sonicated at 40 °C until the aldehyde was fully dissolved. Subsequently, resorcinol (1.65 g, 15.0 mmol, 30.1 equiv.) was added to the mixture and left stirring for a few minutes to dissolve part of the resorcinol. In parallel $NaClO_2$ (0.11 g, 1.21 mmol, 2.4 equiv.) and NaH_2PO_4 (0.36 g, 3.00 mmol, 6.0 equiv.) were dissolved in water (1.7 mL). This mixture was added dropwise to the aldehyde mixture over 45 minutes. The resulting reaction mixture was stirred under an argon atmosphere for 5.5 h after which the crude reaction mixture was concentrated to dryness and redissolved in DCM. The crude mixture was washed with aq. HCl (10%) and the aqueous phase was back-extracted three times with DCM. The combined organic phases were washed five times with brine, dried over anhydrous Na2SO4, and concentrated *in vacuo*. The product was purified by FC (SiO_2, 10x3 cm, EtOAc/pentane 1:8 + 2% acetic acid). Purification gave the acid as a greyish solid (0.13 g, 0.36 mmol, 73%). R_f 0.68 (EtOAc/pentane 7:3 (CAM stain)). ^{1}H NMR (400 MHz, $CDCl_3$) δ 7.41-7.34 (m, 10H), 6.56 (d, J = 2.2 Hz, 1H), 6.54 (d, J = 2.2 Hz, 1H), 5.16 (s, 2H), 5.08 (s, 2H), 2.60 (s, 3H). ^{13}C NMR (100 MHz, $CDCl_3$) δ 169.7, 161.2, 158.4, 142.7, 136.3, 135.7, 128.8, 128.7, 128.3, 128.2, 127.5, 127.4, 113.9, 110.0, 98.7, 71.4, 70.1, 22.0. HRMS (*m/z*): $[M-H]^-$ calcd for $C_{22}H_{19}O_4$, 347.1289; found 347.1292. IR (neat) ν_{max}/cm^{-1} 3034, 2915, 2868, 1681, 1605, 1580, 1498, 1447, 1327, 1296, 1170, 1053, 802, 724, 690.

6,6'-Di-(trityloxy)-α,α-trehalose

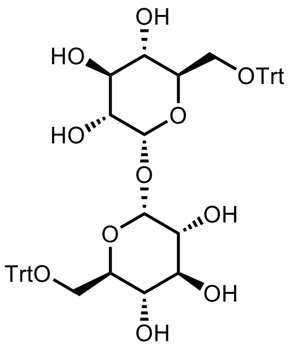

To a 1 L flame-dried round-bottom flask containing a magnetic stirring bar was added α,α-trehalose (20.8 g, 54.8 mmol, 1.0 equiv.) and an azeotrope was formed with dry pyridine (360 mL) and the mixture was concentrated to dryness. Subsequently the α,α-trehalose was redissolved in dry pyridine (160 mL) and TrtCl (38.2 g, 137 mmol, 2.5 equiv.) was added. The resulting mixture was stirred at rt for 17.5 h after which ice water (225 mL) was added and a colorless gum precipitated. Ethanol (70 mL) and heptane (30 mL) was added to turn the gum into a solid that could be filtered off. The mixture was filtered under suction, washed with water, extracted with benzene and concentrated to dryness. The colorless solid was triturated with absolute EtOH (500 mL) and filtered under suction, which yielded the product as a colorless powder (33.6 g, 54.8 mmol, 74%). R_f 0.67 (EtOAc/MeOH/H_2O 4:1:1 (CAM-stain)). ^{1}H NMR (400 MHz, DMSO-d_6) δ 7.47-7.22 (m, 30H), 5.18 (d, J = 3.6 Hz, 2H), 4.08 (m, 2H), 3.65 (t, J = 9.3 Hz, 2H), 3.42 (dd, J = 3.6, 9.3 Hz, 2H), 3.27-3.19 (m, 4H), 3.07 (dd, J

= 6.4, 9.3 Hz, 2H). ^{13}C NMR (100 MHz, DMSO-d_6) δ 144.1, 128.4, 127.8, 126.9, 93.2, 85.5, 73.5, 71.8, 71.1, 70.6, 63.5. HRMS (*m/z*): $[M-H]^-$ calcd for $C_{50}H_{49}O_{11}$, 825.3280; found, 825.3287. IR (neat) ν_{max}/cm^{-1} 3301, 2928, 1076, 1000, 990. $[\alpha]_D^{26.3}$= + 83.8 (c 0.50, MeOH).

2,3,4,2',3',4'-Hexa-(benzyloxy)-6,6'-di-(trityloxy)-α,α-trehalose

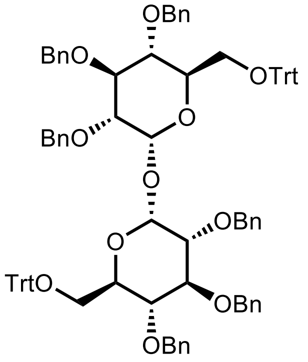

The ditritylated disaccharide (1.06 g, 1.28 mmol, 1.0 equiv.) was mixed with powdered KOH (7.8 g, 140 mmol, 110 equiv.) and dissolved in dioxane (10 mL) in a 100 mL round-bottom flask. The mixture was heated to reflux and benzyl chloride (9 mL, 80 mmol, 60 equiv.) was added. The reaction mixture was re-fluxed for approximately 2 h after which the solvent was distilled off and the crude mixture was allowed to cool to rt. Water was added and the mixture was left overnight. The product was extracted twice with diethyl ether. The organic phase was washed with water and dried over anhydrous Na_2SO_4. The ether was removed under reduced pressure. The crude mixture was used directly in the synthesis of the detritylated disaccharide 2,3,4,2',3',4',-hexa-benzyloxy-α,α-trehalose without further purification and only a crude ^1H NMR spectrum was obtained to confirm the formation of the hexabenzylated product.

2,3,4,2',3',4'-Hexa-(benzyloxy)-α,α-trehalose

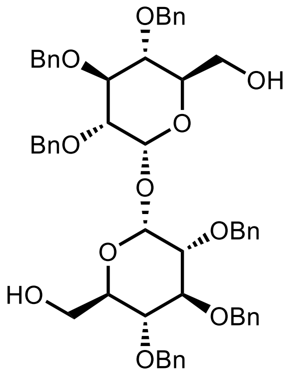

The crude hexabenzylated disaccharide (1.75 g, 1.28 mmol, 1.0 equiv.) was dissolved in excess acetic acid (80% in water, 35 mL, 490 mmol) and stirred at 75-80 °C. After 7.5 h the acetic acid was removed under reduced pressure. The crude mixture was purified by FC (SiO_2, 18x6 cm, EtOAc/Pentane 1:1 → 2:1) to give the detritylated product as an off-white foam (0.73 g, 0.82 mmol, 64%). R_f 0.37 (EtOAc/Pentane 1:1 (CAM-stain)). ^1H NMR (400 MHz, $CDCl_3$) δ 7.33-7.20 (m, 30H), 5.09 (d, *J* = 3.6 Hz, 2H), 4.96 (m, 2H), 4.86-4.82 (m, 4H), 4.69-4.59 (m, 6H), 4.05-4.00 (m, 4H), 3.55-3.47 (m, 8H). ^{13}C NMR (100 MHz, $CDCl_3$) δ 138.9, 138.3, 138.2, 128.6, 128.5, 128.5, 128.2, 128.0, 127.8, 127.7, 127.6, 94.1, 81.7, 79.6, 77.5, 75.7, 75.2, 73.2, 71.5, 61.7. HRMS (*m/z*): $[M-H]^-$ calcd for $C_{54}H_{57}O_{11}$, 881.3906; found, 881.3921. IR (neat) ν_{max}/cm^{-1} 3468, 3030, 2926, 2875, 1497, 1454, 1360, 1068, 1027, 989, 733, 695. $[\alpha]_D^{26.3}$= + 100.5 (c 0.50, $CHCl_3$).

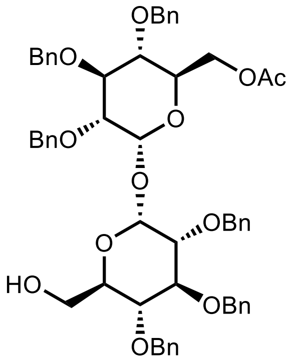

Furthermore, the monoacetylated product, 2,3,4,2',3',4',-hexa-benzyloxy-6-acetyloxy-α,α-trehalose, was recovered as a dark yellow oil (0.127 g, 0.137 mmol, 11 %). R_f 0.54 (EtOAc/Pentane 1:1 (CAM-stain)). ^{1}H NMR (400 MHz, $CDCl_3$) δ 7.37-7.27 (m, 30H), 5.15 (d, *J* = 3.7 Hz, 2H), 5.03-4.98 (m, 2H), 4.90-4.85 (m, 4H), 4.74-4.63 (m, 5H), 4.54 (d, *J* = 11.0 Hz, 1H), 4.25-4.21 (m, 1H), 4.14-4.03 (m, 5H), 3.59-3.50 (m, 6H), 1.97 (s, 3H). ^{13}C NMR (100 MHz, $CDCl_3$) δ 170.7, 138.8, 138.7, 138.3, 138.0, 138.0, 138.0, 128.5, 128.5, 128.5, 128.5, 128.2, 128.2, 128.0, 127.9, 127.8, 127.8, 127.7, 127.6, 127.6, 127.5, 94.1, 93.9, 81.7, 81.6, 79.4, 79.4, 77.4, 77.4, 75.7, 75.6, 75.1, 73.1, 73.0, 71.4, 69.2, 62.9, 61.5, 20.9. HRMS (*m/z*): $[M-H]^-$ calcd for $C_{56}H_{59}O_{12}$, 923.4012; found, 923.4032. IR (neat) ν_{max}/cm^{-1} 3030, 2928, 2875, 1741, 1497, 1453, 1361, 1236, 1068, 992, 733, 695. $[\alpha]_D^{26.3}$= + 89.8 (c 0.50, $CHCl_3$).

6,6'-Di-(trityloxy)-α,β-trehalose

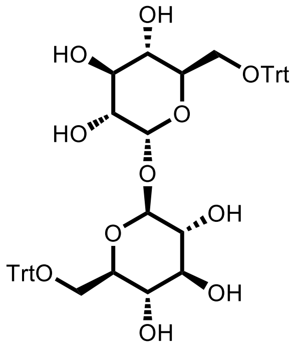

α,β-trehalose (0.247 g, 0.722 mmol, 1.0 equiv.) was added to a flame-dried flask with a magnetic stirring bar and dissolved in dry pyridine and concentrated under reduced pressure and subsequently placed under high vacuum for 2 h. The disscaharide was then redissolved in dry pyridine (3 mL) and TrtCl (0.503 g, 1.80 mmol, 2.5 equiv.) was added and the mixture was stirred under argon at rt for 24 h. After 24 h the mixture was heated to 40 °C and stirred for another 18 h before TrtCl (0.050 g, 0.18 mmol, 0.25 equiv.) was added. This mixture was stirred for 3 h, after which another portion of TrtCl (0.050 g, 0.18 mmol, 0.25 equiv.) was added and the reaction was stirred for 1 h before the flask was placed in the fridge overnight. The pyridine was evaporated under reduced pressure and the crude product was purified by FC (SiO_2, 2.5x12 cm, 5 % MeOH/$CHCl_3$ + 1 % TEA → 10 % MeOH/$CHCl_3$ + 1 % TEA). Purification gave the product as a white solid (0.440 g, 0.532 mmol, 74 %).

2,3,4,2',3',4'-Hexa-(benzyloxy)-α,β-trehalose

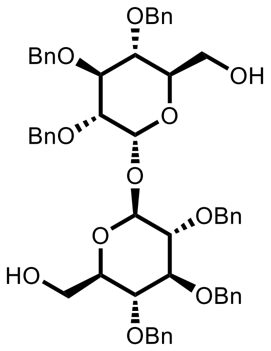

6,6'-di-trityloxy-α,β-trehalose (0.400 g, 0.484 mmol, 1.0 equiv.) and powdered KOH (3.2 g, 56.173 mmol, 116.1 equiv.) was dissolved in 1,4-dioxane (4 mL) and heated to reflux. BnCl (3.6 mL, 31.3 mmol, 64.6 equiv.) was added to the boiling mixture and this was stirred for 1 h. The crude mixture was concentrated *in vacuo*. The solids were redissolved in water and extracted with Et_2O (2 x 50 mL), the organic phases were washed with H_2O and dried over Na_2SO_4. The solvents were removed under reduced pressure to give an orange oily residue. The oil was redissolved in AcOH (10 mL, 80 %, aq.) and heated to 75 °C and stirred for 7 h. The acetic acid was removed *in vacuo*. The crude product was purified by FC (SiO_2, 2.5 x 11 cm, EtOAc/pentane 1:1 → EtOAc/pentane 3:1) to give the product as a white solid (0.174 g, 0.197 mmol, 41 %). R_f 0.14 (EtOAc/Pentane 1:1 (CAM-stain)). ^{1}H NMR (400 MHz, $CDCl_3$) δ 7.42 – 7.17 (m, 30H), 5.14 (d, J = 11.3 Hz, 1H), 5.06 (d, J = 3.5 Hz, 1H), 5.01 (d, J = 11.0 Hz, 1H), 4.94 (d, J = 10.9 Hz, 1H), 4.92 – 4.81 (m, 4H), 4.79 (d, J = 4.0 Hz, 1H), 4.77 – 4.56 (m, 6H), 4.22 – 4.06 (m, 2H), 3.85 (dd, J = 11.2, 2.6 Hz, 1H), 3.82 – 3.77 (m, 1H), 3.73 – 3.28 (m, 9H). ^{13}C NMR (100 MHz, $CDCl_3$) δ 138.7, 138.7, 138.6, 138.0, 138.0, 128.6, 128.6, 128.6, 128.5, 128.4, 128.2, 128.2, 128.1, 128.1, 128.0, 128.0, 127.9, 127.8, 127.7, 127.6, 105.0, 99.6, 84.5, 82.0, 81.8, 79.9, 78.9, 77.8, 76.1, 75.8, 75.8, 75.3, 75.2, 74.8, 73.4, 71.8, 63.1, 62.3. HRMS (*m/z*): [M-H]$^-$ calcd for $C_{54}H_{57}O_{11}$, 881.3906; found, 881.3978. IR (neat) ν_{max}/cm^{-1} 3484, 3032, 2899, 1497, 1453, 1358, 1094, 1016, 730, 694. $[\alpha]_D^{25.2}$= + 47.5 (c 0.5, $CHCl_3$).

2,3,4,2',3',4'-Hexa-(benzyloxy)-6,6'-Bis-(2,4-bis-(benzyloxy)-6-methylbenzoate)-α,α-trehalose

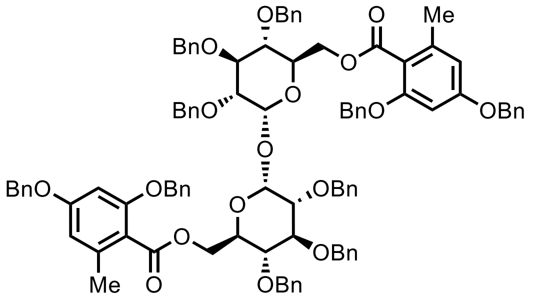

An azeotrope was formed with the benzyl-protected α,α-trehalose (50 mg, 0.06 mmol, 1.0 equiv.) and 2,4-bis-(benzyloxy)-6-methylbenzoic acid (43 mg, 0.12 mmol, 2.2 equiv.) in dry benzene and this was concentrated to dryness. This was repeated three times. The residue was then dissolved in dry DCM (0.24 mL) in a flame-dried schlenk flask containing a stirring bar and the flask was flushed with Ar. The mixture was cooled to 0°C and DCC (0.03 g, 0.14 mmol, 2.4 equiv.) and a catalytic amount of DMAP were added. The reaction was allowed to reach rt and was stirred overnight under an argon atmosphere. After 26 h the reaction mixture was filtered to remove the urea product of DCC. The filtrate was concentrated *in vacuo*. Purification of the crude product by FC (SiO_2, 15x2 cm, EtOAc/Pentane 1:5 → EtOAc/Pentane 1:4 → EtOAc/Pentane 1:2) yielded the diester (62 mg, 0.04 mmol, 71%) as a colorless oil. R_f

0.50 (EtOAc/Pentane 1:5 (CAM-stain)). ^{1}H NMR (400 MHz, $CDCl_3$) δ 7.39-7.14 (m, 50H), 6.36 (d, J = 1.8 Hz, 2H), 6.31 (d, J = 1.8 Hz, 2H), 5.10 (d, J = 3.5 Hz, 2H), 5.02-4.92 (m, 12H), 4.83-4.74 (m, 4H), 4.57-4.48 (m, 8H), 4.26 (d, J = 10.2 Hz, 4H), 4.00 (t, J = 9.6 Hz, 2H), 3.59 (t, J = 9.6 Hz, 2H), 3.43 (dd, J = 3.5, 9.6 Hz, 2H), 2.24 (s, 6H). ^{13}C NMR (100 MHz, $CDCl_3$) δ 168.1, 160.4, 157.2, 138.9, 138.4, 138.2, 138.0, 136.7, 136.6, 128.7, 128.6, 128.5, 128.4, 128.4, 128.2, 128.2, 127.9, 127.8, 127.7, 127.6, 127.5, 126.8, 117.2, 108.2, 98.5, 93.7, 81.5, 79.2, 77.9, 75.5, 75.3, 72.6, 70.1, 69.4, 63.2, 20.1. IR (neat) ν_{max}/cm^{-1} 3031, 2926, 2864, 1725, 1602, 1586, 1497, 1453, 1263, 1157, 1089, 1070, 995, 733, 698. $[\alpha]_D^{26.8}$ = + 55.2 (c 0.50, $CHCl_3$).

2,3,4,2′,3′,4′-Hexa-(benzyloxy)-6-acetyloxy-6′-(2,4-bis-(benzyloxy)-6-methylbenzoate)-α,α-trehalose

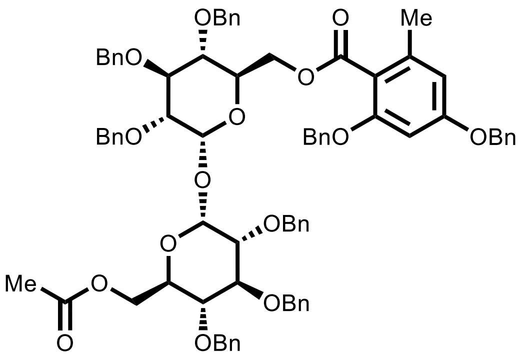

To a flame-dried flask were added 2,3,4,2′,3′,4′,-hexa-benzyloxy-6-acetyloxy-α,α-trehalose (0.065 g, 0.07 mmol, 1.0 equiv.) and 2,4-bis-(benzyloxy)-6-methylbenzoic acid (0.049 g, 0.14 mmol, 2.0 equiv.) and the compounds were dissolved in dry benzene to form an azeotrope with potential residual water. The mixture was concentrated to dryness and placed under high vacuum.. This step was repeated with dry toluene. Subsequently, the compounds were redissolved in dry toluene (2.5 mL) and PPh_3 (0.037 g, 0.141 mmol, 2.0 equiv.) was added after which the mixture was cooled to 0 °C. Finally, DIAD (27 µL, 0.141 mol, 2.0 equiv.) was added and the reaction mixture was stirred for 2 h. The reaction was quenched with ice-water and extracted twice with EtOAc. The organic phase was washed with brine, dried over Na_2SO_4 and concentrated *in vacuo*. The crude yellow oil was purified by FC (SiO_2, 2.5 x 12 cm, 100 % DCM → 2.5 % Et_2O/DCM) to give the product as a viscous clear oil (0.050 g, 0.040 mmol, 58 %). R_f 0.71 (EtOAc/Heptane 1:1 (CAM-stain)). ^{1}H NMR (400 MHz, $CDCl_3$) δ 7.43 – 7.16 (m, 40H), 6.37 (d, J = 2.1 Hz, 1H), 6.33 (d, J = 2.1 Hz, 1H), 5.17 (d, J = 3.5 Hz, 1H), 5.12 (d, J = 3.5 Hz, 1H), 5.05 – 4.92 (m, 6H), 4.90 – 4.77 (m, 4H), 4.69 – 4.48 (m, 7H), 4.32 – 4.27 (m, 2H), 4.24 – 4.16 (m, 1H), 4.13 – 3.97 (m, 4H), 3.63 (t, J = 9.6 Hz, 1H), 3.57 – 3.44 (m, 3H), 2.27 (s, 3H), 1.97 (s, 3H). ^{13}C NMR (100 MHz, $CDCl_3$) δ 170.7, 168.1, 160.5, 157.2, 138.9, 138.8, 138.5, 138.1, 138.1, 138.0, 137.9, 136.8, 136.7, 128.8, 128.6, 128.6, 128.5, 128.5, 128.5, 128.3, 128.3, 128.2, 128.1, 128.0, 127.9, 127.8, 127.8, 127.7, 127.7, 127.6, 127.5, 126.8, 117.2, 108.3, 98.5, 94.1, 94.0, 81.7, 81.6, 79.5, 79.1, 78.0, 77.4, 75.8, 75.6, 75.4, 75.2, 73.0, 72.7, 70.2, 70.2, 69.6, 69.1, 63.3, 63.0, 21.0, 20.2. HRMS (m/z): $[M+Na]^+$ calcd for $C_{78}H_{78}NaO_{15}$, 1277.5233; found, 1277.5242. IR (neat) ν_{max}/cm^{-1} 3063, 3031, 2925, 1737, 1603, 1497, 1454, 1365, 1326, 1251, 1158, 1091, 1070, 1027, 996, 909, 828, 732, 696, 644. $[\alpha]_D^{29.8}$+ 73.0 (c 1.00, $CHCl_3$).

2,3,4,2′,3′,4′-Hexa-(benzyloxy)-6′-(2,4-bis-(benzyloxy)-6-methylbenzoate)-α,α-trehalose

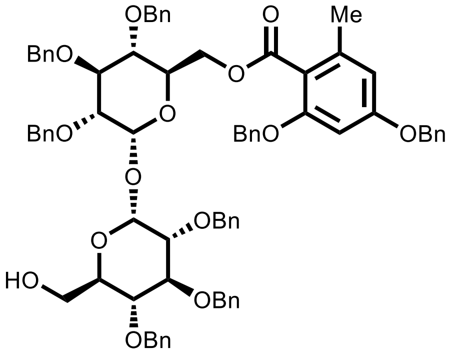

2,3,4,2′,3′,4′,-hexa-benzyloxy-6-acetyloxy-6′-(2,4-bis-(benzyloxy)-6-methylbenzoate)-α,α-trehalose (0.050 g, 0.040 mmol, 1.0 equiv.) was dissolved in benzene to form an azeotrope with potential residual water. The mixture was concentrated to dryness and placed under high vacuum. The compound was redissolved in dry MeOH/THF (1.0 mL, 6:4) under argon. Subsequently, solid K_2CO_3 (0.009 g, 0.065 mmol, 1.6 equiv.) was added and the stirring was continued at rt for 3 h. The crude product was concentrated under reduced pressure and purified by FC (SiO_2, 6 x 1 cm, 10 % Et_2O/DCM) to yield the product quantitatively as a yellow oil (0.048 g, 0.040 mmol). R_f 0.12 (EtOAc/Heptane 1:2 (CAM-stain)). ^{1}H NMR (400 MHz, $CDCl_3$) δ 7.44 – 7.20 (m, 40H), 6.41 (d, J = 2.1 Hz, 1H), 6.37 (d, J = 2.1 Hz, 1H), 5.18 (d, J = 3.5 Hz, 1H), 5.11 (d, J = 3.6 Hz, 1H), 5.06 – 4.96 (m, 6H), 4.92 – 4.85 (m, 3H), 4.82 (d, J = 10.4 Hz, 1H), 4.70 – 4.53 (m, 8H), 4.39 – 4.26 (m, 2H), 4.14 – 4.01 (m, 3H), 3.66 (t, J = 9.6 Hz, 1H), 3.61 – 3.48 (m, 5H), 2.30 (s, 3H). ^{13}C NMR (100 MHz, $CDCl_3$) δ 168.1, 160.5, 157.2, 138.9, 138.5, 138.4, 138.2, 138.1, 138.0, 136.8, 136.6, 128.8, 128.6, 128.6, 128.5, 128.5, 128.5, 128.4, 128.3, 128.2, 128.2, 128.0, 127.9, 127.8, 127.8, 127.7, 127.6, 127.6, 127.6, 127.6, 126.7, 117.2, 108.3, 98.6, 93.9, 93.9, 81.7, 81.6, 79.6, 79.3, 78.0, 77.5, 77.2, 76.8, 75.7, 75.6, 75.3, 75.1, 73.0, 72.8, 71.3, 70.2, 70.2, 69.6, 63.3, 61.7, 20.2. HRMS (*m/z*): $[M-H]^-$ calcd for $C_{76}H_{75}O_{14}$, 1211.5162; found, 1211.5173. IR (neat) ν_{max}/cm^{-1} 3031, 2926, 1725, 1603, 1497, 1453, 1262, 1157, 1069, 994, 733, 695. $[\alpha]_D^{24.9}$+ 79.5 (c 0.50, $CHCl_3$).

2,3,4,2′,3′,4′-Hexa-(benzyloxy)-6,6′-bis-benzoate-α,α-trehalose

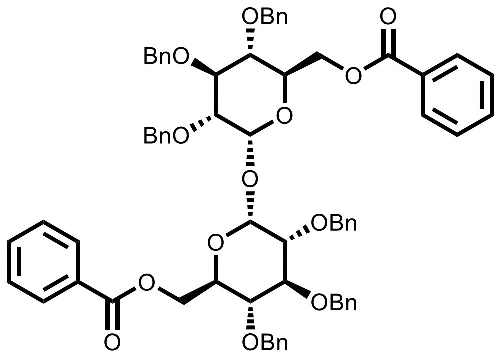

The benzyl-protected trehalose (0.089 g, 0.1 mmol, 1.0 equiv.) and benzoic acid (0.030 g, 0.218 mmol, 2.18 equiv.) were dissolved in dry toluene to form an azeotrope with potential residual water. The mixture was concentrated to dryness and placed under high vacuum. This step was repeated three times. The mixture was then dissolved in dry DCM (0.50 mL) in a flame-dried schlenk flask with a stirring bar and flushed with Ar. The mixture was cooled to 0°C and added DCC (0.050 g, 0.24 mmol, 2.4 equiv.) and a catalytic amount of DMAP. The reaction was allowed to warm to rt and stirred overnight under argon. The reaction was stopped after 22 h and the urea product of DCC was filtered off. The filtrate was concentrated. The crude

product was purified by FC (SiO_2, 2x15 cm, Heptane 100% (150 mL) → EtOAc/heptane 1:4 (250 mL) → EtOAc/heptane 1:2 (250 mL). The pure product was isolated as a colorless oil (77 mg, 0.071 mmol, 71%). R_f 0.70 (EtOAc/Heptane 1:1 (CAM-stain)). ^{1}H NMR (400 MHz, $CDCl_3$) δ 7.96 (dd, J = 8.4, 1.3 Hz, 4H), 7.58 – 7.50 (m, 2H), 7.45 – 7.20 (m, 34H), 5.23 (d, J = 3.6 Hz, 2H), 5.04 (d, J = 10.6 Hz, 2H), 4.94 – 4.86 (m, 4H), 4.79 – 4.66 (m, 4H), 4.58 (d, J = 10.7 Hz, 2H), 4.37 – 4.30 (m, 3H), 4.27 (dd, J = 12.6, 3.9 Hz, 2H), 4.12 (t, J = 9.3 Hz, 2H), 3.73 – 3.65 (m, 2H), 3.62 (dd, J = 9.6, 3.6 Hz, 2H). ^{13}C NMR (100 MHz, $CDCl_3$) δ 166.2, 138.6, 137.9, 137.9, 133.2, 130.0, 129.7, 128.6, 128.6, 128.6, 128.5, 128.3, 128.2, 128.1, 127.9, 127.9, 127.5, 94.1, 81.9, 79.6, 77.8, 76.0, 75.4, 73.1, 69.4, 63.1. HRMS (*m/z*): $[M+H]^+$ calcd for $C_{68}H_{67}O_{13}$, 1091.4576; found, 1091.4482. IR (neat) ν_{max}/cm^{-1} 3031, 2907, 1719, 1601, 1496, 1452, 1270, 1094, 1067, 992, 695. $[\alpha]_D^{25.3}$+ 138.2 (c 1.00, $CHCl_3$).

2,3,4,2',3',4'-Hexa-(benzyloxy)-6,6'-bis-(2,3-methoxybenzoate)-α,α-trehalose

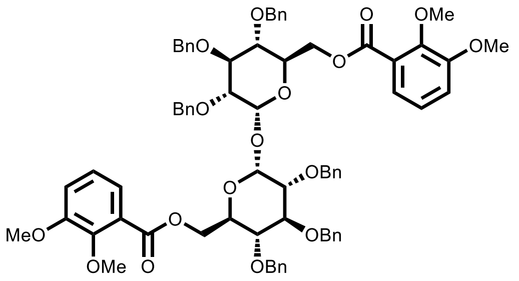

Hexabenzylated trehalose (0.0507 g, 0.057 mmol, 1.0 equiv.) and 2,3-dimetoxybenzoic acid (0.023 g, 0.127 mmol, 2.22 equiv.) was suspended in dry benzene and concentrated to dryness. Subsequently, the compounds were redissolved in dry DCM (0.36 mL) in a flame-dried schlenk flask with a magnetic stirring bar and the flask was flushed with Ar. The mixture was cooled to 0°C and DCC (0.028 g, 0.138 mmol, 2.4 equiv.) and a catalytic amount of DMAP were added. The reaction was allowed to warm to rt and stirred overnight under argon. The crude product was purified by dry column chromatography (SiO_2, 2x5.5 cm, EtOAc/heptane 0:1 → 1:1) to give the desired product (0.0569 g, 0.047 mmol, 82 %). R_f 0.55 (EtOAc/Heptane 1:1 (CAM-stain)). ^{1}H NMR (400 MHz, $CDCl_3$) δ 7.42 – 7.20 (m, 30H), 7.06 – 7.03 (m, 6H), 5.20 (d, J = 3.6 Hz, 2H), 5.02 (d, J = 10.8 Hz, 2H), 4.88 (dd, J = 10.7, 4.9 Hz, 4H), 4.73 – 4.64 (m, 4H), 4.58 (d, J = 10.6 Hz, 2H), 4.39 (dd, J = 12.2, 3.3 Hz, 2H), 4.35 – 4.22 (m, 4H), 4.08 (t, J = 9.3 Hz, 2H), 3.88 (s, 6H), 3.85 (s, 6H), 3.69 (t, J = 10.2, 9.1 Hz, 2H), 3.57 (dd, J = 9.7, 3.5 Hz, 2H). ^{13}C NMR (100 MHz, $CDCl_3$) δ 166.0, 153.6, 149.3, 138.8, 138.1, 137.9, 128.6, 128.6, 128.5, 128.3, 128.1, 128.0, 127.9, 127.7, 127.6, 125.9, 123.9, 122.5, 116.0, 94.1, 81.8, 79.4, 77.8, 75.8, 75.5, 73.1, 69.4, 63.3, 61.7, 56.2. HRMS (*m/z*): $[M+H]^+$ calcd for $C_{72}H_{75}O_{17}$, 1211.4999; found, 1211.5026. IR (neat) ν_{max}/cm^{-1} 3030, 2935, 1729, 1582, 1497, 1479, 1454, 1426, 1359, 1310, 1262, 1195, 1151, 1055, 997, 910, 802, 733, 697, 644. $[\alpha]_D^{29.6}$+ 76.7 (c 0.50, $CHCl_3$).

2,3,4,2′,3′,4′-Hexa-(benzyloxy)-6,6′-bis-(2,4-bis-(benzyloxy)-6-methylbenzoate)-α,β-trehalose

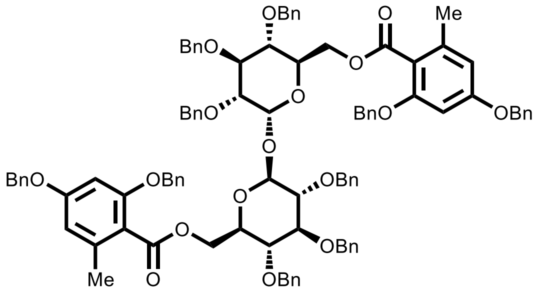

The hexabenzylated α,β-trehalose (0.050 g, 56.7 µmol, 1.0 equiv.) was added to a flame-dried flask and an azeotrope was formed with dry benzene, concentrated to dryness and subsequently placed under high vacuum. This step was repeated once. The flask was purged with argon and the compound was redissolved in dry THF/toluene (2 mL, 1:1) and to this was added 2,4-bis-(benzyloxy)-6-methylbenzoic acid (0.059 g, 170 µmol, 3.0 equiv.) and PPh_3 (0.045 g, 170 µmol, 3.0 equiv.). The mixture was cooled to 0 °C in an ice bath and subsequently DIAD (33 µL, 170 µmol, 3.0 equiv.) was added. The reaction was stirred at 0 °C for 2.5 h until TLC showed full conversion of starting material. The reaction mixture was diluted with ice-water and extracted with EtOAc (3x), after which the organic phases were washed with brine, dried over Na_2SO_4 and concentrated *in vacuo*. The crude product was purified by FC (SiO_2, 2 x 11 cm, EtOAc/Pentane 10% → 20 % → 25 % → 30 %). The product was isolated as a colorless oil (0.043 g, 27 µmol, 48 %). R_f 0.71 (EtOAc/Heptane 1:1 (CAM-stain)). ^1H NMR (400 MHz, $CDCl_3$) δ 7.41 – 7.08 (m, 50H), 6.36 (d, J = 2.1 Hz, 1H), 6.34 (d, J = 2.2 Hz, 1H), 6.29 (d, J = 2.1 Hz, 2H), 5.11 – 5.02 (m, 4H), 5.02 – 4.98 (m, 1H), 4.98 – 4.91 (m, 5H), 4.87 (t, J = 10.8 Hz, 2H), 4.79 – 4.67 (m, 6H), 4.59 – 4.44 (m, 6H), 4.39 (dd, J = 12.0, 3.9 Hz, 1H), 4.29 – 4.18 (m, 2H), 3.96 (t, J = 9.3 Hz, 1H), 3.66 (t, J = 9.5 Hz, 1H), 3.62 – 3.56 (m, 2H), 3.53 – 3.47 (m, 1H), 3.46 – 3.35 (m, 2H), 2.23 (s, 3H), 2.20 (s, 3H). ^{13}C NMR (100 MHz, $CDCl_3$) δ 167.9, 167.8, 160.3, 160.3, 157.0, 157.0, 138.7, 138.5, 138.5, 138.4, 138.3, 137.9, 137.8, 136.8, 136.7, 136.6, 136.5, 128.6, 128.6, 128.5, 128.5, 128.4, 128.4, 128.3, 128.2, 128.1, 128.0, 128.0, 127.8, 127.7, 127.7, 127.6, 127.6, 127.5, 127.5, 127.5, 127.4, 127.3, 126.7, 126.6, 117.1, 116.8, 108.0, 107.9, 103.7, 99.1, 98.4, 98.3, 84.3, 81.6, 81.5, 79.6, 77.5, 75.6, 75.3, 75.1, 75.0, 74.4, 73.3, 73.0, 70.1, 70.0, 70.0, 69.9, 20.1, 20.0. HRMS (*m/z*): $[M+H]^+$ calcd for $C_{98}H_{95}O_{17}$, 1543.6564; found, 1543.6603. IR (neat) ν_{max}/cm^{-1} 3031, 2924, 1725, 1602, 1497, 1453, 1325, 1263, 1158, 1072, 1043, 1027, 909, 732, 695.

6,6′-Bis-(2,4-dihydroxy-6-methylbenzoate)-α,α-trehalose (1)

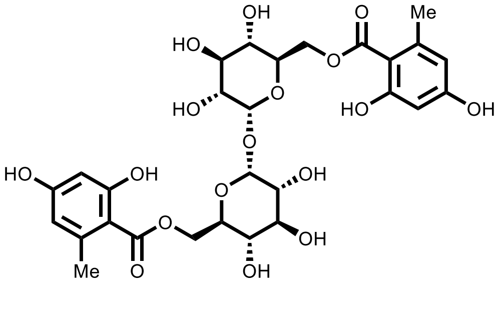

The benzyl protected brartemicin (33.1 mg, 21.4 µmol, 1.0 equiv.) was dissolved in MeOH/$CHCl_3$ 1:1 (5 mL) in a 50 mL round-bottom flask and the flask was then purged with argon. To the solution was added $Pd(OH)_2/C$ (23 mg, 20 %, 32 µmol,

1.5 equiv.) and the mixture was stirred while flushed with argon. The atmosphere in the flask was subsequently saturated with H_2-gas from a balloon. After 6 h the reaction was completed as judged by TLC and the mixture was filtered to remove residues of the catalyst. The solvents were removed *in vacuo* to yield brartemicin (**1**) as an analytically pure off-white solid (13.5 mg, 21.0 µmol, 98%). The product was purified further by semi-preparative C-18 RP HPLC prior to binding assays (5% → 70% MeOH in H_2O over 17 min., hold 3 min., then 70 % → 100 % MeOH, hold 1 min., 10 mL/min., RT = 16.7 min., Phenomenex Luna 5u C18(2) 100 A, New Column, 250 x 10 mm). R_f 0.58 (EtOAc/MeOH/H_2O 4:1:1 (CAM-stain)). ^{1}H NMR (400 MHz, CD_3OD) δ 6.21 (d, *J* = 2.3 Hz, 2H), 6.15 (d, *J* = 2.3 Hz, 2H), 5.13 (d, *J* = 3.7 Hz, 2H), 4.58 (dd, *J* = 2.0, 12.0 Hz, 2H), 4.46 (dd, *J* = 4.9, 12.0 Hz, 2H), 4.21-4.17 (m, 2H), 3.83 (t, *J* = 9.6 Hz, 2H), 3.49 (dd, *J* = 3.7, 9.6 Hz, 2H), 3.43 (t, *J* = 9.6 Hz, 2H) 2.51 (s, 6H). ^{13}C NMR (100 MHz, CD_3OD) δ 172.8, 166.3, 163.9, 144.9, 112.5, 105.6, 101.7, 95.6, 74.5, 73.2, 72.2, 71.3, 65.4, 24.9. HRMS (*m/z*): [M-H]$^-$ calcd for $C_{28}H_{33}O_{17}$, 641.1723; found, 641.1723. IR (neat) ν_{max}/cm^{-1} 3292, 2928, 2856, 1644, 1617, 1447, 1312, 1256. $[\alpha]_D^{26.5}$ = + 78.8 (c 0.50, MeOH).

6,6'-Bis-(2,3-dimethoxybenzoate)-α,α-trehalose (2)

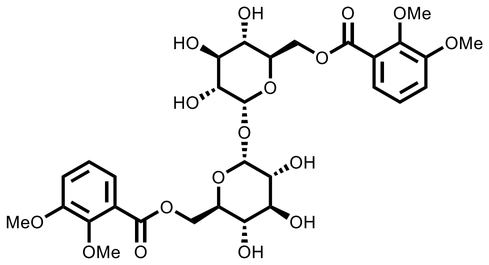

2,3,4,2',3',4'-Hexa-(benzyloxy)-6,6'-bis-(2,3-methoxybenzoate)-α,α-trehalose (0.0558 g, 0.0461 mmol, 1 equiv.) was dissolved in EtOH/EtOAc 1:1 (10 mL) in a 50 mL round-bottom flask and the flask was flushed with argon. To this was added $Pd(OH)_2$/C (0.021 g, 20 %, 0.03 mmol, 0.65 equiv.) while still flushing with argon. The atmosphere in the flask was then exchanged to H_2-gas from a balloon. After 8 h the reaction mixture was filtered and concentrated *in vacuo*. The reaction yielded the product as an analytically pure off-white solid (0.030 g, 0.045 mmol, 97 %). The product was purified by semi-preparative HPLC before binding assays (5 % → 65 % MeCN in H_2O over 20 min., hold for 5 min., then 65 % → 100 % MeCN, hold for 2 min., 10 mL/min., RT = 10.1 min., Phenomenex Luna 5u C18(2) 100 A, New Column, 250 x 10 mm). R_f 0.55 (EtOAc/MeOH/H_2O 4:1:1 (CAM-stain)). ^{1}H NMR (400 MHz, CD_3OD) δ 7.31 (dd, J = 7.9, 1.6 Hz, 2H), 7.21 (dd, J = 8.3, 1.6 Hz, 2H), 7.12 (t, J = 8.0 Hz, 2H), 5.12 (d, J = 3.8 Hz, 2H), 4.55 (dd, J = 12.0, 2.3 Hz, 2H), 4.45 (dd, J = 11.9, 5.0 Hz, 2H), 4.14 (ddd, J = 10.2, 5.1, 2.1 Hz, 2H), 3.88 (s, 6H), 3.84 (s, 6H), 3.81 (t, J = 9.3 Hz, 2H), 3.50 (dd, J = 9.7, 3.7 Hz, 2H), 3.45 (t, J = 9.4 Hz, 2H). ^{13}C NMR (100 MHz, $CDCl_3$) δ 167.9, 155.0, 150.2, 127.2, 125.2, 123.2, 117.4, 95.3, 74.6, 73.2, 72.0, 71.5, 65.2, 62.1, 56.6. HRMS (*m/z*): [M-H]$^-$ calcd for $C_{30}H_{37}O_{17}$, 669.2036; found, 669.2044. IR (neat) ν_{max}/cm^{-1} 3368, 2934, 1711, 1625, 1581, 1480, 1428, 1311, 1263, 1149, 1046, 1022, 987, 940, 805, 753, 640. $[\alpha]_D^{26.7}$+ 93.6 (c 0.25, MeOH).

6-(2,4-Dihydroxy-6-methylbenzoate)-α,α-trehalose (4)

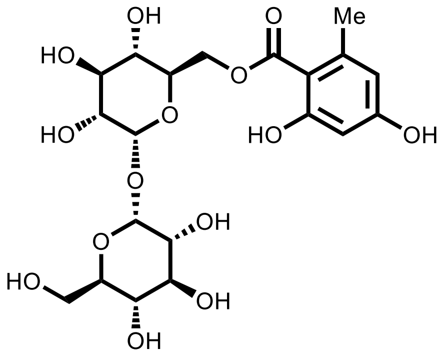

2,3,4,2′,3′,4′,-hexa-benzyloxy-6′-(2,4-bis-(benzyloxy)-6-methylbenzoate)-α,α-trehalose (0.048 g, 0.040 mmol, 1.0 equiv.) was dissolved in MeOH/$CHCl_3$ (5 mL, 1:1) and the flask was purged with argon. $Pd(OH)_2$/C (0.020 g, 20 %, 0.7 equiv.) was added, the atmosphere changed to H_2-gas and the mixture was stirred at rt for 3.5 h. The mixture was filtered through celite with MeOH/$CHCl_3$ (2:1) and concentrated under reduced pressure. This yielded the product quantitatively as an analytically pure solid. The product was purified on HPLC before binding assays (5 % → 65 % MeCN in water over 20 min., hold for 1 min., then 65 % → 100 % MeCN, hold for 1 min., 10 mL/min., RT = 3.0 min., Phenomenex Luna 5u CN 100 A, 250 x 10 mm, 5 micron). R_f 0.38 (EtOAc/MeOH/H_2O 4:1:1 (CAM-stain)). ^{1}H NMR (400 MHz, CD_3OD) δ 6.20 (dd, J = 2.3, 0.7 Hz, 1H), 6.15 (dd, J = 2.4, 0.6 Hz, 1H), 5.13 (dd, J = 12.0, 3.7 Hz, 2H), 4.58 (dd, J = 12.0, 2.3 Hz, 1H), 4.46 (dd, J = 12.0, 5.1 Hz, 1H), 4.17 (ddd, J = 10.2, 5.0, 2.3 Hz, 1H), 3.89 – 3.74 (m, 4H), 3.67 (dd, J = 11.8, 5.4 Hz, 1H), 3.54 – 3.38 (m, 3H), 2.51 (d, J = 0.7 Hz, 3H). ^{13}C NMR (100 MHz, CD_3OD) δ 172.8, 166.4, 164.4, 144.8, 112.7, 105.4, 101.8, 95.3, 95.2, 74.7, 74.4, 73.9, 73.2, 72.3, 71.9, 71.3, 65.4, 62.6, 49.6, 49.4, 49.2, 49.0, 48.8, 48.6, 48.4, 24.9. HRMS (*m/z*): $[M+Na]^+$ calcd for $C_{20}H_{29}NaO_{14}$, 515.1377; found, 515.1375. IR (neat) ν_{max}/cm^{-1} 3288, 2933, 1645, 1446, 1312, 1258, 1203, 1149, 1100, 984, 800, 575. $[\alpha]_D^{26.0}$ = + 119.8 (c 1.00, MeOH).

6,6′-Bis-(benzoate)-α,α-trehalose (3)

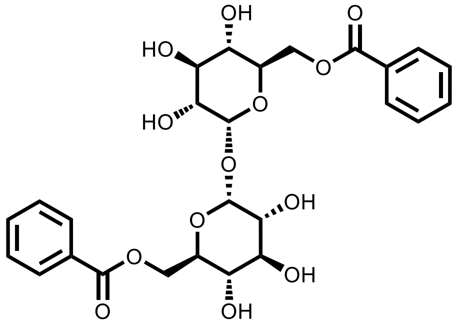

2,3,4,2′,3′,4′-Hexa-(benzyloxy)-6,6′-bis-benzoate-α,α-trehalose (0.029 g, 0.0266 mmol, 1.0 equiv.) was dissolved in MeOH/$CHCl_3$ 1:1 (5 mL) in a 10 mL round-bottom flask and the flask was purged with argon. $Pd(OH)_2$/C (0.0273 g, 20 %, 0.0358 mmol, 1.35 equiv.) was added and the mixture was stirred to suspend the reagent. The atmosphere in the flask was then exchanged with H_2-gas from a balloon. After stirring at rt for 22 h the reaction had reached full conversion as judged by TLC. The reaction mixture was filtered through a syringe filter (PTFE) and the solvents were removed *in vacuo* to yield the desired product as an analytically pure off-white solid (15.3 mg, 27.8 µmol, quant.). The product was purified by semi-preparative HPLC prior to binding assays (5 % → 65 %

MeCN in H_2O over 20 min., hold for 1 min., then 65 % → 100 % MeCN, hold for 1 min., 10 mL/min., RT = 8.9 min., Phenomenex Luna 5u CN 100 A, 250 x 10 mm, 5 micron). R_f 0.57 (EtOAc/MeOH/MeCN/H_2O 6:1:1:1 (CAM-stain)). ^{1}H NMR (400 MHz, CD_3OD) δ 8.04 (d, *J* = 7.9 Hz, 4H), 7.59 (t, *J* = 7.4 Hz, 2H), 7.47 (t, *J* = 7.7 Hz, 4H), 5.12 (d, *J* = 3.8 Hz, 2H), 4.58 (dd, *J* = 11.9, 2.2 Hz, 2H), 4.46 (dd, *J* = 11.9, 5.1 Hz, 2H), 4.20 (ddd, *J* = 10.2, 5.1, 2.1 Hz, 2H), 3.84 (t, *J* = 9.3 Hz, 2H), 3.53 (dd, *J* = 9.7, 3.8 Hz, 2H), 3.48 (dd, *J* = 10.1, 8.8 Hz, 2H). ^{13}C NMR (100 MHz, CD_3OD) δ 167.9, 134.3, 131.4, 130.6, 129.6, 95.5, 74.6, 73.2, 71.9, 71.6, 65.0. HRMS (m/z): $[M-H]^-$ calcd for $C_{26}H_{29}O_{13}$, 549.1614; found, 549.1635. IR (neat) ν_{max}/cm^{-1} 3344, 2931, 1699, 1451, 1316, 1275, 1071, 1024, 985, 708. $[\alpha]_D^{27.4} = +96.8$ (c 0.50, MeOH).

6,6'-Bis-(2,4-dihydroxy-6-methylbenzoate)-α,β-trehalose (5)

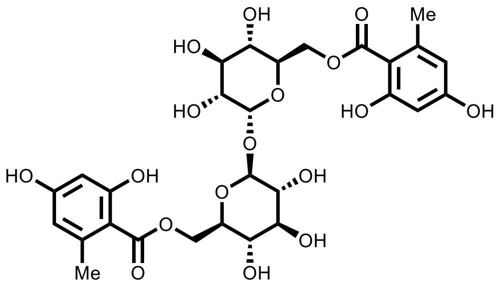

The globally benzylated α,β-brartemicin (0.042 g, 27 µmol, 1.0 equiv.) was dissolved in $CHCl_3$/MeOH (1:1, 5 mL) and the flask was purged with argon before $Pd(OH)_2$/C (0.020 g, 20 %, 28 µmol, 1.05 equiv.) was added. The flushing with argon was continued until the atmosphere in the flask was exchanged for H_2-gas. The reaction mixture was stirred under the H_2-atmosphere for 4 h at which time TLC showed full conversion of the starting material. The mixture was filtered over celite followed by washing of the celite with MeOH. The solvents were then removed *in vacuo*. This yielded the product quantitatively as a brownish solid with minor impurities and solvent traces. The product was subsequently purified by semi-preparative HPLC before binding assays (5 % → 65 % MeCN in water over 20 min., hold for 1 min., then 65 % → 100 % MeCN, hold for 2 min., RT = 5.5 min., 10 mL/min., Phenomenex Luna 5u CN 100 A, 250 x 10 mm 5 micron). R_f 0.58 (EtOAc/MeOH/H_2O 4:1:1 (CAM-stain)). ^{1}H NMR (400 MHz, CD_3OD) δ 6.19 – 6.03 (m, 4H), 5.12 (d, J = 3.7 Hz, 1H), 4.61 – 4.49 (m, 2H), 4.48 – 4.35 (m, 2H), 4.28 (dd, J = 11.9, 2.6 Hz, 1H), 4.06 (dt, J = 10.2, 2.9 Hz, 1H), 3.72 – 3.57 (m, 2H), 3.50 – 3.35 (m, 5H), 2.48 (s, 3H), 2.43 (s, 3H). ^{13}C NMR (100 MHz, CD_3OD) δ 172.7, 172.3, 166.3, 166.1, 163.9, 163.7, 144.7, 112.6, 112.5, 105.9, 102.8, 101.7, 77.4, 75.5, 75.3, 74.9, 73.8, 71.6, 71.5, 65.2, 64.4, 24.9, 24.8. HRMS (m/z): $[M-H]^-$ calcd for $C_{28}H_{33}O_{17}$, 641.1723; found, 641.1725. IR (neat) ν_{max}/cm^{-1} 3391, 2920, 2851, 1645, 1456, 1315, 1263, 1167, 1080.

References

1 Feinberg, H., Jégouzo, S. a F., Rowntree, T. J. W., Guan, Y., Brash, M. a, Taylor, M. E., Weis, W. I., Drickamer, K., *J. Biol. Chem.*, 2013, **288**, 28457.

2 Jégouzo, S., Harding, E., Acton, O., Rex, M., Fadden, A., Taylor, M., Drickamer, K., *Glycobiology*, 2014, In press.

Spectra

2,4-Dihydroxy-6-methylbenzaldehyde

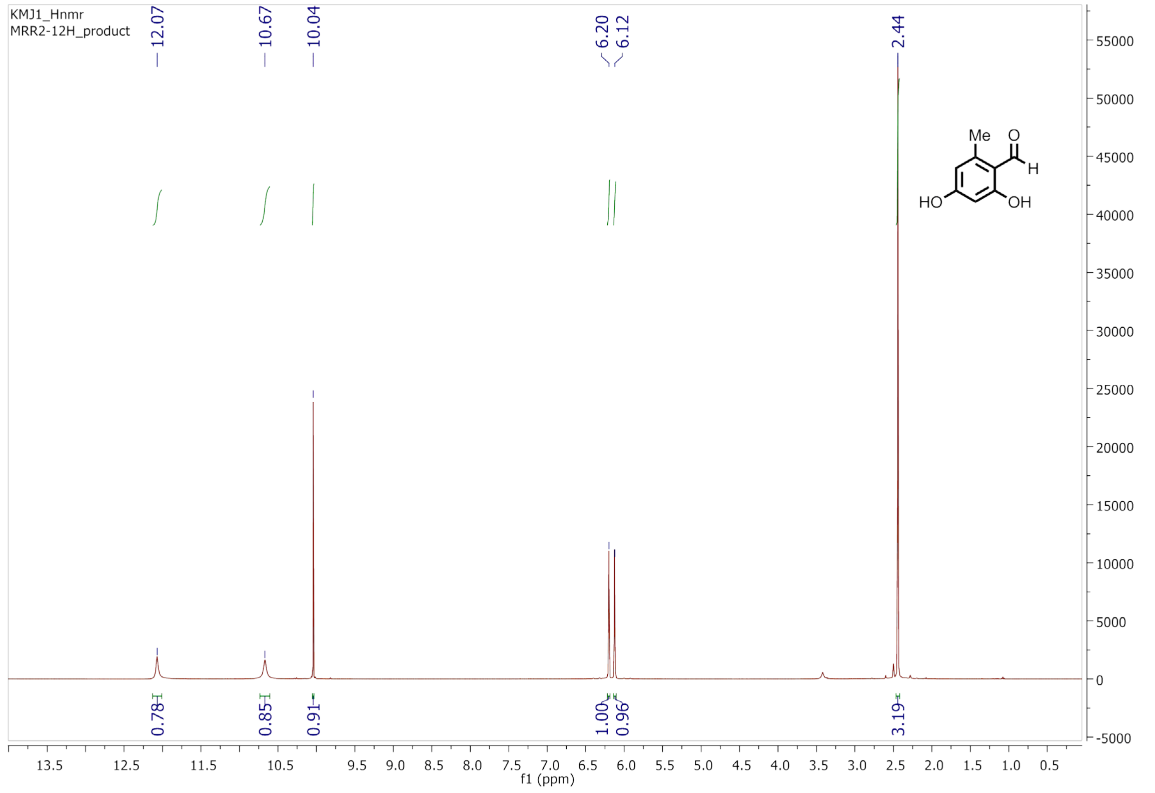

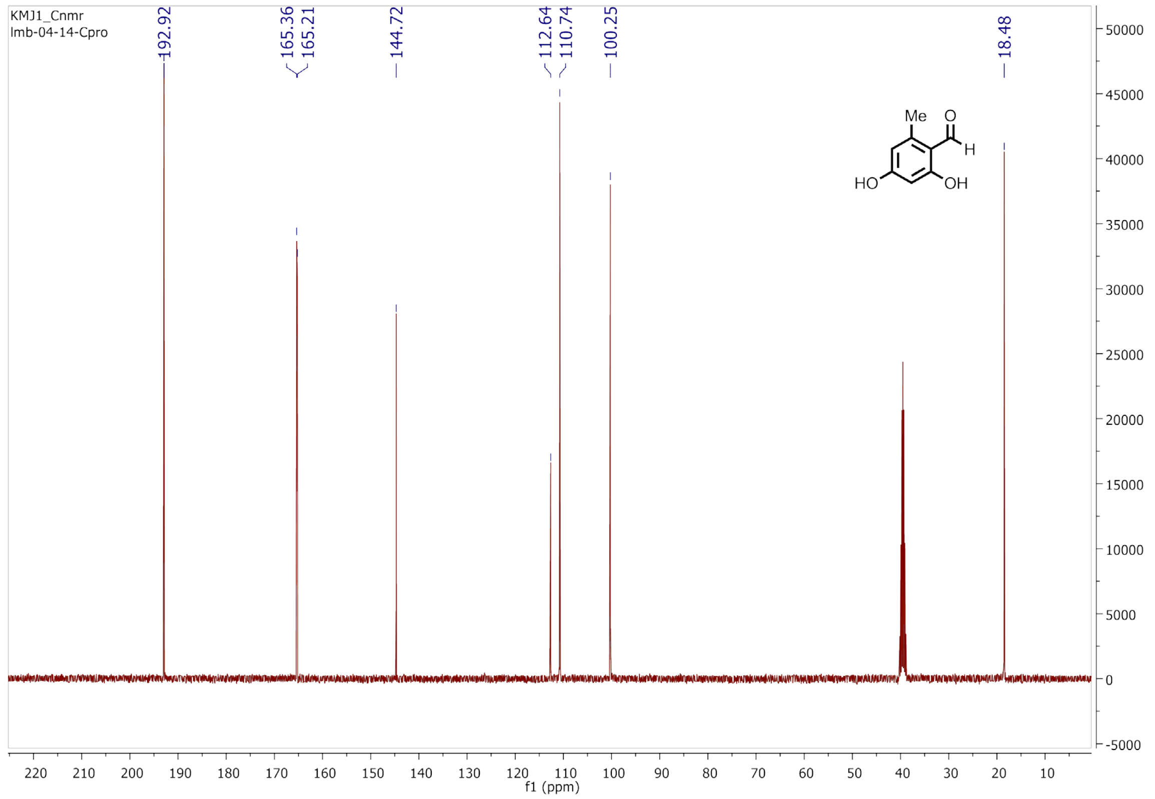

2,4-Bis-(benzyloxy)-6-methylbenzaldehyde

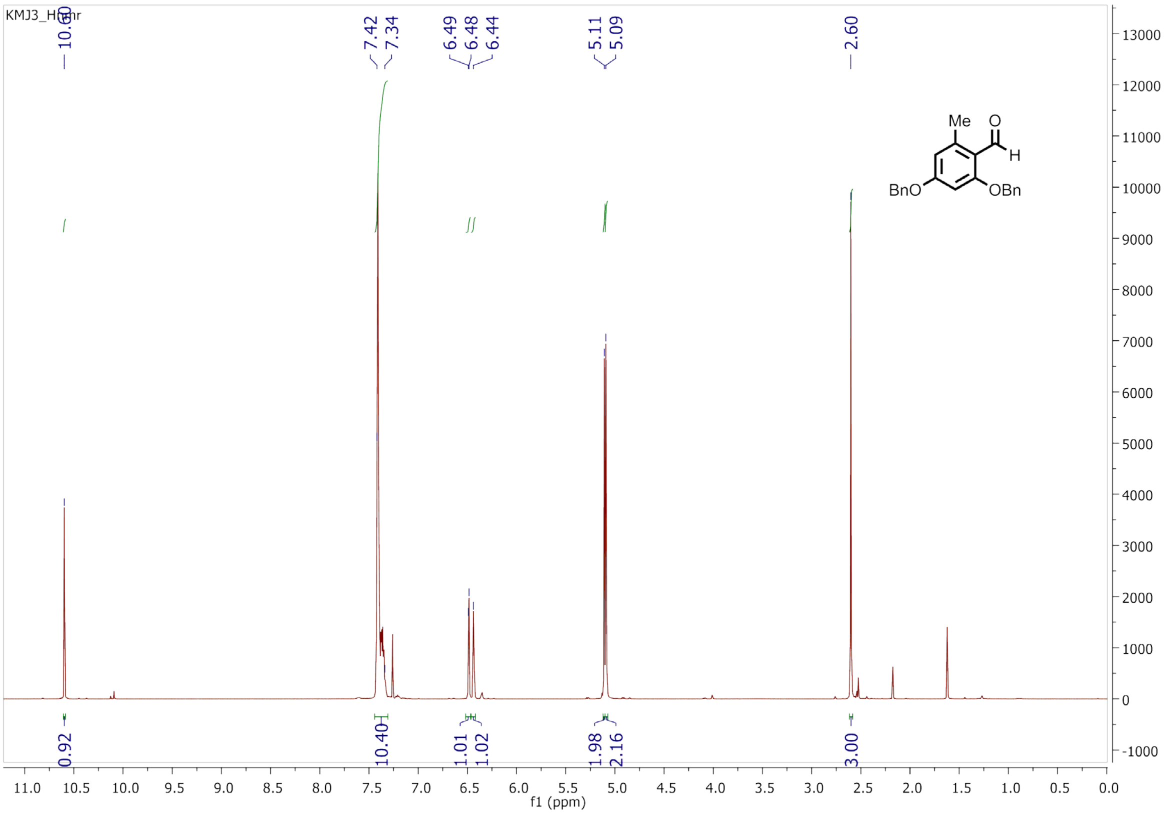

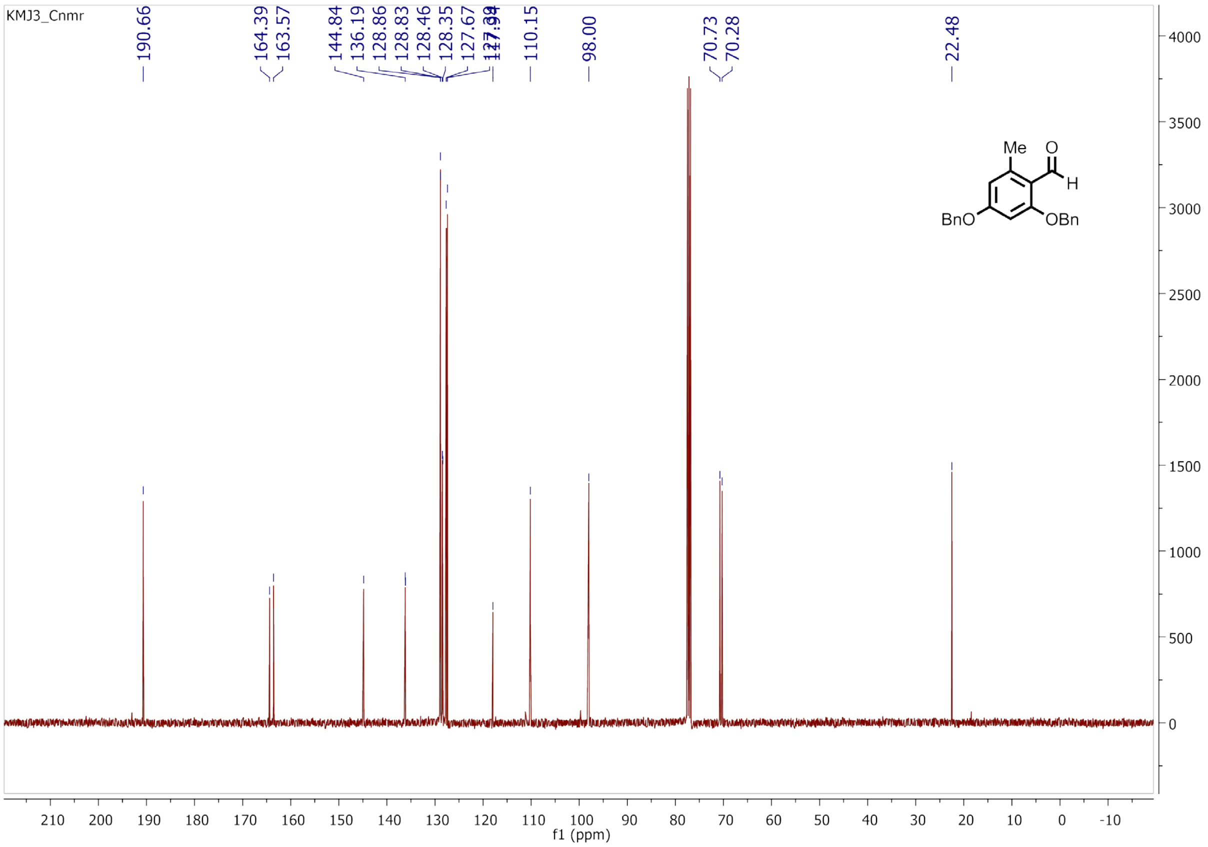

2,4-Bis-(benzyloxy)-6-methylbenzoic acid (6)

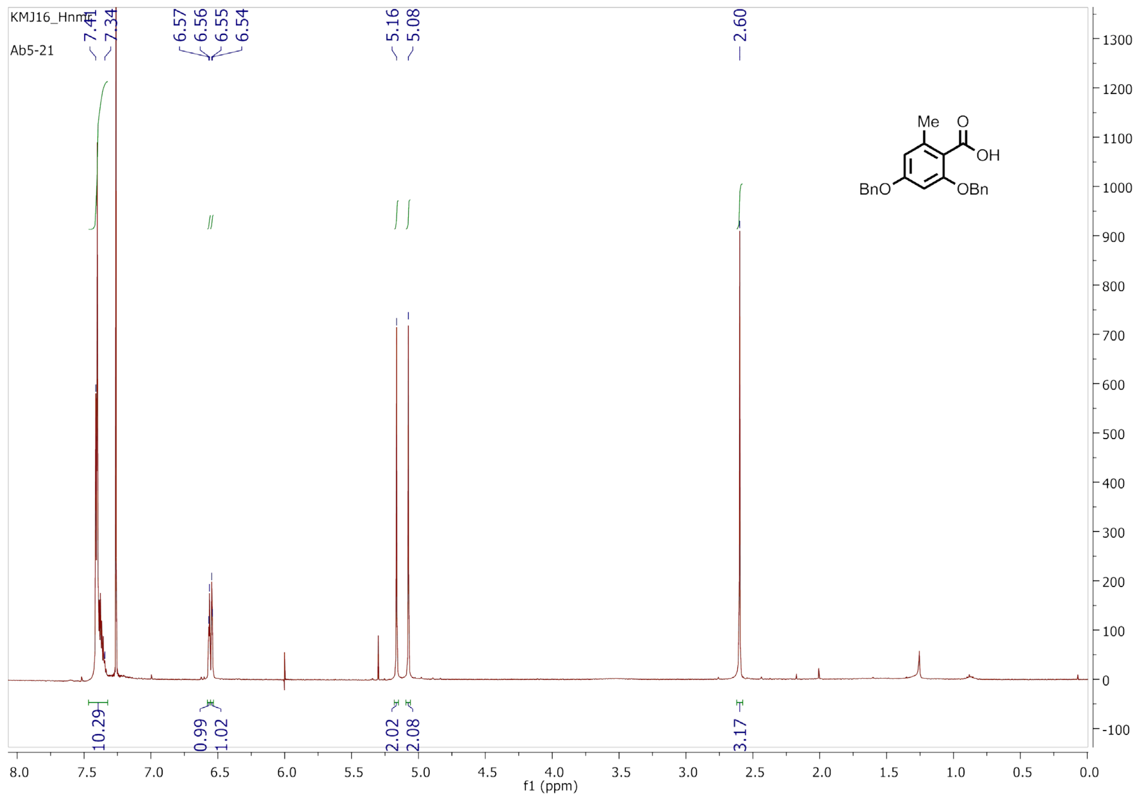

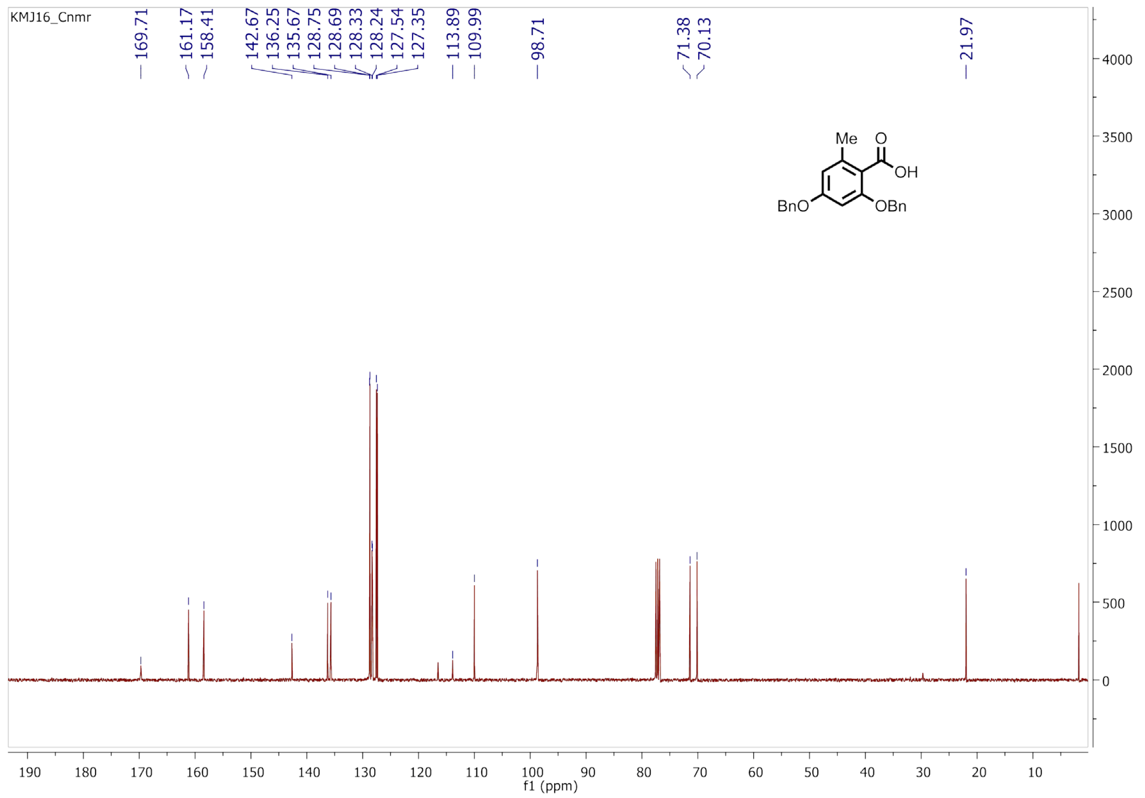

6,6'-di-(trityloxy)-α,α-trehalose

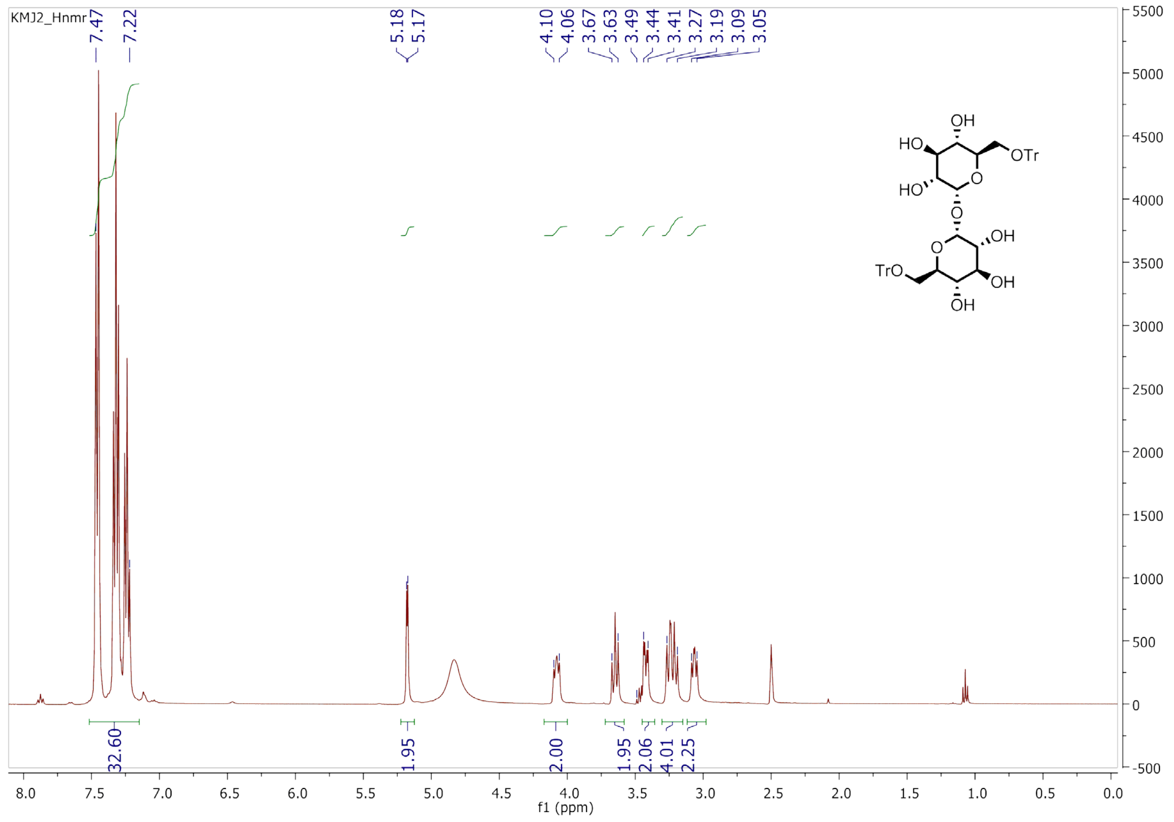

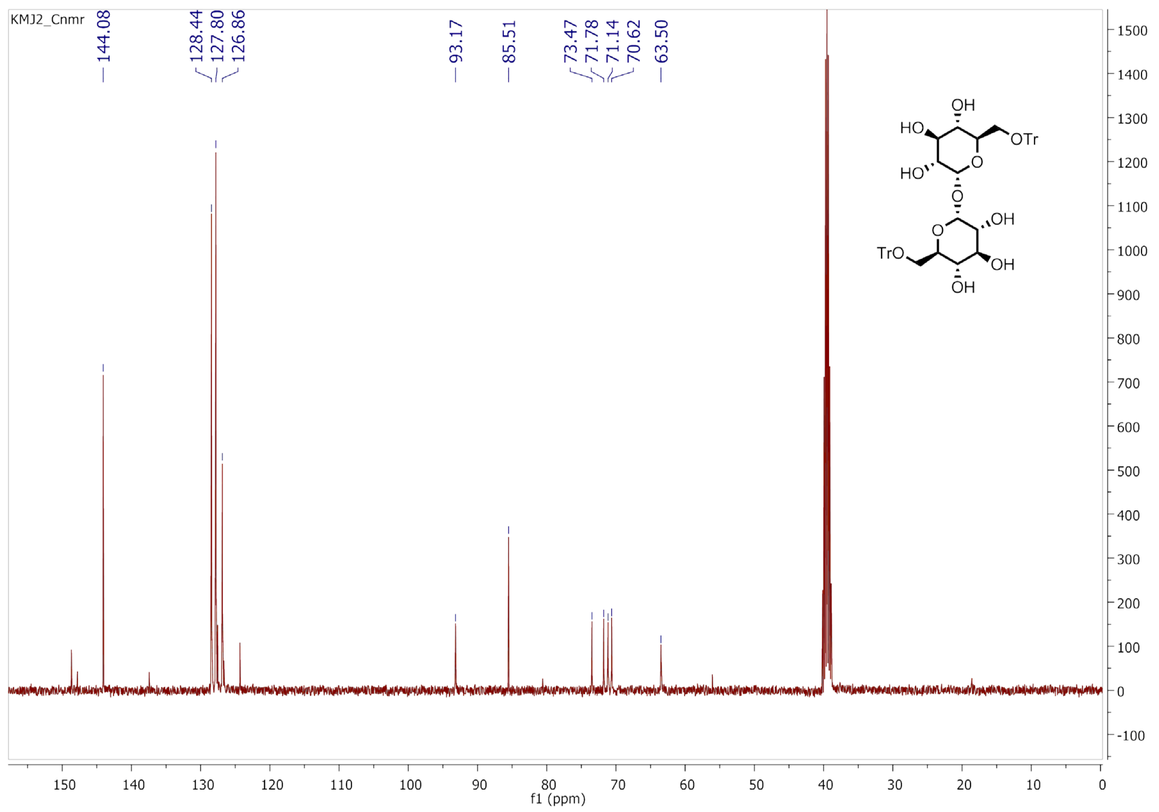

2,3,4,2’,3’,4’-Hexa-(benzyloxy)-α,α-trehalose

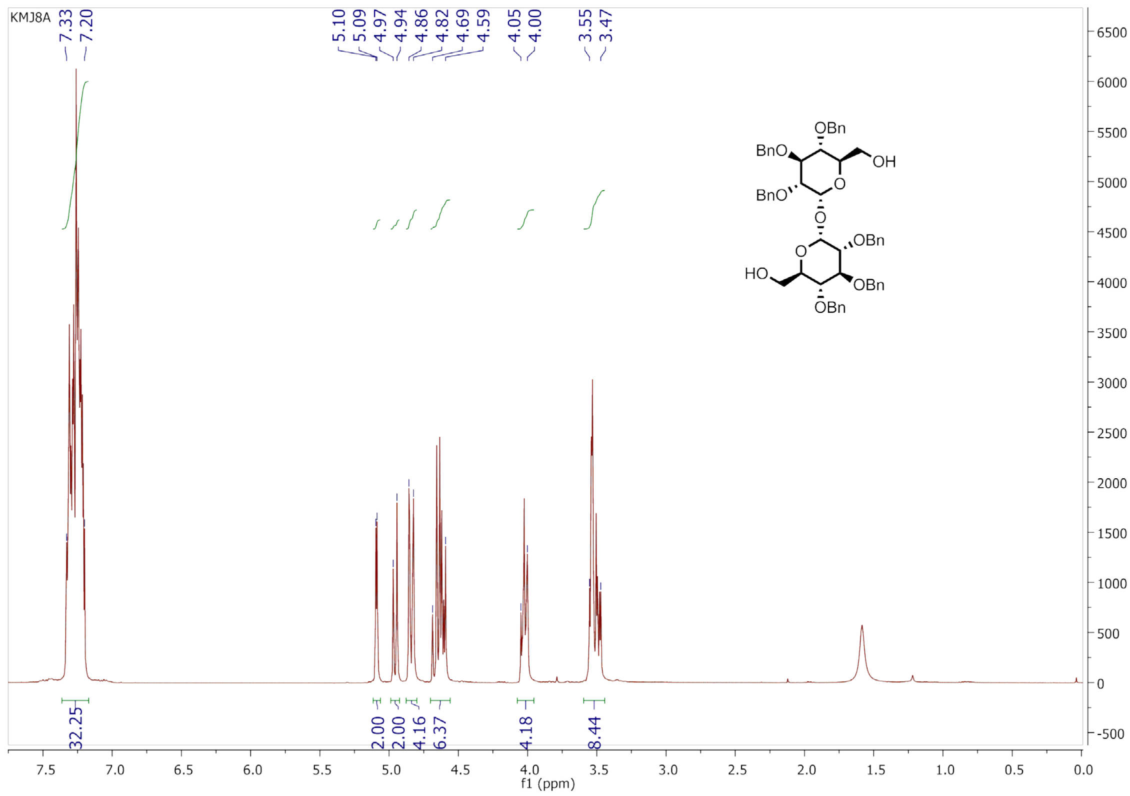

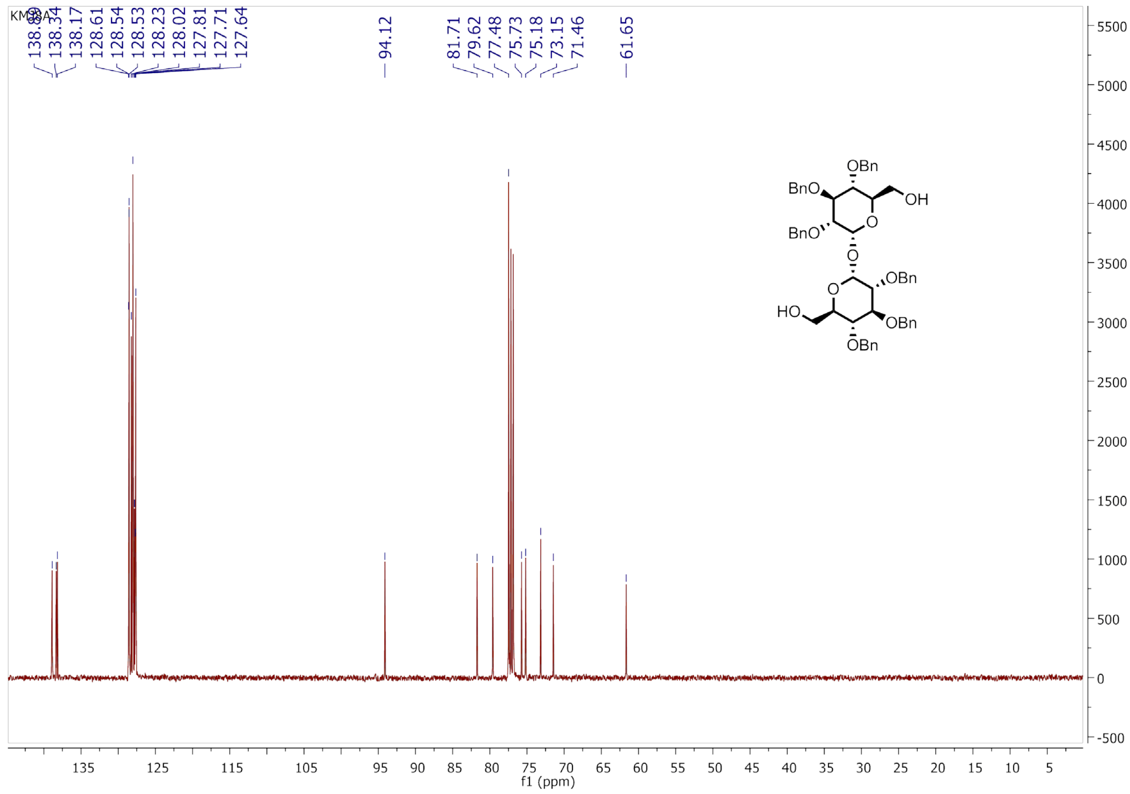

2,3,4,2’,3’,4’-Hexa-(benzyloxy)-α,β-trehalose

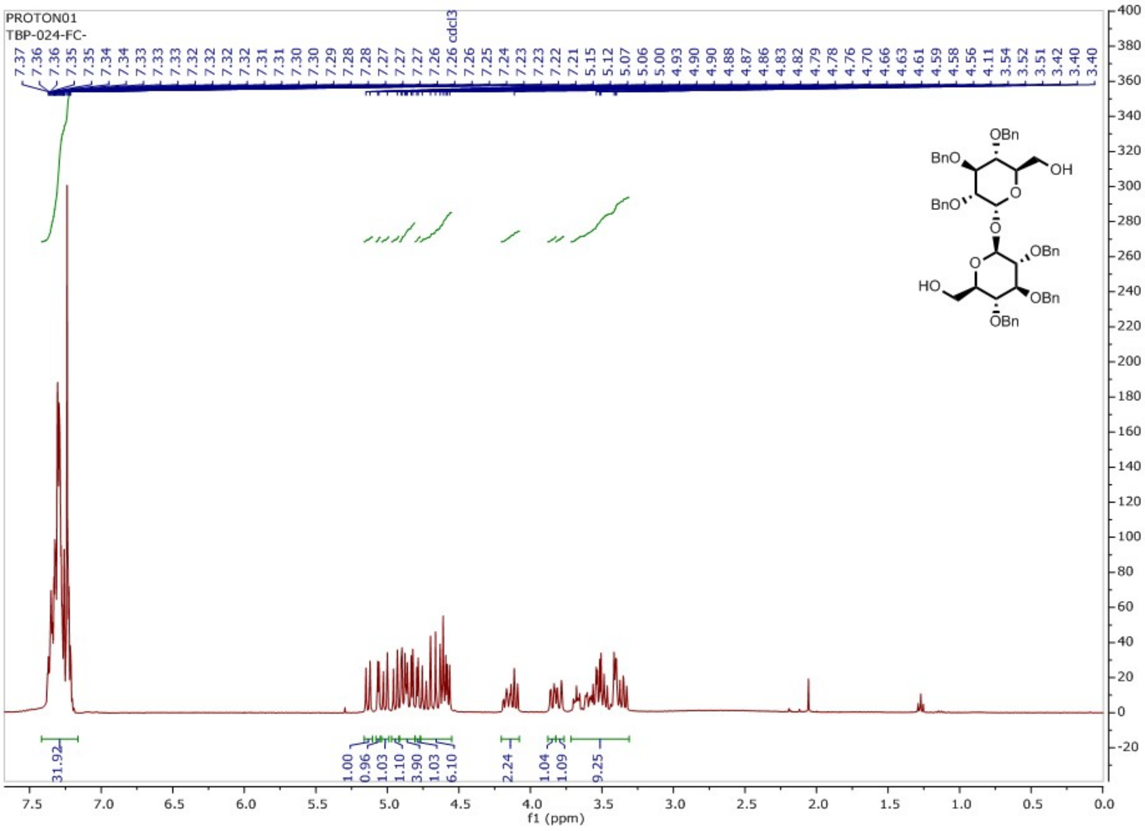

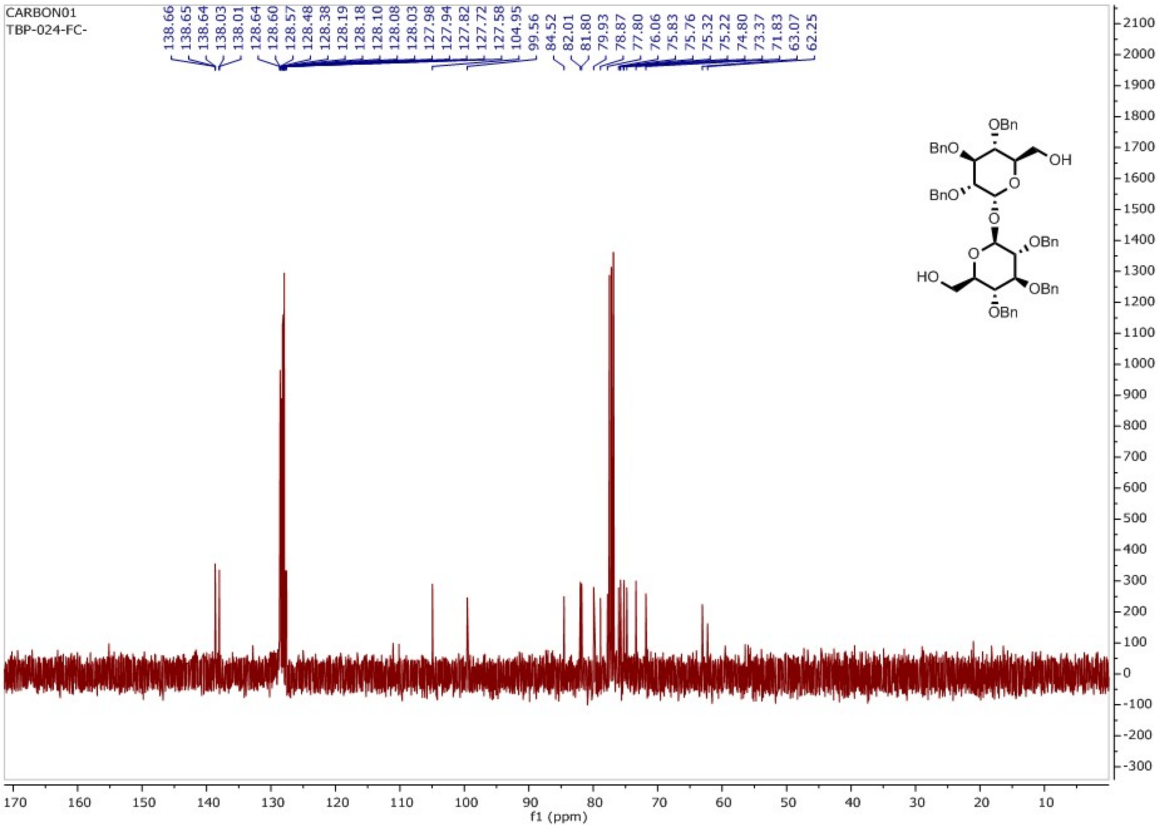

2,3,4,2′,3′,4′-Hexa-(benzyloxy)-6-acetyloxy-α,α-trehalose

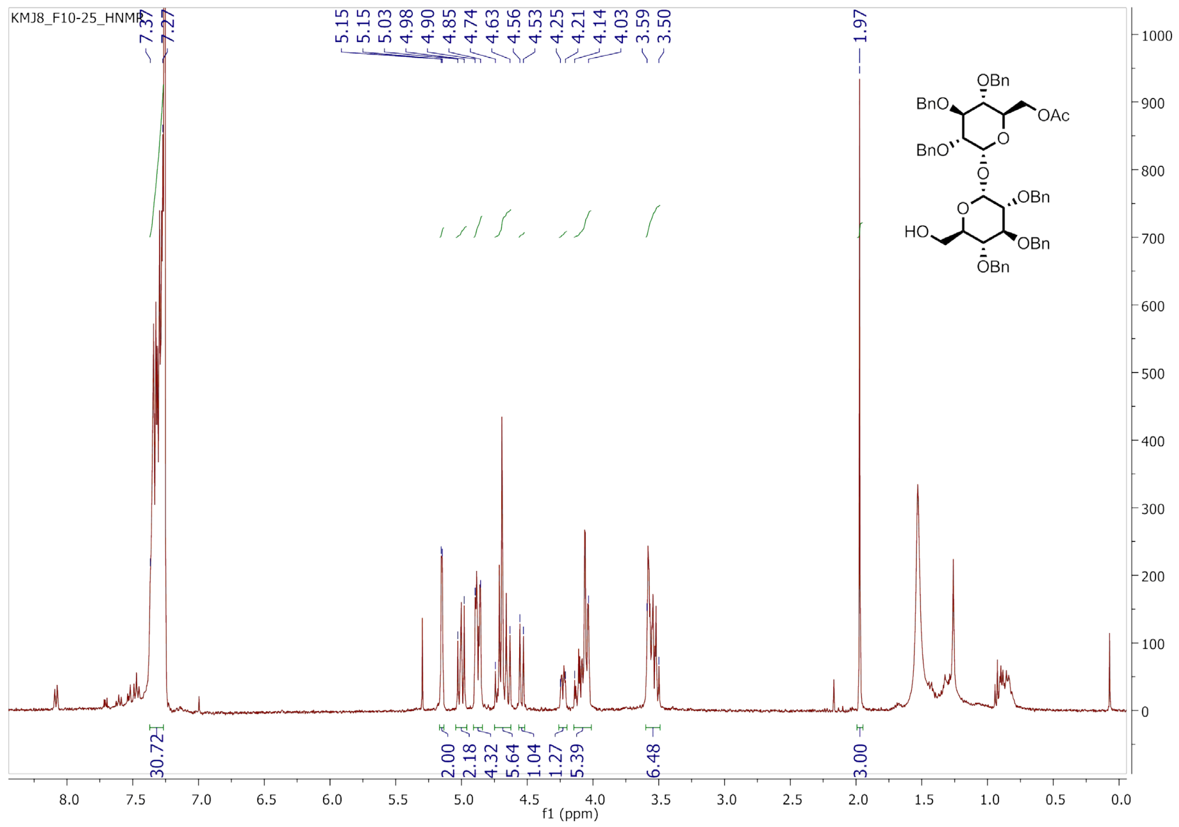

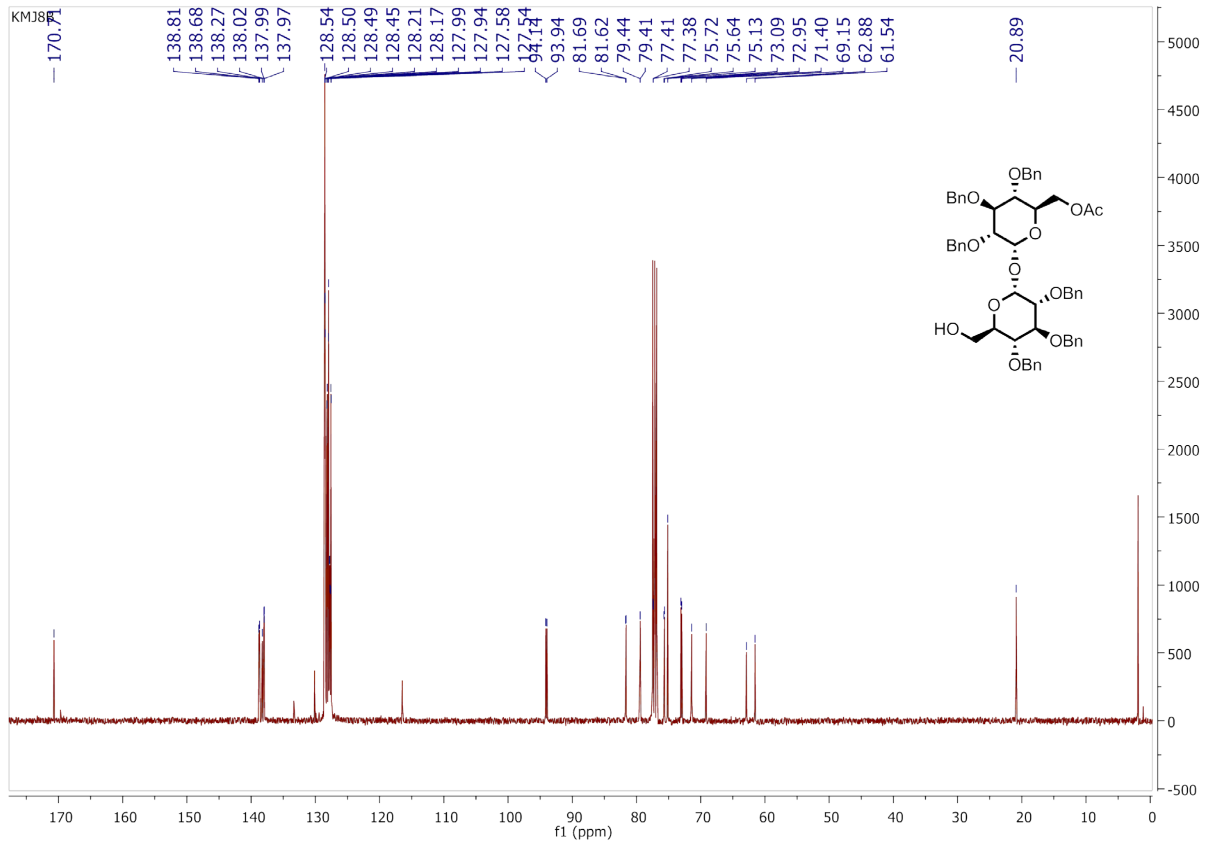

2,3,4,2',3',4'-Hexa-(benzyloxy)-6,6'-bis-(2,4-bis-(benzyloxy)-6-methylbenzoate)-α,α-trehalose

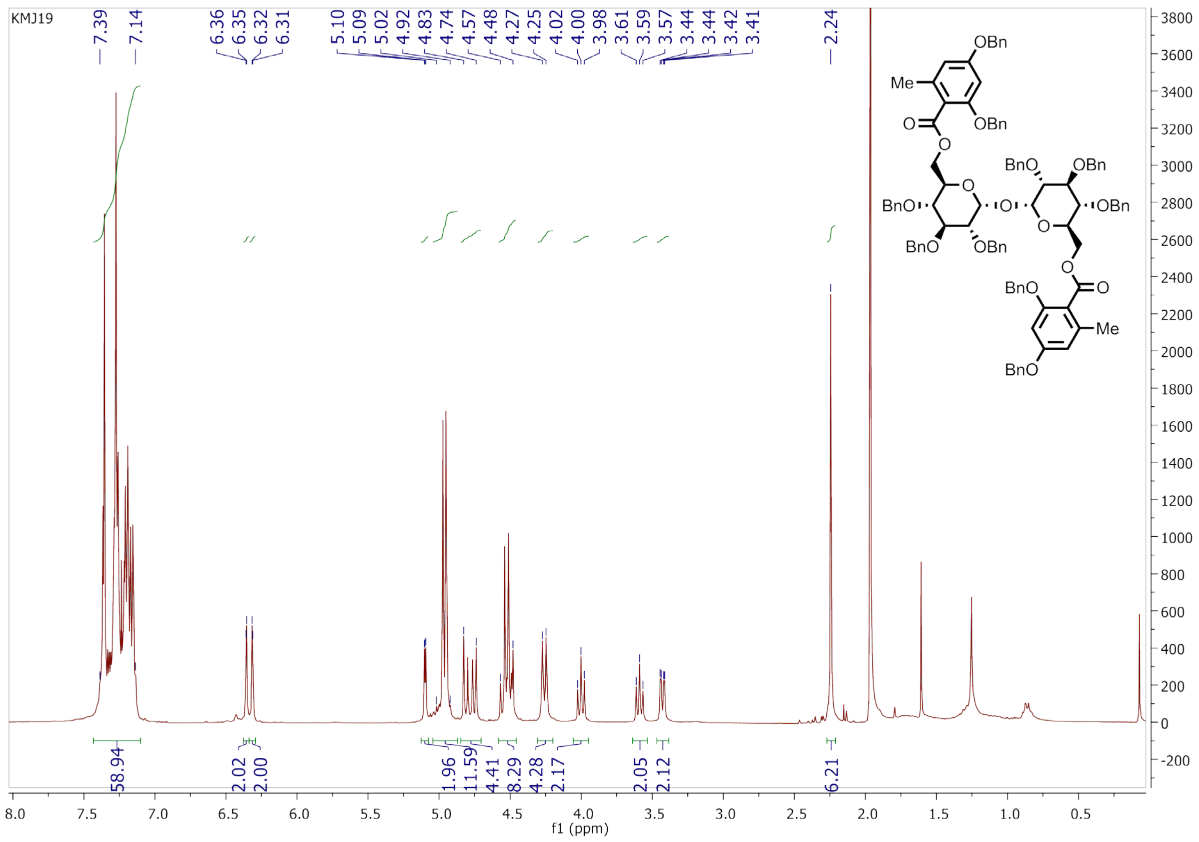

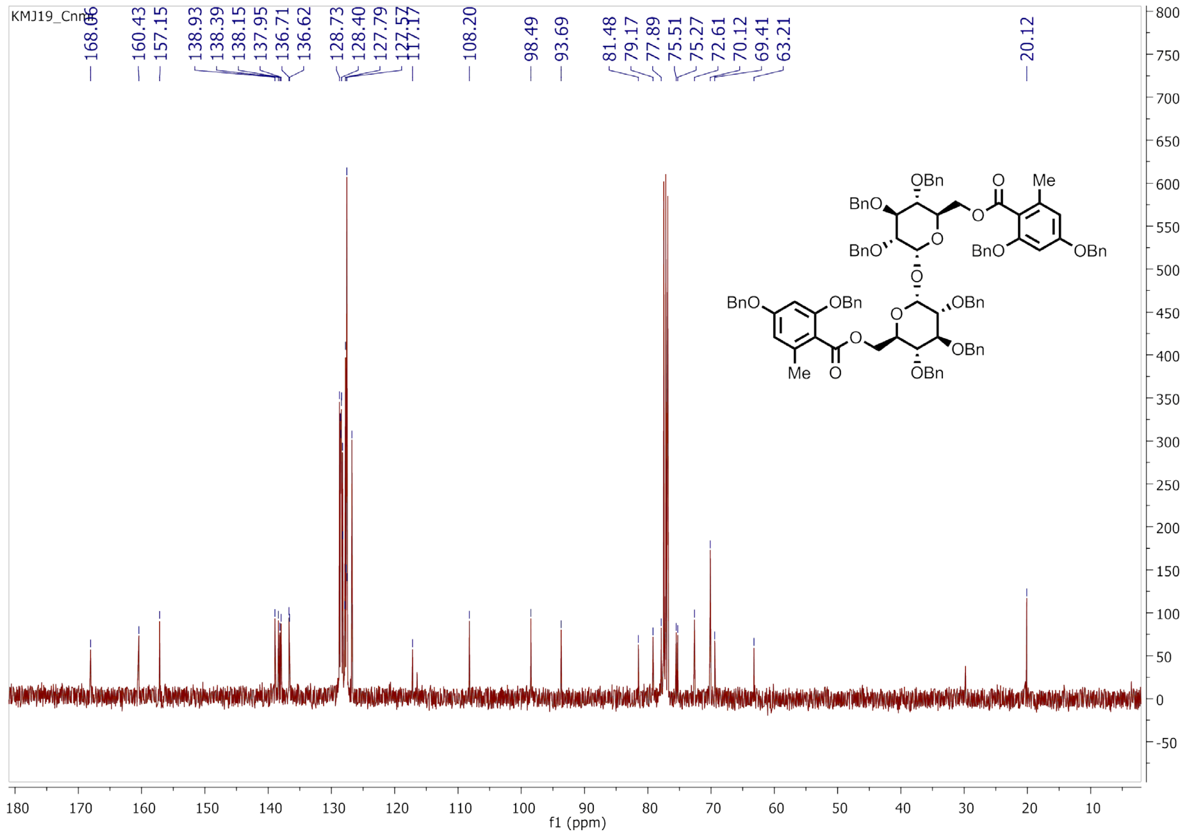

2,3,4,2’,3’,4’-Hexa-(benzyloxy)-6-acetyloxy-6’-(2,4-bis-(benzyloxy)-6-methylbenzoate)-α,α-trehalose

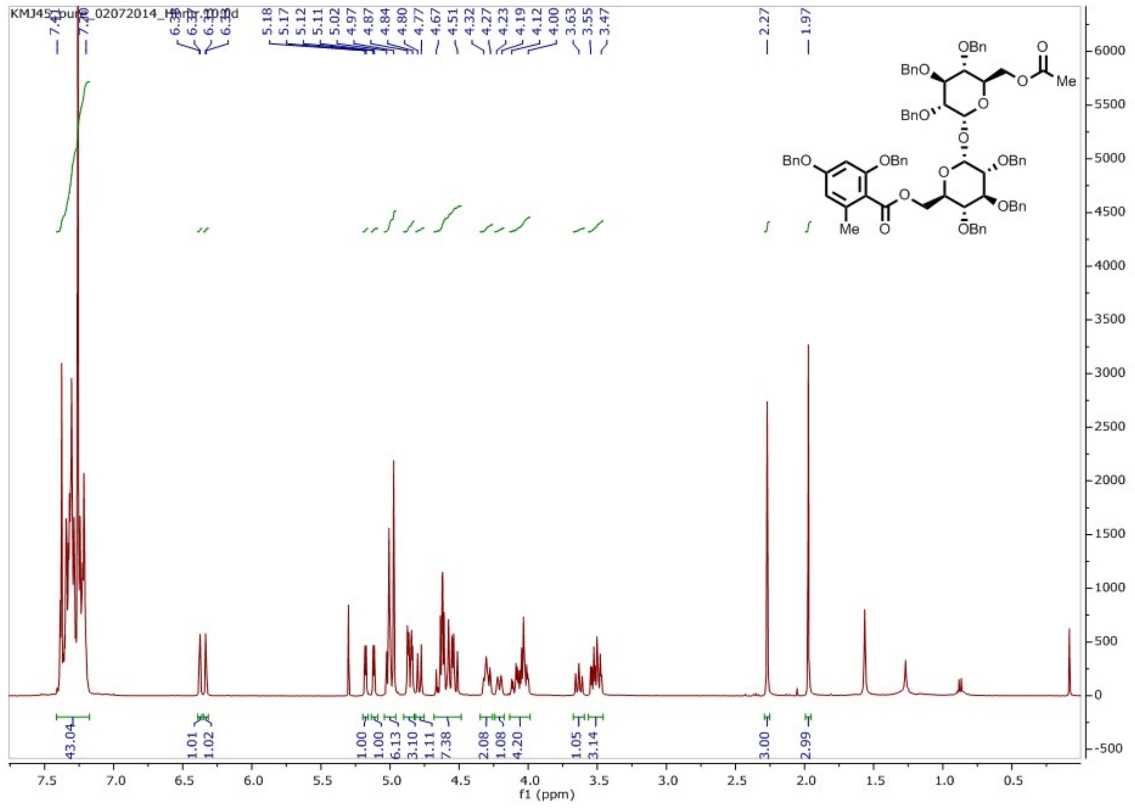

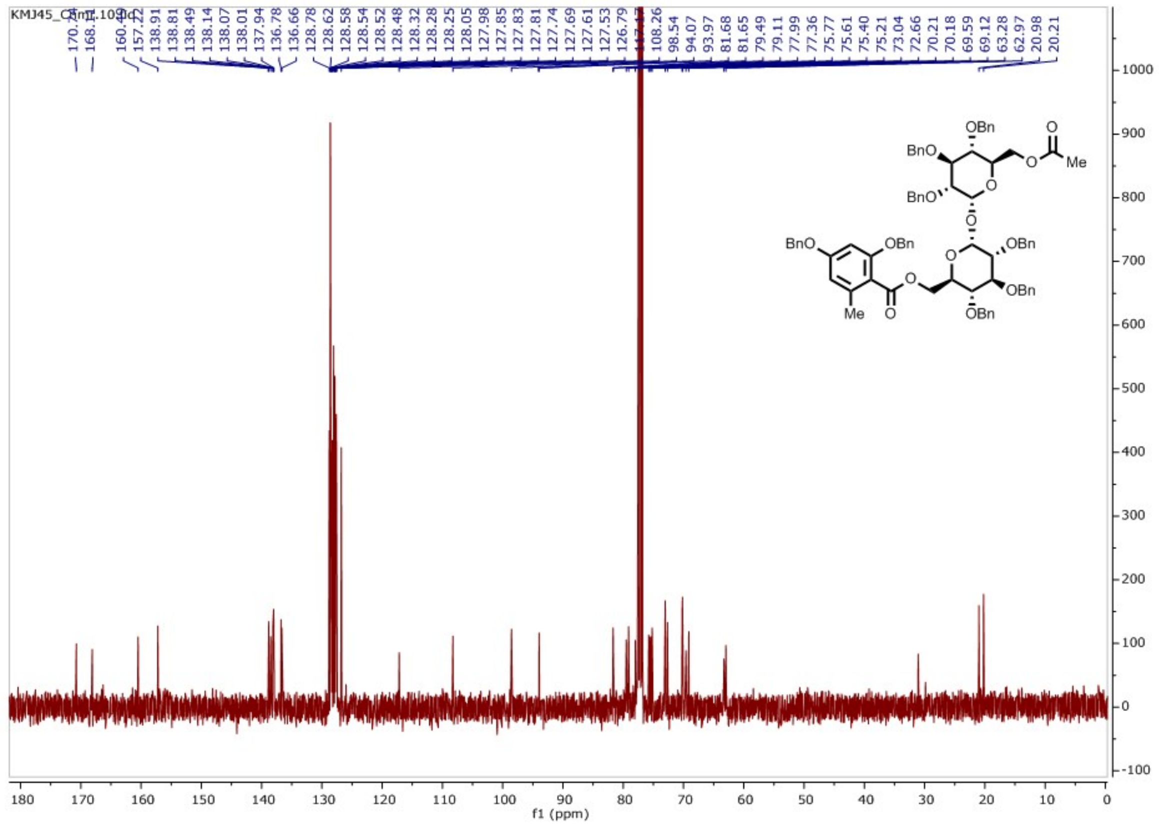

2,3,4,2′,3′,4′-Hexa-(benzyloxy)-6′-(2,4-bis-(benzyloxy)-6-methylbenzoate)-α,α-trehalose

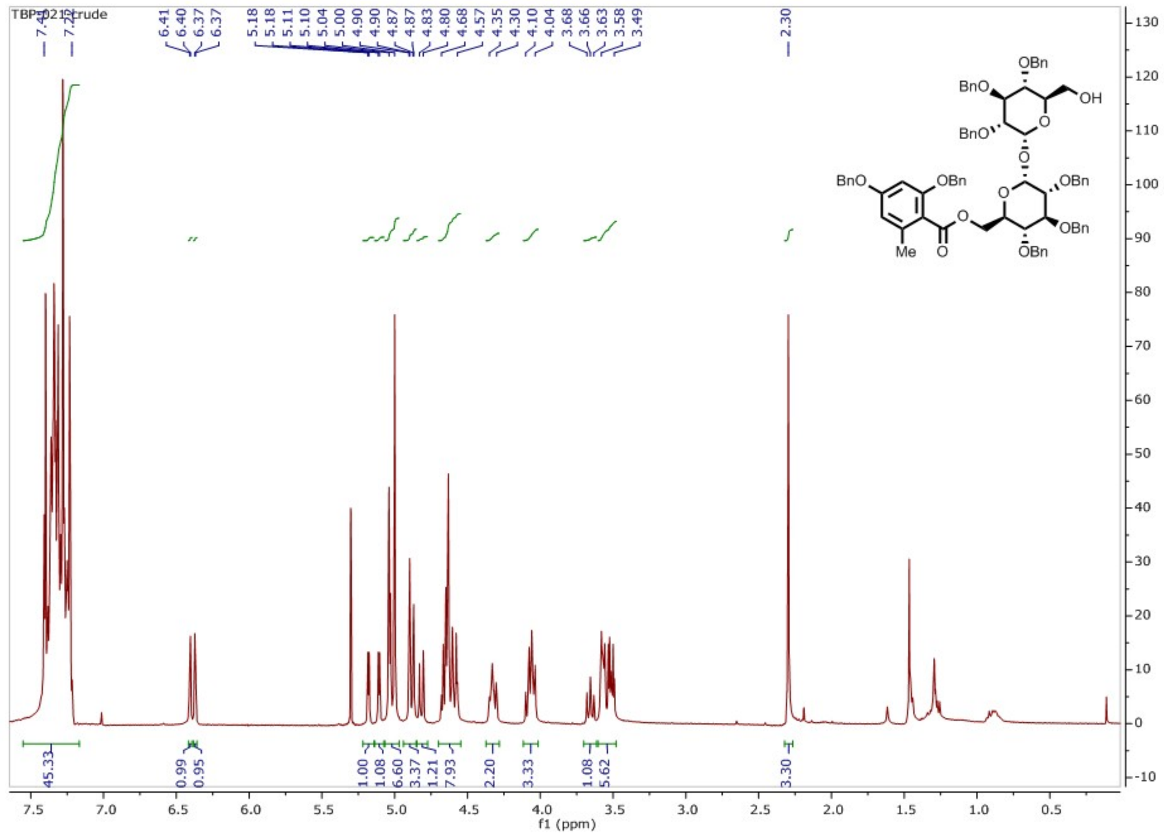

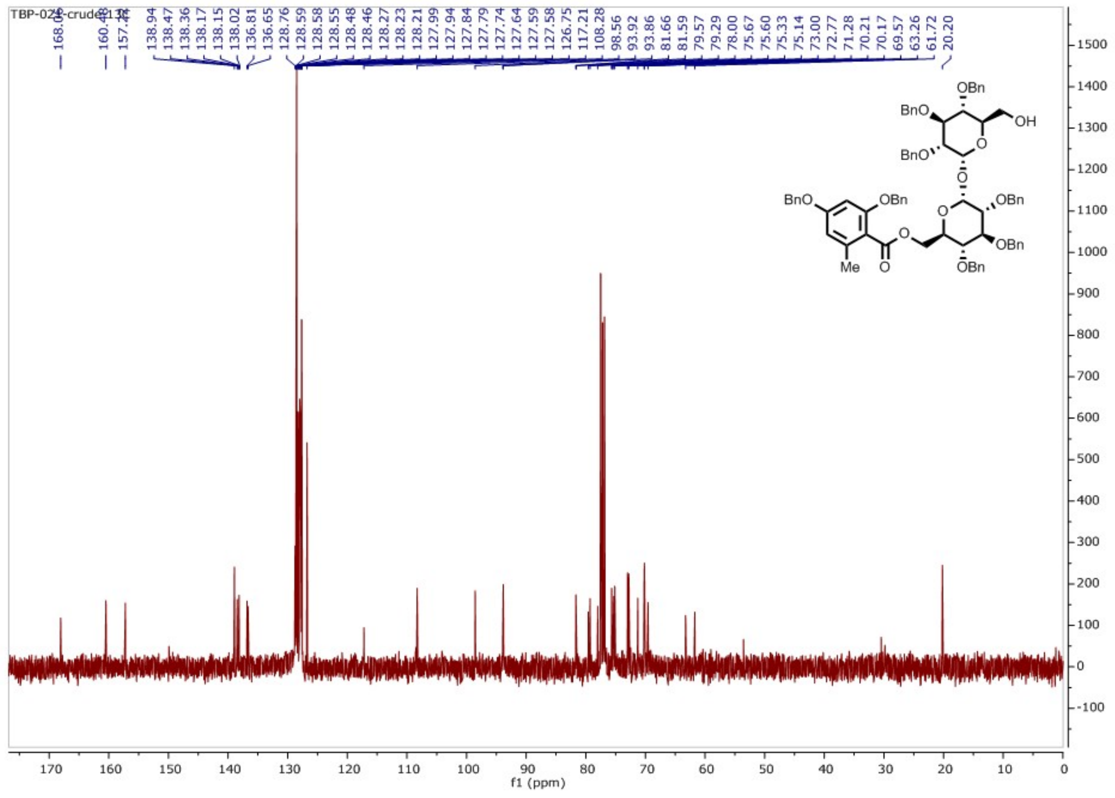

2,3,4,2',3',4'-Hexa-(benzyloxy)-6,6'-bis-(benzoate)-α,α-trehalose

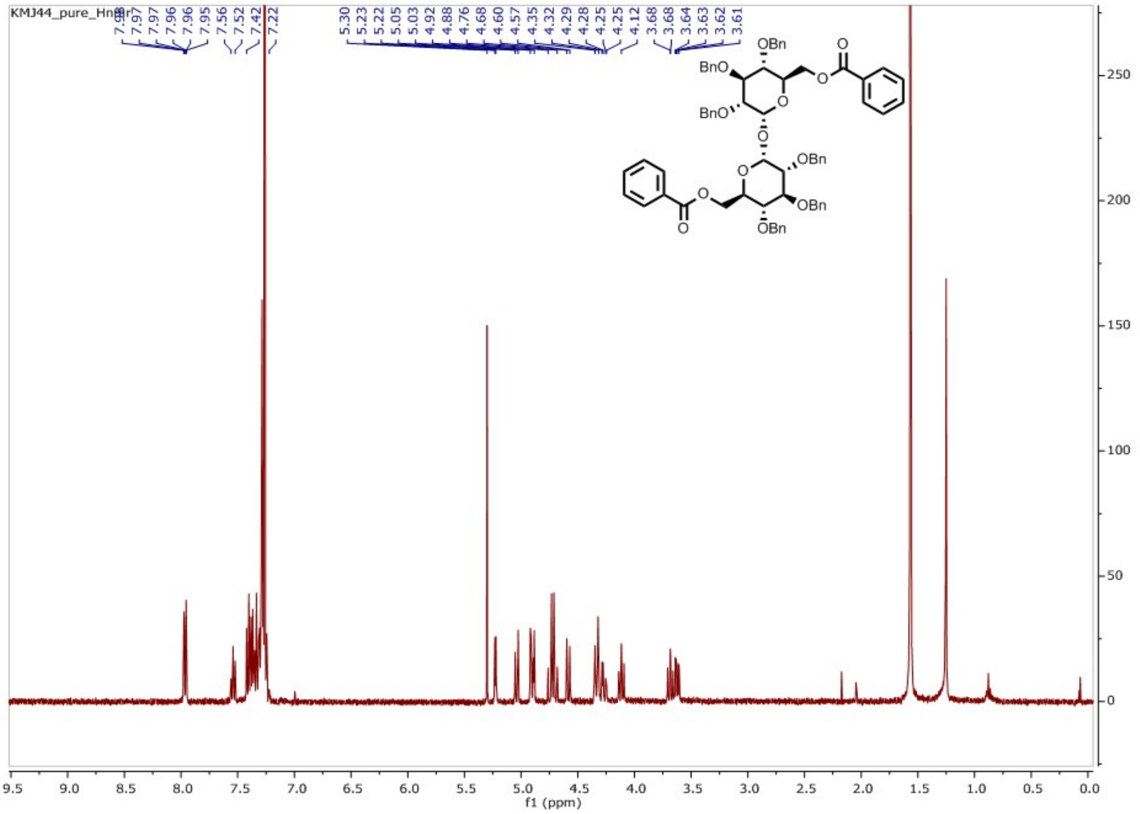

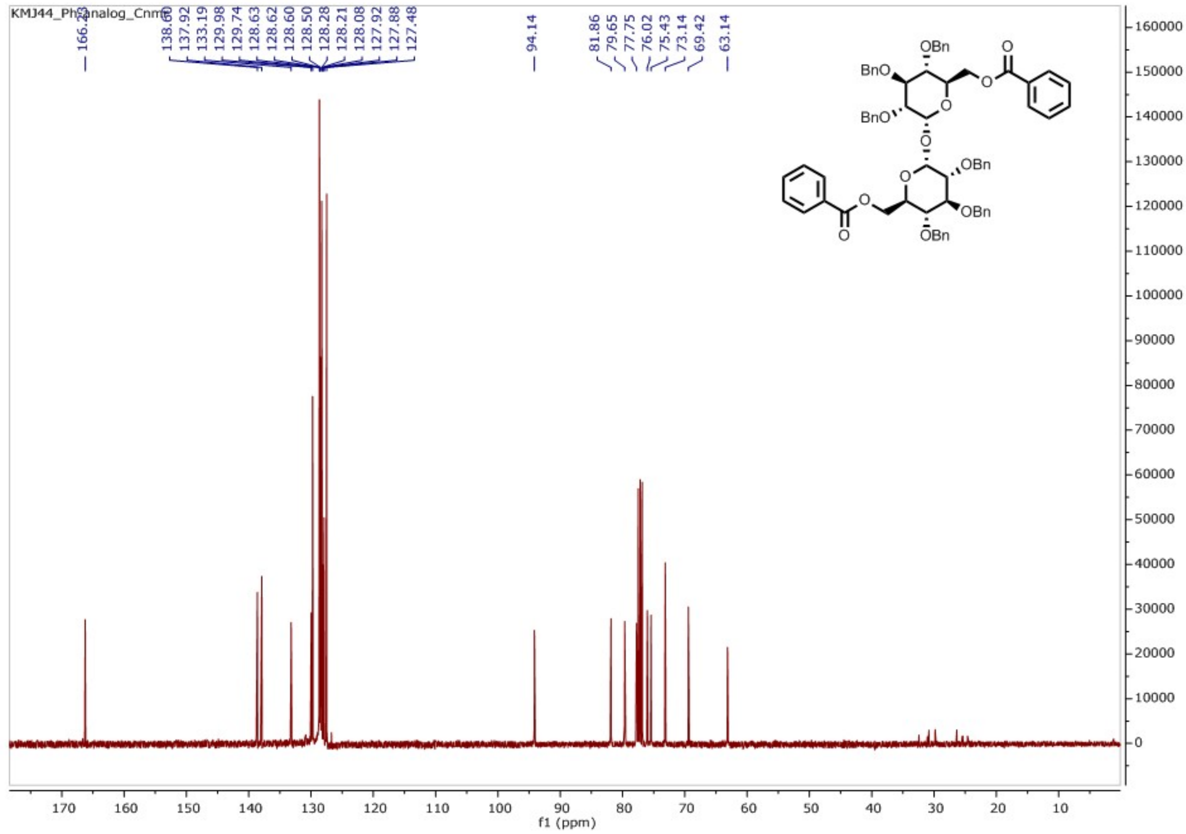

2,3,4,2’,3’,4’-Hexa-(benzyloxy)-6,6’-bis-(2,3-methoxybenzoate)-α,α-trehalose

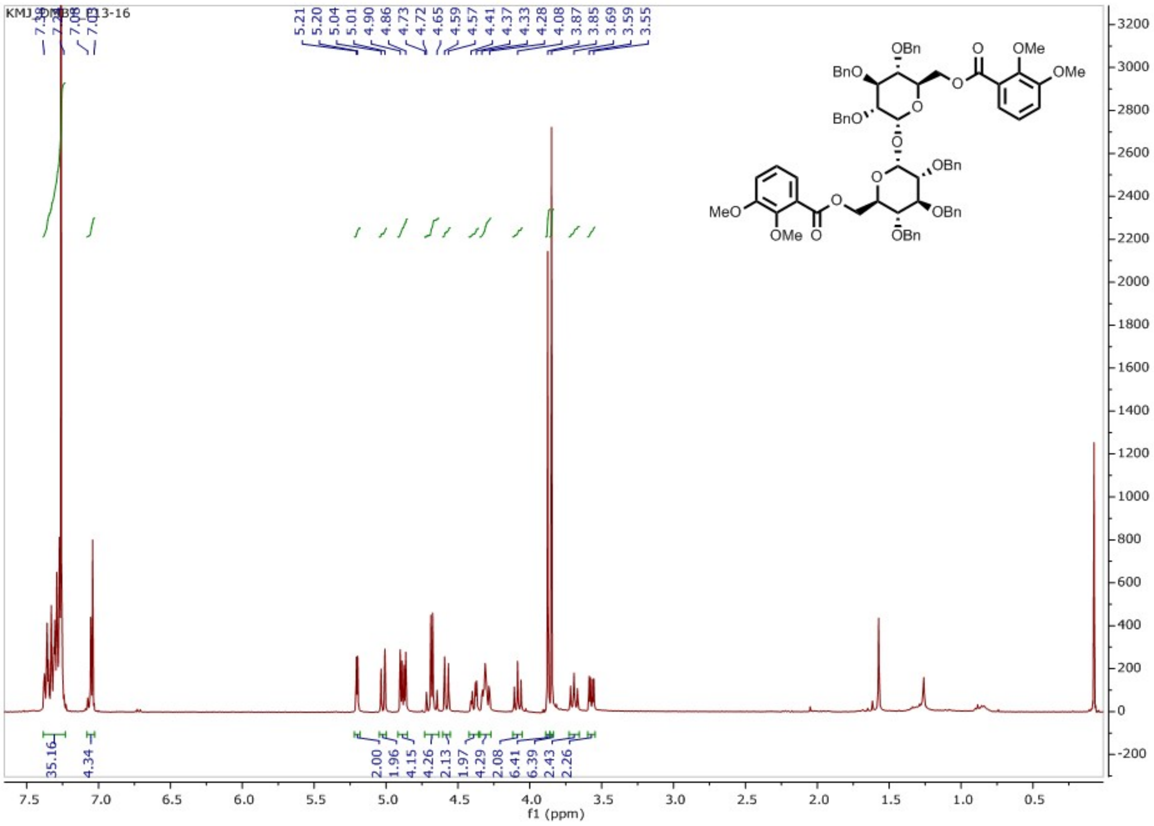

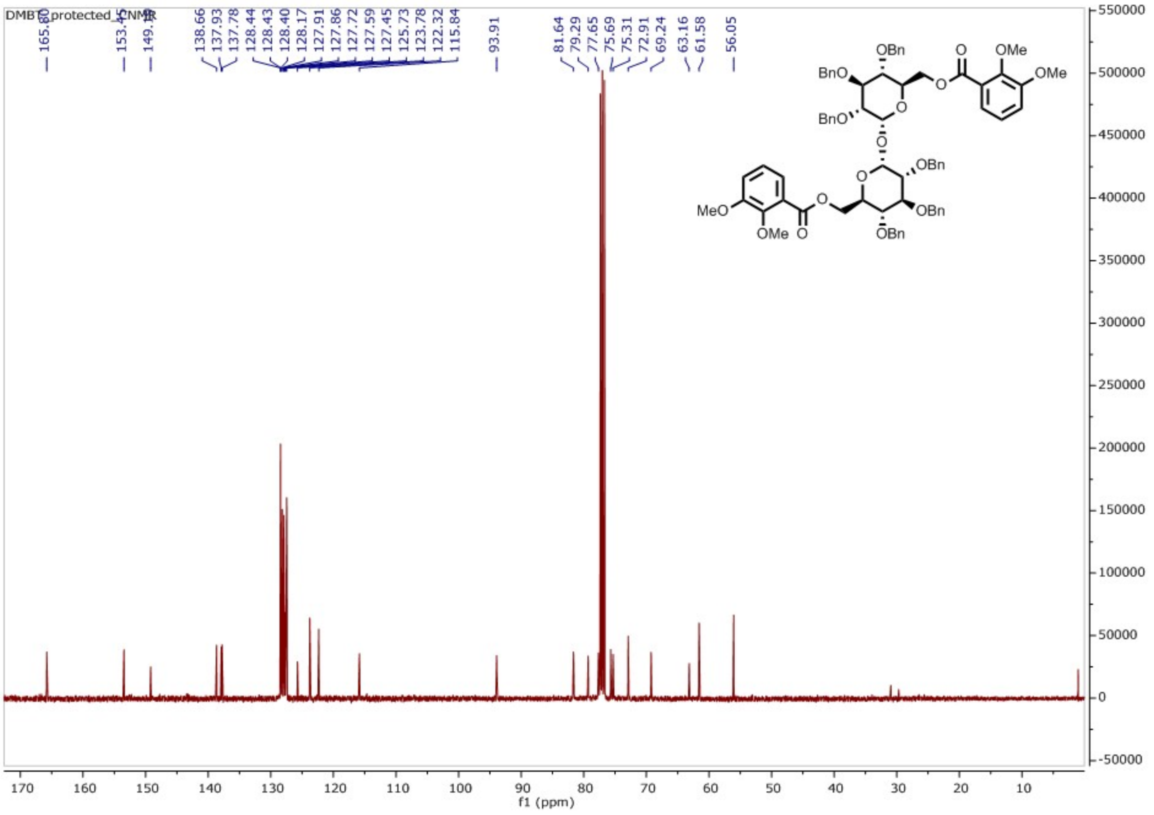

2,3,4,2′,3′,4′-Hexa-(benzyloxy)-6,6′-bis-(2,4-bis-(benzyloxy)-6-methylbenzoate)-α,β-trehalose

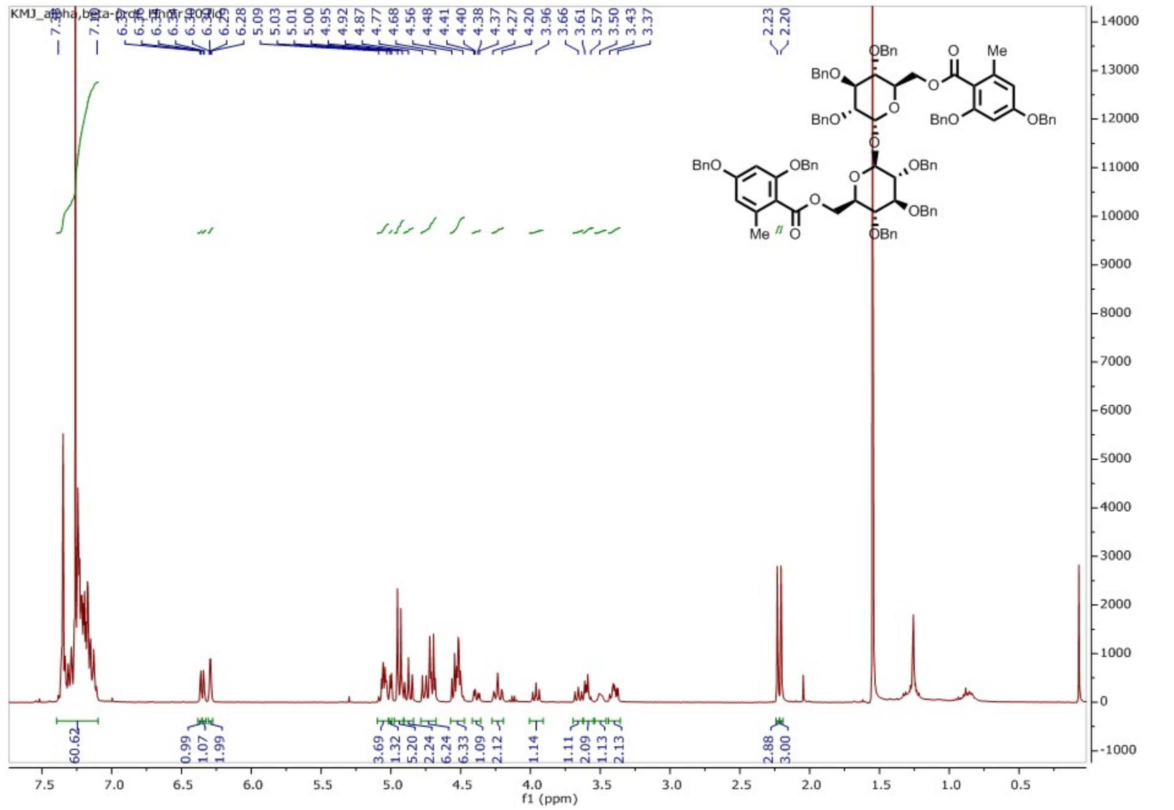

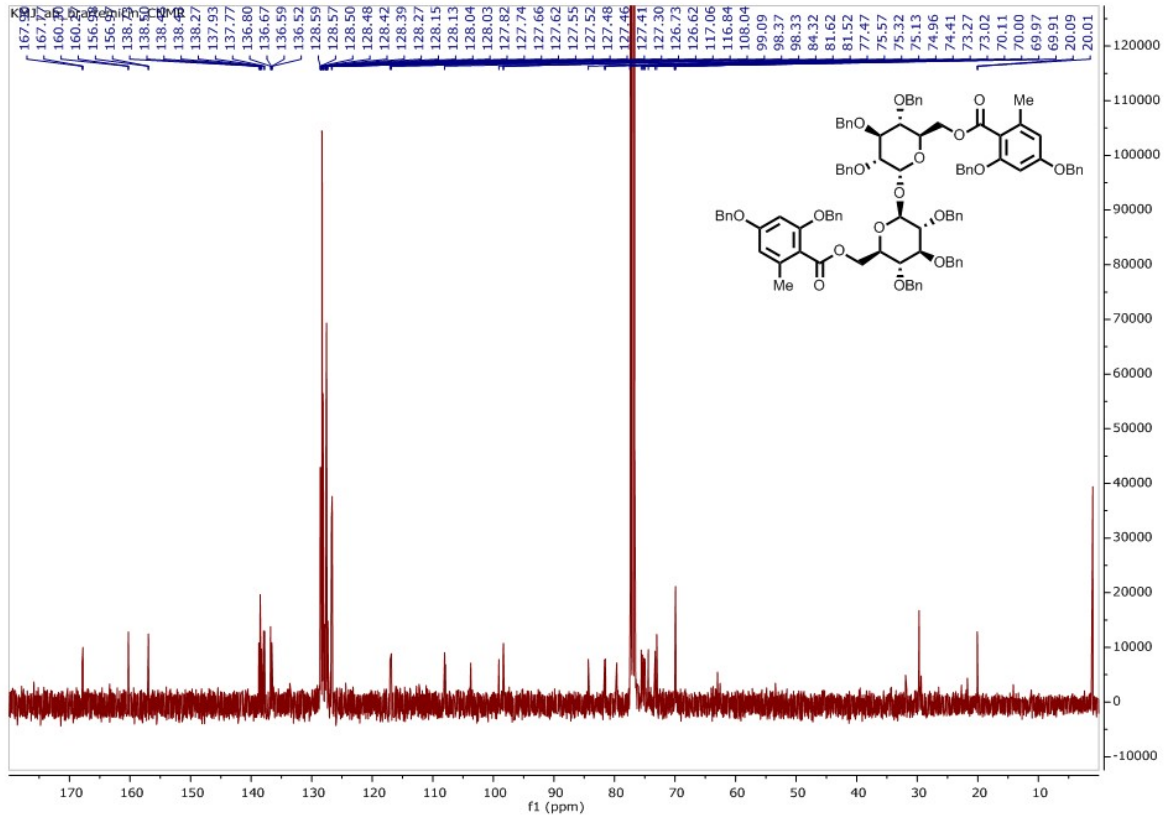

6,6'-Bis-(2,4-dihydroxy-6-methylbenzoate)-α,α-trehalose (1)

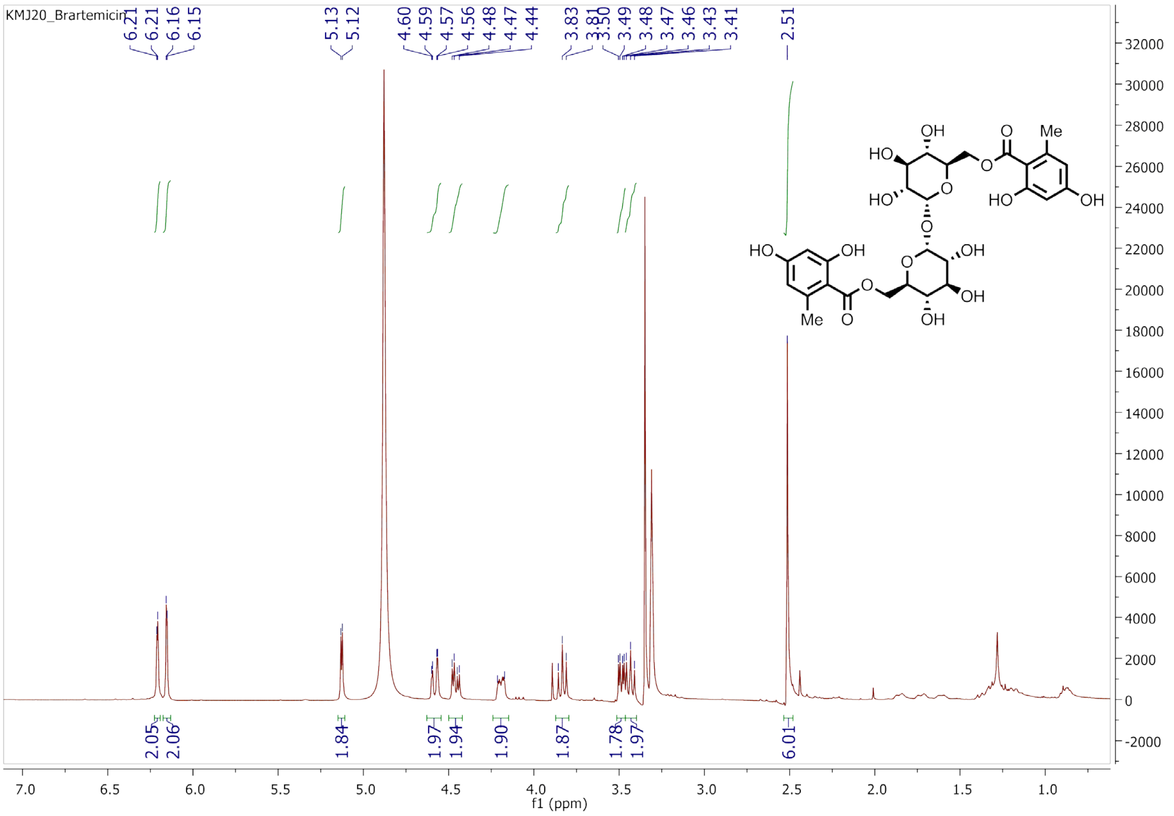

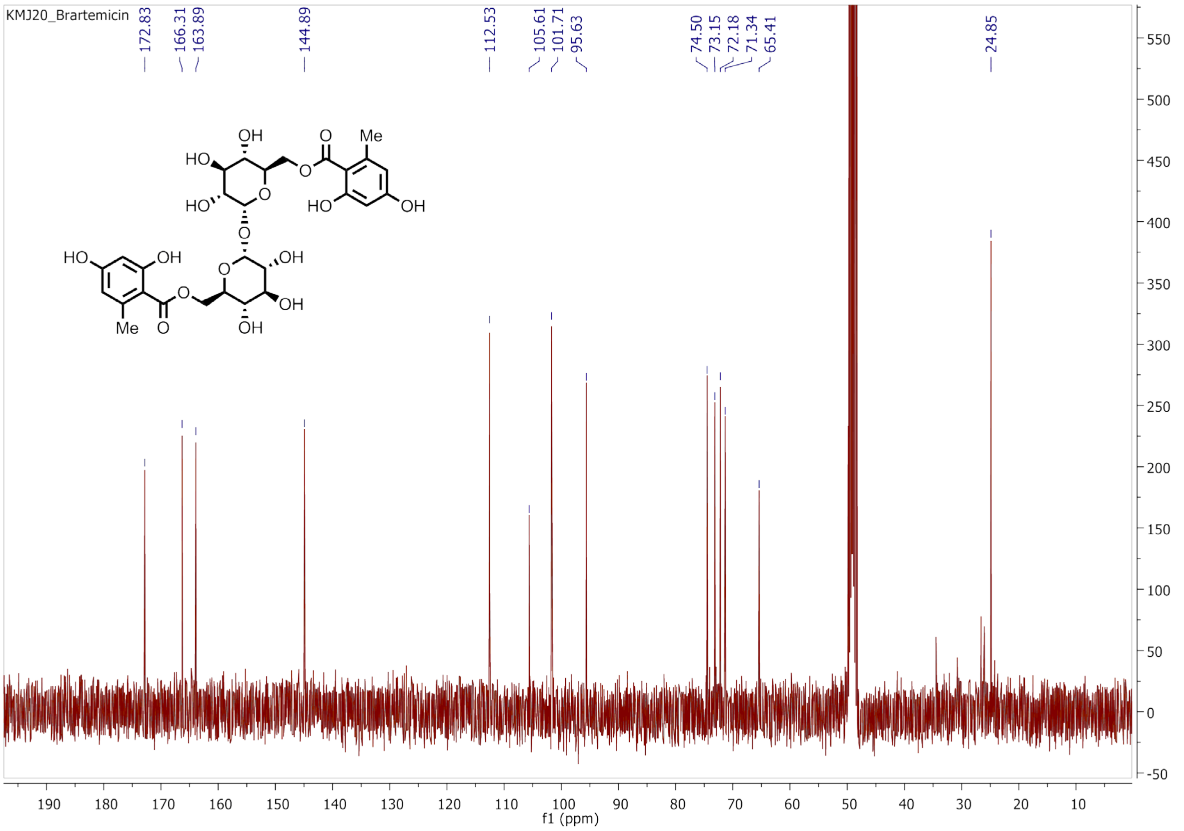

6,6'-Bis-(2,3-dimethoxybenzoate)-α,α-trehalose (2)

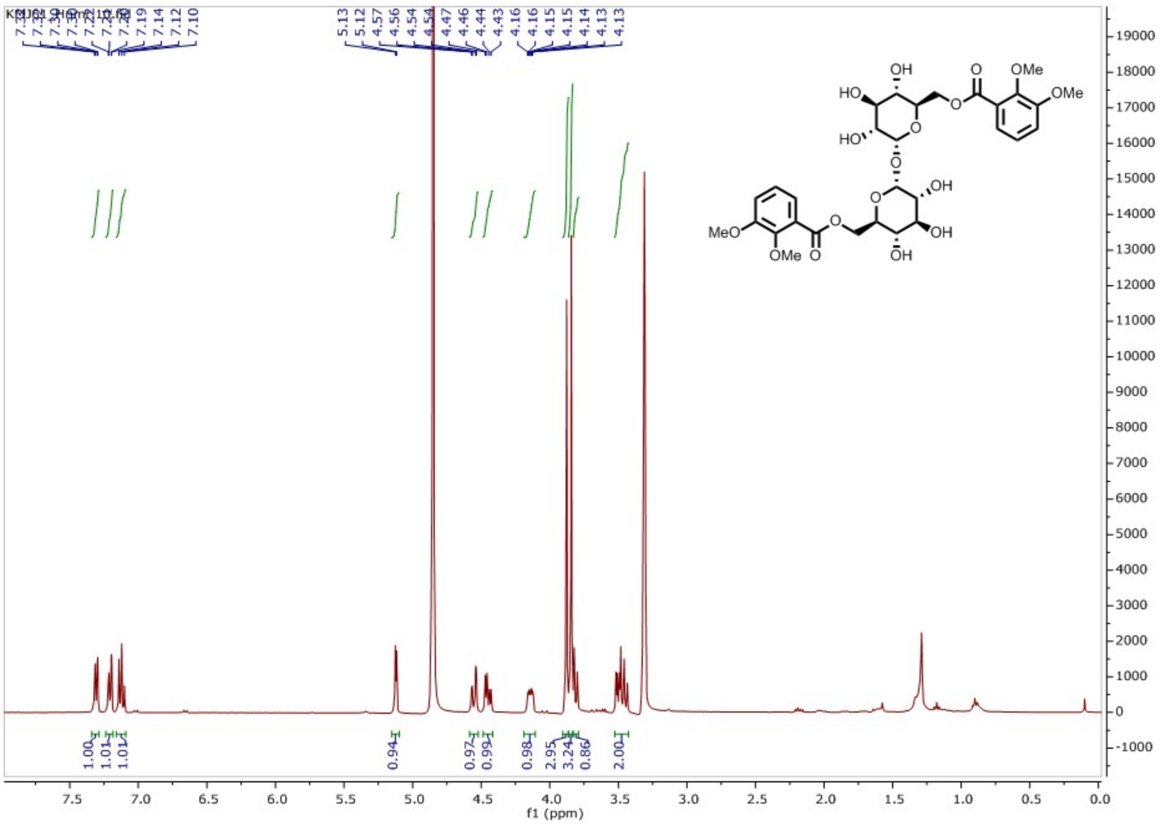

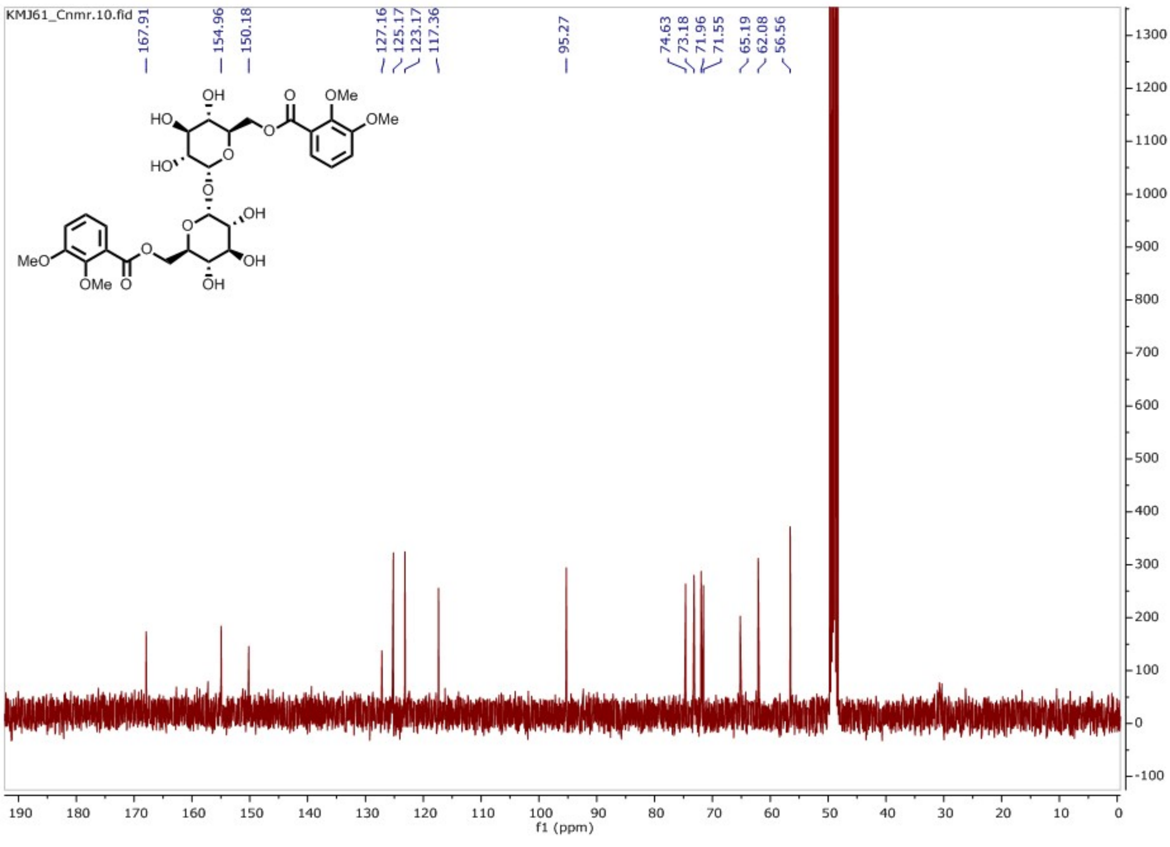

6-(2,4-dihydroxy-6-methylbenzoate)-α,α-trehalose (4)

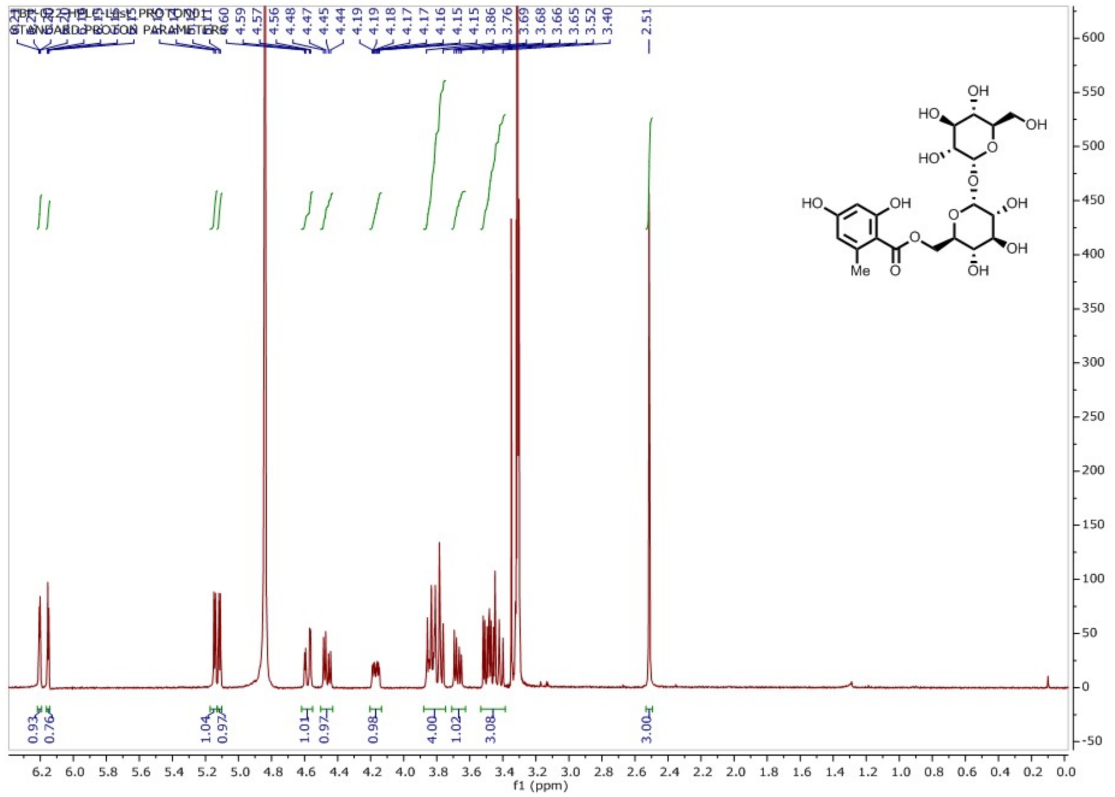

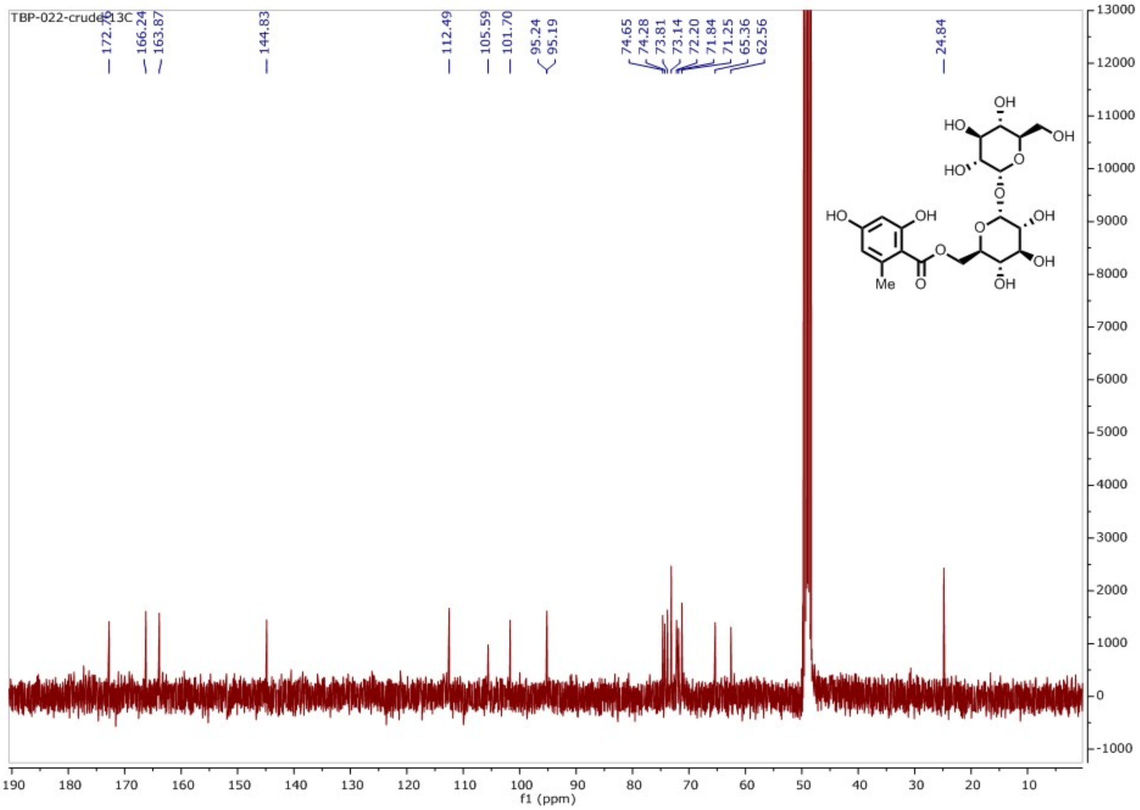

6,6'-Bis-(benzoate)-α,α-trehalose (3)

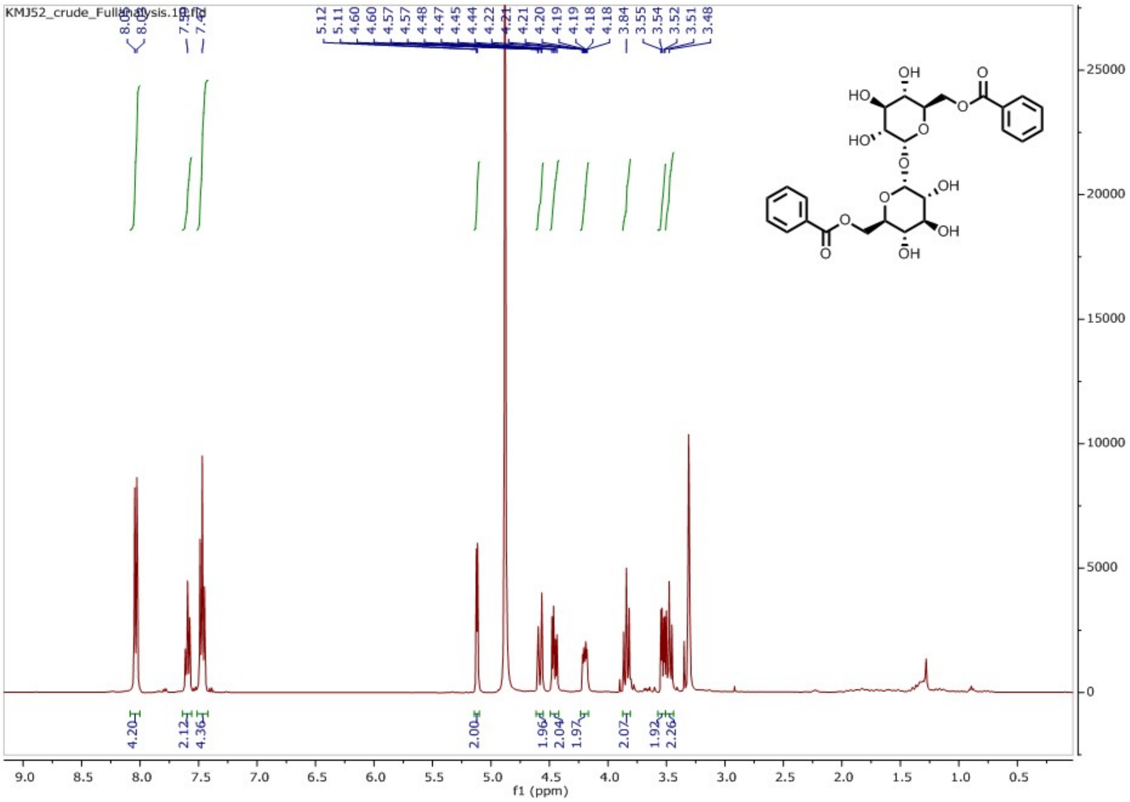

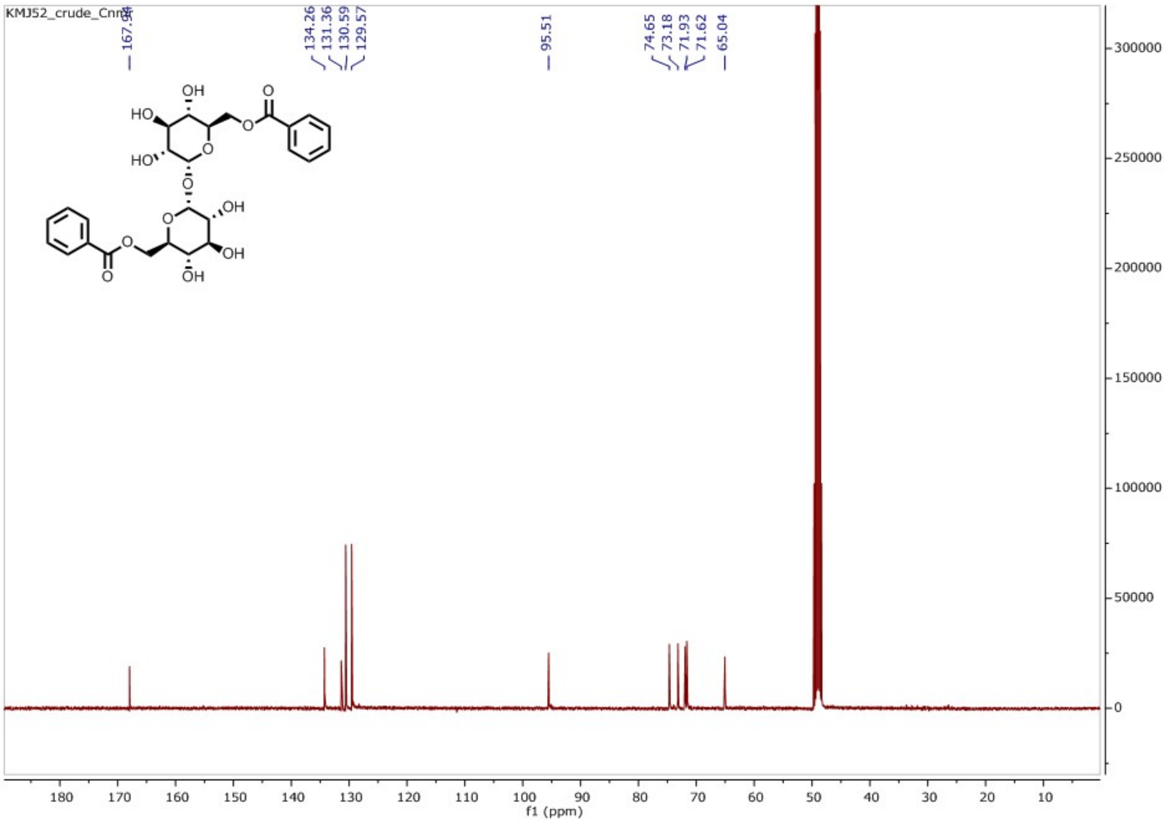

6,6’-Bis-(2,4-dihydroxy-6-methylbenzoate)-α,β-trehalose

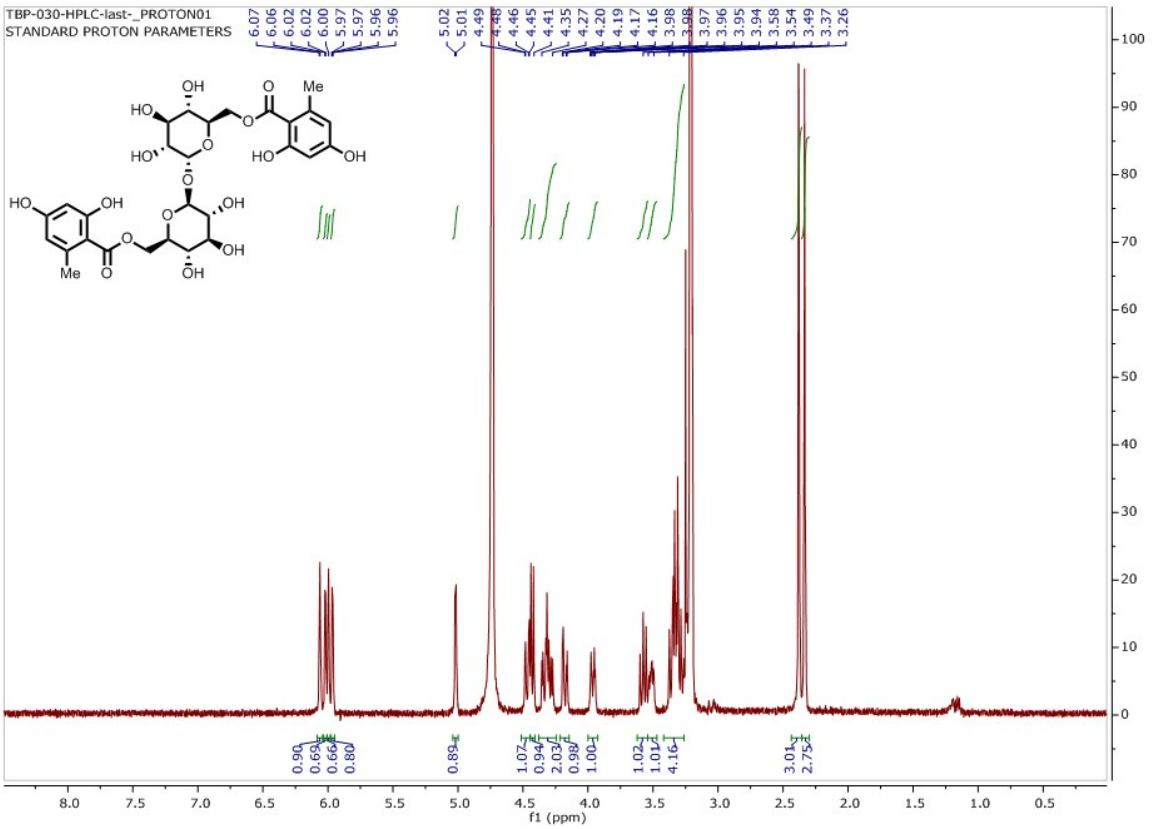

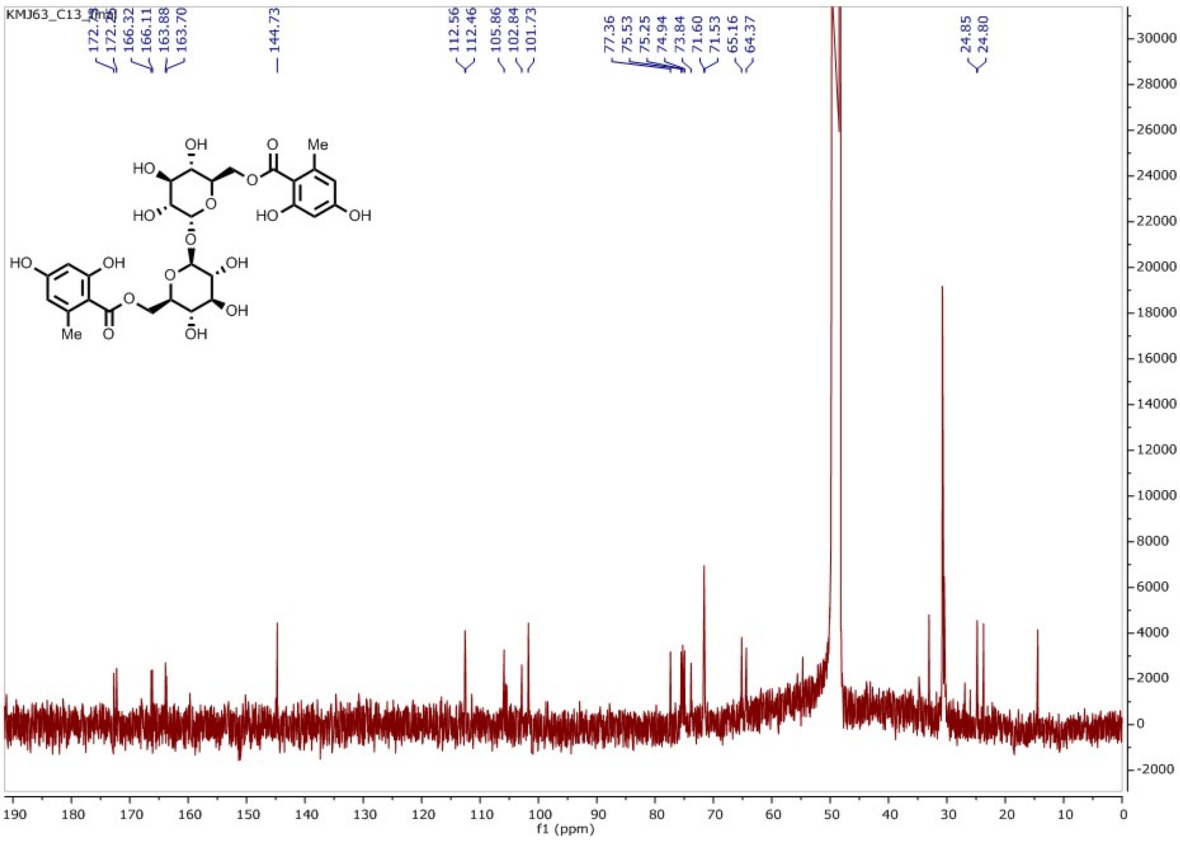

HPLC-UV-MS Chromatograms

The stock solutions of products in DMSO used for binding assays were analyzed on a Gilson GX-271/Perkin Elmer Flexar SQ-300 LC-MS system fitted with a Phenomenex Luna 5u CN 100A (150 x 4.6 mm) column and eluted with 5 % → 65 % MeCN in H_2O over 20 minutes, after 4 minutes the eluent was changed to 100 % MeCN. The elution was performed with a constant flow of 1 mL/min. Retention time of DMSO is 3.6 min.

6,6'-Bis-(2,4-dihydroxy-6-methylbenzoate)-α,α-trehalose (1)

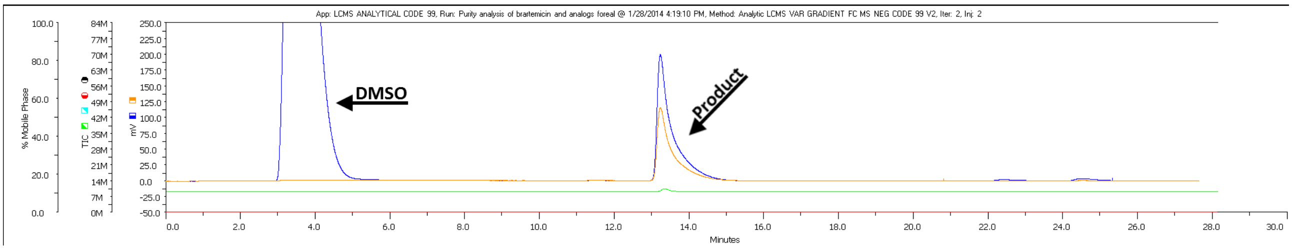

Retention time: 13.3 min.

6,6'-Bis-(2,3-dimethoxybenzoate)-α,α-trehalose (2)

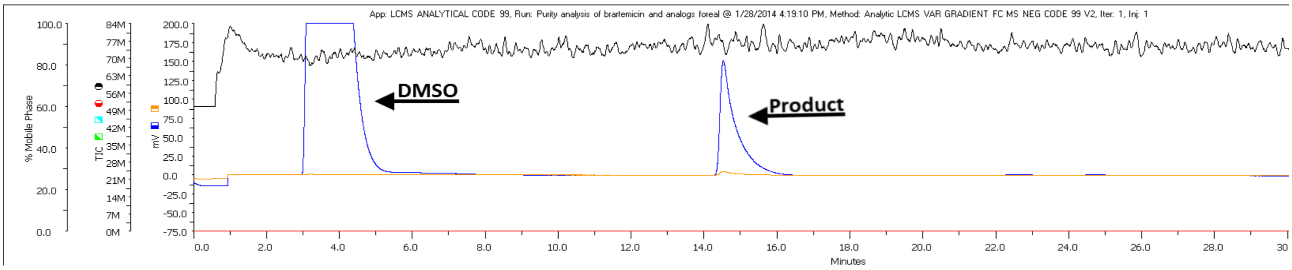

Retention time: 14.6 min.

6-(2,4-dihydroxy-6-methylbenzoate)-α,α-trehalose (4)

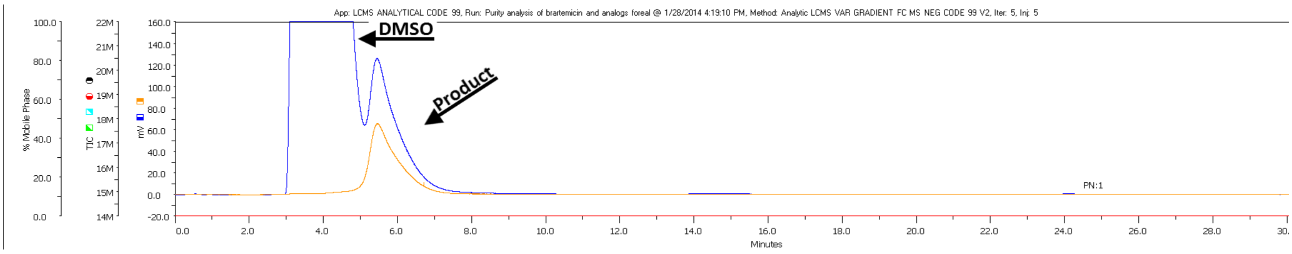

Retention time: 5.3 min.

6,6'-Bis-(benzoate)-α,α-trehalose (3)

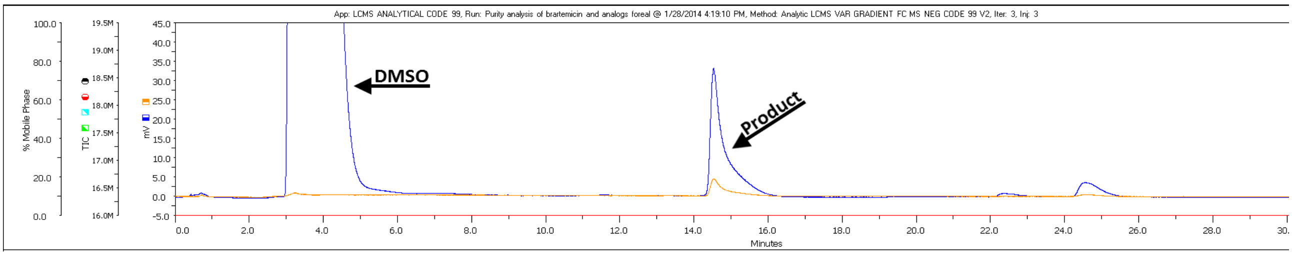

Retention time: 14.7 min.

6,6’-Bis-(2,4-dihydroxy-6-methylbenzoate)-α,β-trehalose (5)

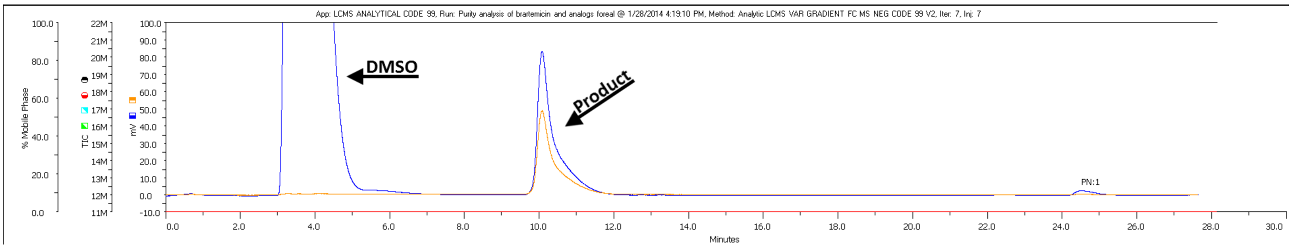

Retention time: 10.3 min.
